# Inflammatory Response-Related Long Non-Coding RNA Signature Predicts the Prognosis of Hepatocellular Carcinoma

**DOI:** 10.1155/2022/9917244

**Published:** 2022-03-17

**Authors:** Xinyu Li, Shuqiao Zhang, Shijun Zhang, Weihong Kuang, Chunzhi Tang

**Affiliations:** ^1^Medical College of Acupuncture-Moxibustion and Rehabilitation, Guangzhou University of Chinese Medicine, Guangzhou, Guangdong, China; ^2^First Affiliated Hospital of Guangzhou University of Chinese Medicine, Guangzhou University of Chinese Medicine, Guangzhou, Guangdong, China; ^3^Department of Traditional Chinese Medicine, The First Affiliated Hospital, Sun Yat-Sen University, Guangzhou, Guangdong, China; ^4^Guangdong Key Laboratory for Research and Development of Natural Drugs, School of Pharmacy, Guangdong Medical University, Dongguan, Guangdong, China

## Abstract

**Background:**

Hepatocellular carcinoma (HCC) is a high mortality malignant tumor with genetic and phenotypic heterogeneity, making predicting prognosis challenging. Meanwhile, the inflammatory response is an indispensable player in the tumorigenesis process and regulates the tumor microenvironment, which can affect the prognosis of tumor patients.

**Methods:**

Using HCC samples in the TCGA-LIHC dataset, we explored lncRNA expression profiles associated with the inflammatory response. The inflammatory response-related lncRNA signature was constructed by univariate Cox regression, LASSO regression, and multivariate Cox regression methods based on inflammatory response-related differentially expressed lncRNAs in HCC.

**Results:**

Seven inflammatory response-related lncRNA signatures were identified in predicting HCC prognosis. Kaplan–Meier (K-M) survival analysis indicated that high-risk group HCC patients were associated with poor prognosis. The utility of the inflammatory response-related lncRNA signatures was proved by the AUC and DCA analysis. The nomogram further confirmed the accuracy of the novel signature in predicting HCC patients' prognoses. In validation, our novel signature is more accurate than traditional clinicopathological performance for prognosis prediction of HCC patients. GSEA analysis further elucidated the underlying mechanisms and pathways of HCC progression in the low- and high-risk groups. Moreover, immune cells infiltration responses and immune function analyses revealed a significant difference between high- and low-risk groups in cytolytic activity, MHC class I, type I INF response, type II INF response, inflammation-promoting, and T cell coinhibition. Finally, HHLA2, NRP1, CD276, TNFRSF9, TNFSF4, CD80, and VTCN1 were expressed higher in high-risk groups in the immune checkpoint analysis.

**Conclusions:**

A novel inflammatory response-related lncRNA signature (AC145207.5, POLHAS1, AL928654.1, MKLN1AS, AL031985.3, PRRT3AS1, and AC023157.2) is capable of predicting the prognosis of HCC patients and providing new immune targeted therapies insight.

## 1. Introduction 

Hepatocellular carcinoma (HCC) is the primary histological subtype of liver cancer, with genetic and phenotypic heterogeneity, and the third most invasive and lethal tumor globally [1]. The inflammatory response caused by risk factors such as chronic hepatitis B, hepatitis C virus infection, smoking, obesity, and diabetes promotes liver fibrosis, which progresses to cirrhosis and ultimately to HCC [2–4]. HCC patients are asymptomatic early, which delays timely diagnosis. Patients who are only diagnosed at an advanced stage of liver cancer are not candidates for radical surgery, and treatment options are limited in availability and effectiveness [5]. Therefore, novel biomarkers to discriminate high-risk HCC patients are urgently needed to improve personalized liver cancer therapy.

Over the past few decades, research studies on tumor-associated inflammatory responses has increased rapidly, and inflammation is also regarded as one hallmark of cancer [6]. Many tumors arise from inflammatory responses, a critical component of the neoplastic process. The tumor microenvironment, primarily orchestrated by inflammatory cells, is indispensable in promoting tumor proliferation, survival, and migration [7]. Inflammatory response-related therapies induction holds promise as an opportunity to inhibit HCC development. Meanwhile, long non-coding RNA (lncRNA) is an endogenous cellular RNA molecule (>200 nucleotide sequences) that regulates gene expression and is involved in a variety of inflammatory biological pathways, including oncogenesis, development, and metastasis [8, 9]. Functional lncRNAs are considered to play a critical role in the process of inflammatory response regulation [10–13]. However, studies elucidating the mechanisms of inflammatory response-related lncRNAs in HCC progression remain scarce. A systematic evaluation of inflammatory response-related lncRNAs prognostic signature in liver patients may deepen our understanding of HCC progression mechanisms and offer novel approaches for specific, precise diagnosis and effective therapies.

In our study, a novel prognostic signature was first established based on inflammatory response-related differentially expressed lncRNAs. We then studied the roles of the novel lncRNA signature-associated mRNAs, immune responses, and N6-methylated adenosine (m6A) modification status in HCC prognosis.

## 2. Methods

### 2.1. Data Collection

RNA sequencing data with complete clinical information annotation were downloaded from the public database TCGA-LIHC (https://portal.gdc.cancer.gov/repository) dataset. Clinical information of HCC patients is shown in Table 1. The corresponding inflammatory response-related genes were identified from the Molecular Signatures (http://www.gsea-msigdb.org/gsea/login.jsp) database [14] and are provided in Table S1. Pearson's correlation analysis was used to identify inflammatory response-related lncRNAs by comparing the expression levels of inflammatory response-related genes and lncRNAs. Correlations are considered significant when the correlation coefficient |*R*^2^| > 0.4 and the *P* < 0.05, and then inflammatory response-related lncRNAs were selected. The vital differential expression of lncRNA associated with inflammatory response was set as |log_2_*f*_*c*_| ≥ 1.00 and false discovery rates (FDR) < 0.05. The biological functions, including biological process (BP), cellular component (CC), and molecular function (MF) of inflammatory response-associated differentially expressed lncRNAs (DEGs), were investigated using Gene Ontology (GO). And the pathways of differentially expressed inflammatory response-related lncRNAs involved in HCC progression were analyzed using the “clusterProfiler” package in R software (version 4.1.0) by the Kyoto Encyclopedia of Genes and Genomes (KEGG).

### 2.2. Development of the Inflammatory Response-Related lncRNAs Prognostic Signature

The inflammatory response-related DEGs significantly associated with the prognosis of HCC patients were first screened via univariate Cox regression analysis. Then, LASSO regression analysis was used to reduce the number of lncRNAs filtered by univariate Cox regression and prevent the risk model of overfitting. Finally, an inflammatory response-related lncRNA signature was constructed by multivariate Cox proportional hazards regression analysis, and the risk score formula stratified HCC patients. The formula of risk score model = ∑_*i*_^7^*x*_*i*_ × *y*_*i*_(*X* : coefficients,  *Y* : lncRNA expression level). Additionally, HCC patients were divided into high-risk and low-risk groups based on the median risk score.

### 2.3. The Predictive Nomogram

A hybrid nomogram model incorporating independent predictive factors including risk signature and gender, age, TMN, stage, and grade was established for predicting the 1-, 3-, and 5-year overall survival rate of HCC patients. Then, the fit degree of the calibration curve versus the actual observed value was used to judge the accuracy of the hybrid nomogram for clinical prognosis judgment.

### 2.4. Immune Profile Analysis

The single-sample gene set enrichment analysis (ssGSEA) proceeded to quantify the individual specimens' immune cell infiltration levels of low-risk and high-risk groups. The immune response differences between the two risk groups were assessed based on the results of multialgorithms including CIBERSORT [15, 16], CIBERSORT-ABS [17], QUANTISEQ [18], MCPCOUNTER [19], XCELL [20], EPIC [21], and TIMER [22]. In addition, the heatmap demonstrated the differences of immune responses in two risk groups stratified by inflammatory response-related lncRNA signatures under different algorithms. Moreover, the immune function of tumor-infiltrating immune cell subsets in the low-risk and high-risk groups was analyzed.

### 2.5. Statistical Analysis

**Figure 1:**
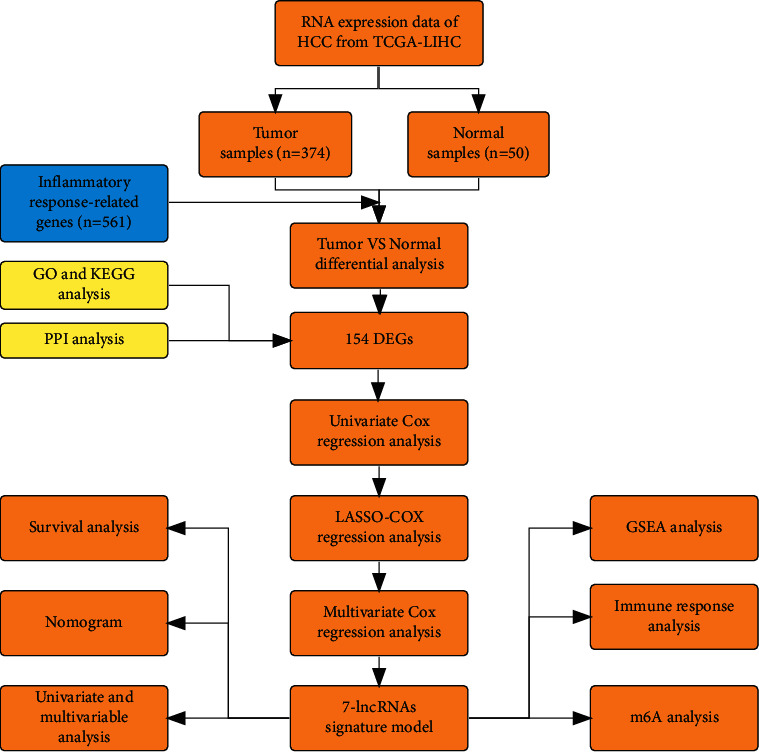
Flow chart of this study.

We used packages including “limma,” “survival,” and “survminer” in RStudio software (version 1.4.1106) for analyzing data. The Wilcoxon test and unpaired Student's *t*-test were used to compare nonnormal and normal distribution expression variables. Based on the FDR, the differential expressions of lncRNA were corrected by the Benjamin Hochberg method. The “GSVA” package in R was used to compare the ssGSEA-normalized HCC DEGs. We applied the time-dependent receiver operator characteristic (ROC) and the decision curve analysis (DCA) [23] to compare the performance between the inflammatory response-related lncRNA signature and clinical characteristics in predicting HCC prognosis. Furthermore, a clinical heatmap graph with Fisher's test was utilized to assess the relationship between inflammatory response-related lncRNAs and clinical characteristic manifestations. The overall survival of HCC patients was evaluated with a Kaplan–Meier (K–M) survival analysis based on the inflammatory response-related lncRNA signature. *P* < 0.05 was considered statistically significant in all analyses. The flow chart summarized this study in Figure 1.

## 3. Results

### 3.1. Enrichment Analysis of Inflammatory Response-Related Genes

**Figure 2:**
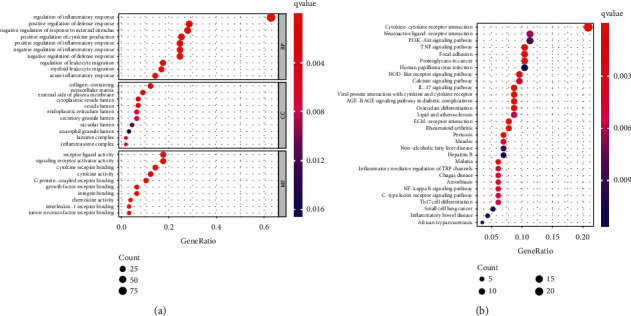
Go and KEGG enrichment analysis of inflammatory response-related DEGs. (a) GO analysis. (b) KEGG analysis. The inflammatory response-based lncRNA prognostic signature.

We identified 154 inflammatory response-related DEGs between HCC and noncancerous liver tissues (36 upregulated and 118 downregulated; Table S2). Enriched BP includes inflammatory response, positive regulation of defense response, and the immune system process. Meanwhile, the MF of DEGs in HCC were cytokine activity, cytokine receptor binding, and receptor ligand activity. The collagen-containing vesicle lumen, extracellular matrix, and the plasma membrane were predominantly enriched in CC. Additionally, pathways of DEGs analysis by KEGG indicated that the PI3K-AKT signaling pathway, the NOD-like receptor signaling pathway, focal adhesion, the TNF signaling pathway, the NF-kappa B signaling pathway, and proteoglycans in cancer were highly enriched in Figure 2.

**Figure 3:**
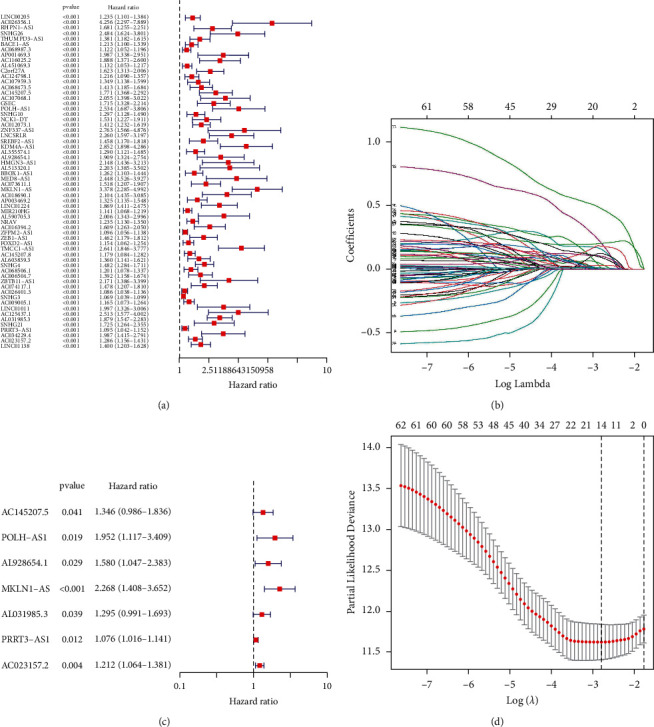
Development of inflammatory response-related lncRNA prognostic signature. (a) Univariate Cox regression analysis. (b, d) LASSO regression analysis. (c) Multivariate Cox regression analysis. Multivariate examination of the prognostic signature.

In the primary screening, 62 inflammatory response-related lncRNAs associated with HCC prognosis were obtained using univariate Cox analysis from differential expressed inflammatory response-related lncRNAs in HCC (Figure 3(a)). Next, LASSO regression was used to penalize 62 inflammatory response-related lncRNAs (Figures 3(b) and 3(d)). Finally, multivariate Cox regression analysis constructed seven inflammatory response-related lncRNA signatures as independent prognostic indicators for HCC patients (Figure 3(c); Table S3). Then, the novel risk score model was calculated by formula as follows: risk score = (coefficient AC145207.5 × expression of AC145207.5) + (coefficient POLH-AS1 × expression of POLH-AS1) + (coefficient AL928654.1 × expression AL928654.1) + (coefficient MKLN1-AS × expression MKLN1-AS) + (coefficient AL031985.3 ×expression AL031985.3) + (coefficient PRRT3-AS1 × expression PRRT3-AS1) + (coefficient AC023157.2 × expression AC023157.2).

**Figure 4:**
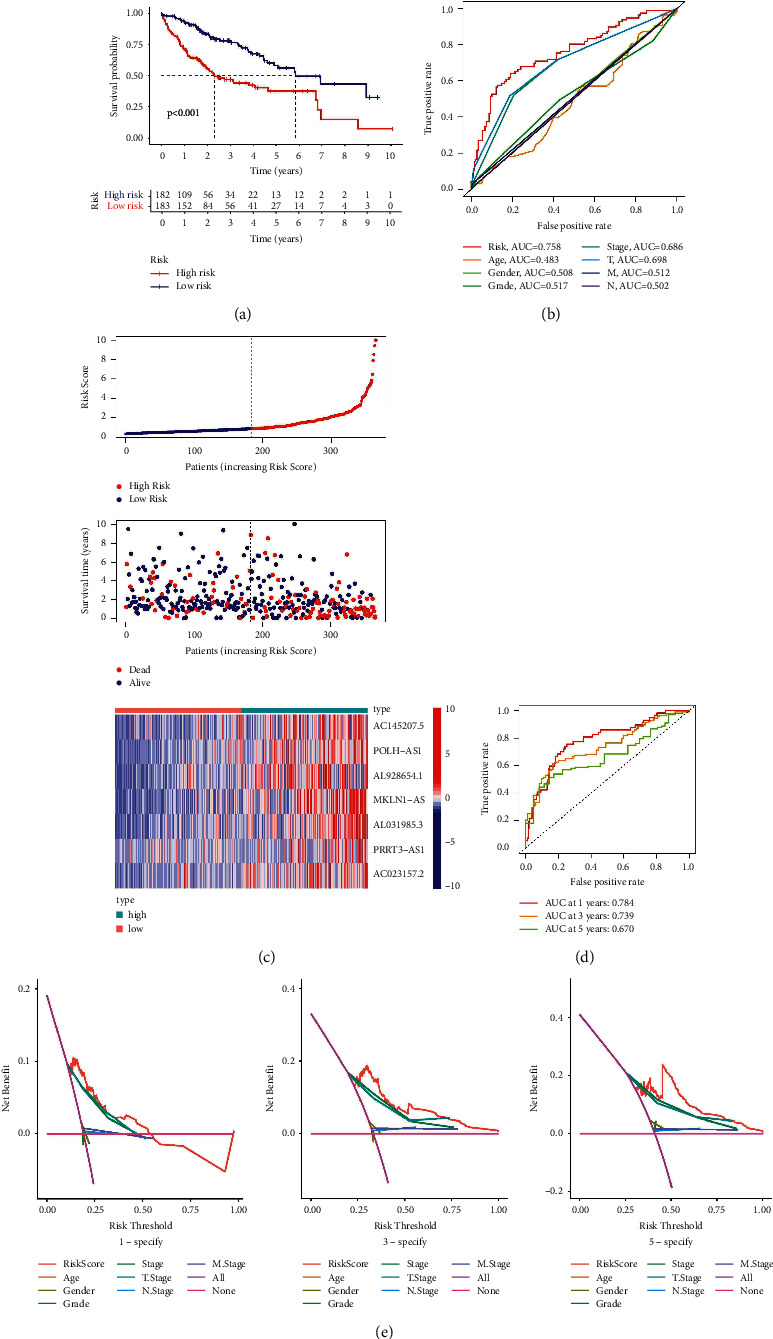
Validation of the seven inflammatory response-related lncRNAs signature. (a) K–M survival analysis result. (b) The AUC values for forecasting overall survival based on risk factors. (c) Survival status and the risk score distribution of HCC patients. (d) The AUC of the ROC curves represented 1-, 3-, and 5-year survival rate of HCC patients. (e) The DCA curves for the signature and clinicopathological manifestation.

**Figure 5:**
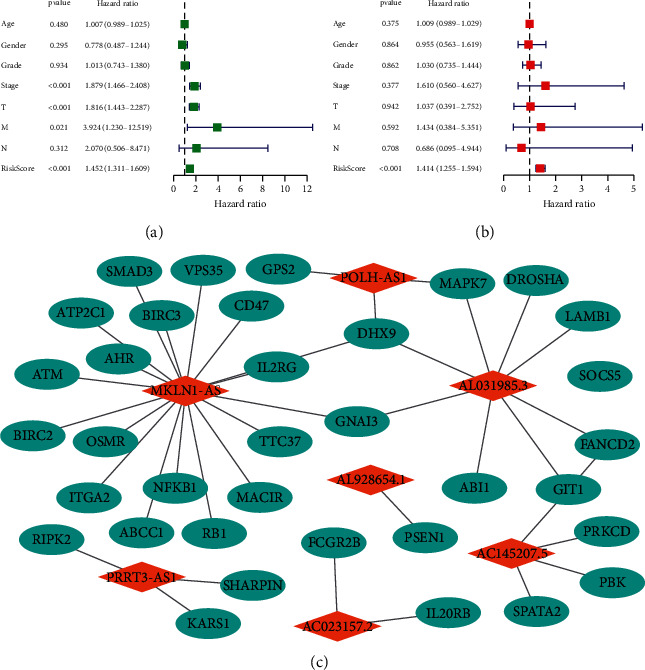
Assessment of the clinical prognostic value of the inflammatory response-related lncRNA signature in HCC patients based on univariate and multivariate COX analyses. (a) Univariate independent Cox analysis. (b) Multivariate independent Cox analysis. (c) The lncRNA-mRNA interaction.

**Figure 6:**
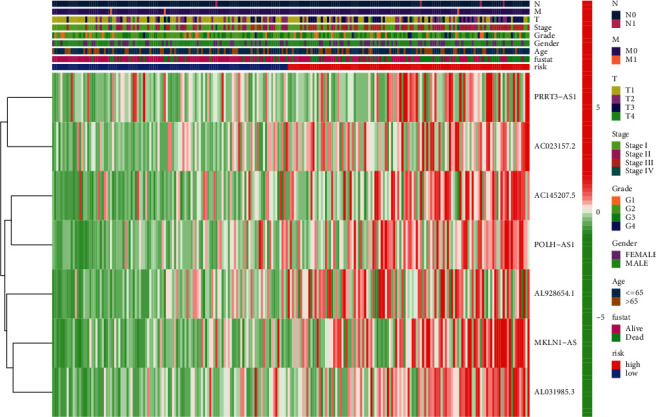
Heatmap of inflammatory response-related lncRNAs prognosis signature associated with clinical pathology.

**Figure 7:**
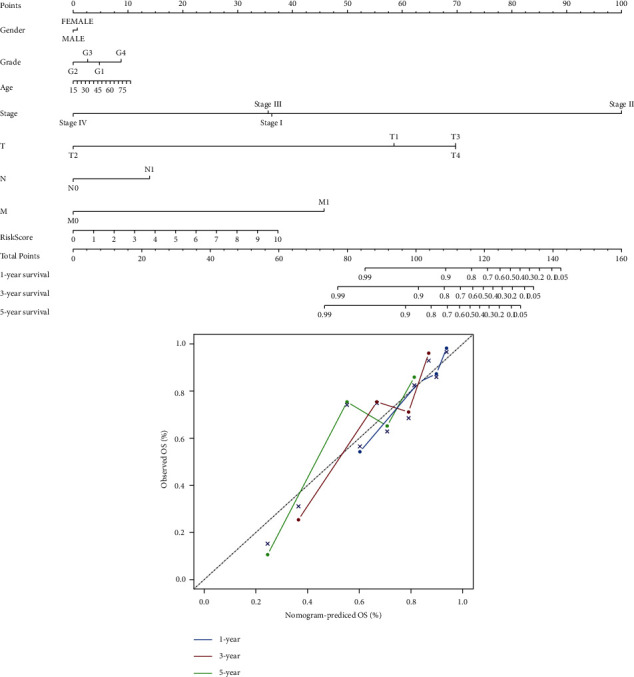
Nomogram and calibration curves constructed based on prognostic inflammatory response-related lncRNA signature and prognosis related clinicopathological factors.

Kaplan–Meier analysis confirmed that the high-risk group patients have worse overall survival than patients in the low-risk group (Figure 4(a)). Meanwhile, the inflammatory response-related lncRNAs signature had the AUC of 0.758, which outperformed traditional clinical characteristics in the prediction of prognosis of HCC patients (Figure 4(b)). From the risk survival status plots and heatmaps, it could be seen that a higher risk score is associated with a lower survival rate of patients with HCC (Figure 4(c)). The AUCs of ROC analysis was 0.784, 0.739, and 0.670 for the predictive value of HCC patients for 1-year, 3-year, and 5-year survival, respectively (Figure 4(d)). Besides, the net benefit of the DCA plot revealed a stable and robust prognostic-predictive ability of the inflammatory response-related lncRNA signature (Figure 4(e) and Table S4). Univariate and multivariate Cox analyses verified that the novel risk score model (HR: 1.41, 95 CI: 1.26–1.59) is an independent prognostic predictor of HCC patients' overall survival (Figures 5(a) and 5(b)). The inflammatory response-related lncRNA-mRNA interaction was presented in the correlation network (Figure 5(c)). Also, the clinical heatmap analyzed the relevance among the inflammatory response-related lncRNA signature and the clinicopathological manifestation (Figure 6). The calibration curves showed excellent uniformity between predicted overall survival and actual observed values with longer follow-up, which confirmed that the nomogram is reliable (Figure 7). Thus, this nomogram model is suitable for the clinical management of HCC patients.

### 3.2. Gene Set Enrichment Analysis

**Figure 8:**
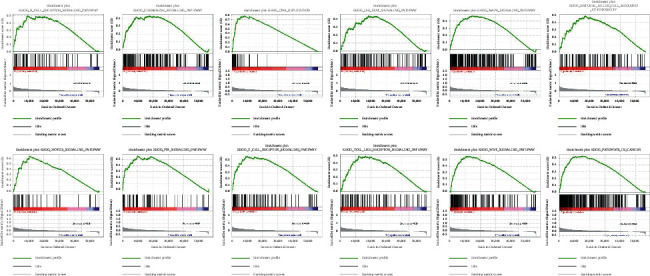
GSEA for inflammatory response-related lncRNAs signature.

The pathways and bioprocess involved in tumorigenesis were analyzed by GSEA, which revealed that the inflammatory response-related lncRNA signature modulated both the progression of tumor and the essential pathways associated with immunity, mainly including the JAK-STAT signaling pathway, the toll-like receptor signaling pathway, the WNT signaling pathway, the T cell receptor signaling pathway, the MAPK signaling pathway, the NOTCH signaling pathway, and natural killer cell-mediated cytotoxicity (Figure 8; Table S5).

### 3.3. Immunological Reaction and Related Gene Expression

**Figure 9:**
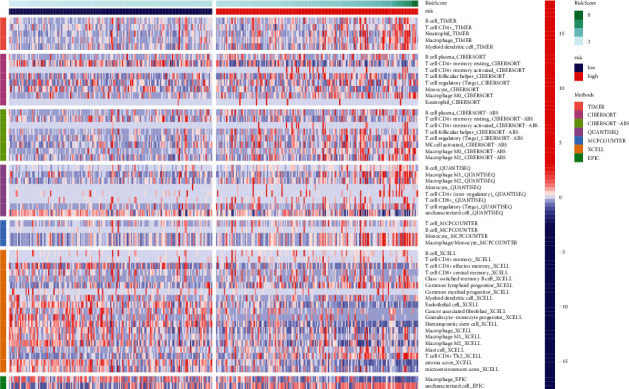
The immune responses of the inflammatory response-related lncRNA signature in two risk group.

**Figure 10:**
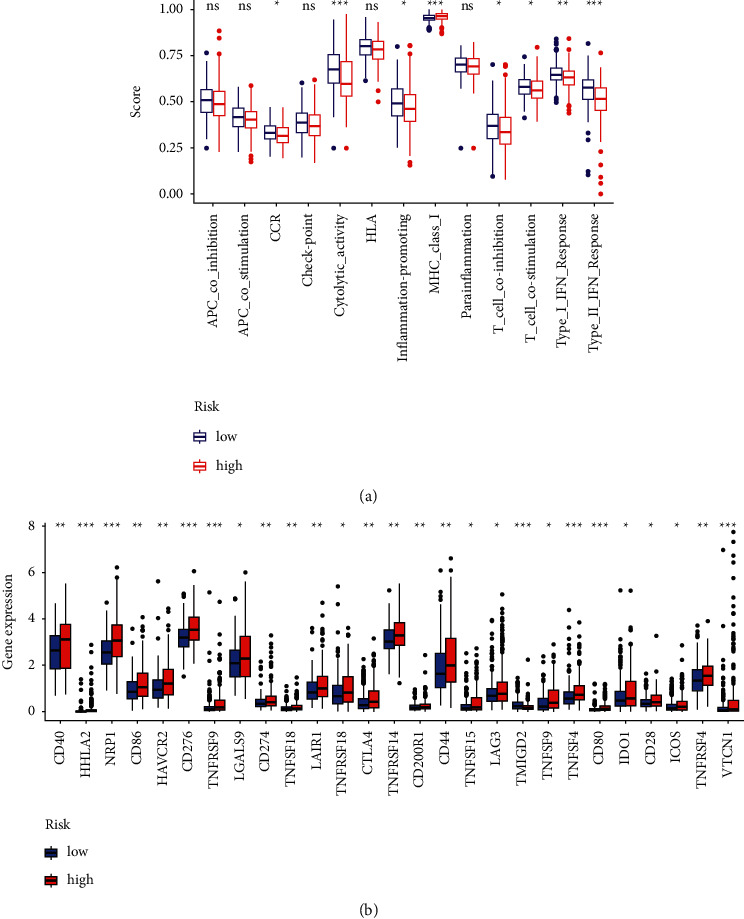
Immunity analyses and gene expression. (a) Relevant functional analysis of immune cell subsets. (b) Analyses of immune checkpoints between the two HCC risk groups.

**Figure 11:**
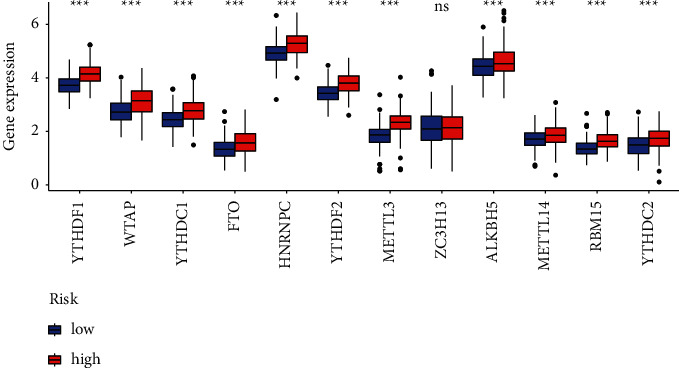
Analyses of m6A modification expression between the low and high HCC risk groups.

The heatmap discovered that the expression of immune responses was markedly different between low- and high-risk groups using multiple algorithms (Figure 9 and Table S6). Single-sample GSEA correlation analyses showed significant differences in the expression of corresponding immune functions between the low- and high-risk groups. In high-risk groups, immune functions such as T cell coinhibition and costimulation, type-II INF response, and T cell coinhibition were markedly attenuated (Figure 10(a)). Given the critical role of checkpoint inhibitors in HCC immunotherapy, we examined the differences in immune checkpoint expression between the two risk groups. In the high-risk group, immune checkpoints expression including HHLA2, NRP1, CD276, TNFRSF9, TMIGD2, TNFSF4, CD80, and VTCN1 were higher than in the low-risk group (Figure 10(b)). Expression comparison of m6A-related modification in two risk groups indicated that the high-risk group had higher expression of RNA methyltransferases (METTL3, METTL14, RBM15, and WTAP), demethylases (ALKBH5 and FTO), and readers (YTHDF1, YTHDF2, YTHDC1, YTHDC2, and HNRNPC) (Figure 11).

## 4. Discussion

The inflammatory response is critical in neoplastic progression by causing reactive oxygen species and deoxyribonucleic acid damage, increasing the frequency of genomic DNA mutations and causing oncogenesis [24, 25]. Meanwhile, inflammation-induced changes in the hepatic immune system make cancer cells prone to escape immune surveillance and destruction [6, 25]. We first identified 154 inflammatory response-related DEGs by comparing HCC and normal liver tissues. KEGG analysis discovered that these DEGs mainly participated in focal adhesion, proteoglycans in cancer, the PI3K-AKT signaling pathway, NF-kappa B and TNF signaling pathway. Some recent studies have shown that inflammatory interferon regulates cellular metastasis, vasculogenic mimicry, and antiapoptosis activity of tumor cells, mainly activating the PI3K/AKT/mTOR pathway [26]. At the same time, FGFR1 and TLR4 regulate tumor cell hyperplasia and migration and promote proinflammatory response production via the PI3K/Akt signaling pathway [27]. Studies by Balkwill [28], Ringelhan et al. [29], and Taniguchi et al. [30] reported that TNF*α* activated the NF-*κ*B signaling pathway, contributing to the promotion and progression of human HCC through hepatic inflammation, hepatocyte death, and compensatory proliferation.

Therefore, blocking the link between inflammation and liver cancer may inspire a new strategy for HCC treatment. In our study, the seven inflammatory response-related lncRNA signature based on clinical features were confirmed as independent prognostic factors for HCC patients. Among the seven inflammatory response-related lncRNA signatures, only a small part of lncRNAs have been reported to be studied. Zhou et al. [31] reported that AC145207.5 and AL031985.3 were overexpressed in HCC cell lines and were related to the poor prognosis of HCC patients. MKLN1-AS could intensify the hyperplasia, migration, and invasion of liver cancer cells by positively regulating YAP1 expression [32]. Li et al. [33] revealed that silencing of lncRNA PRRT3-AS1 could activate the expression of the PPAR*γ* gene and then block the mTOR signaling pathway to inhibit prostate cancer cell proliferation and promote apoptosis and autophagy. Although the other three lncRNAs have not been reported yet, according to the coexpression network, we found that POLH-AS1 has a coexpression relationship with GPS2, DHX9, and MAPK7. AL928654.1 has a coexpression relationship with PSEN1. AC023157.2 has a coexpression relationship with FCGR2B and IL20RB. From this, we speculated that these inflammatory response-related lncRNAs are likely to participate in the proliferation, migration, and immune response in cancer. However, the function of these inflammatory response-related lncRNAs and their roles in hepatocarcinogenesis and progression need to be explored through further clinical and experimental studies.

Then, the risk score model classified HCC patients into high- and low-risk groups. The survival analysis determined that patients in the high-risk group had a poor prognosis for HCC. Furthermore, the risk score model had an AUC of 0.758 and performed well with the net benefit of DCA validation. Moreover, the nomogram's calibration curves validated that our novel risk score model performs better than traditional clinicopathological characteristics in predicting the prognosis of HCC.

The direct correlation between lncRNAs and cancer-derived inflammatory responses emphasises their potential as tumor biomarkers and therapeutic targets [34]. Accumulating evidence suggests that lncRNAs are crucial in mediating inflammatory responses and dysregulation in HCC [35–38]. So lncRNAs could be the essential class of prevalent genes involved in the development of liver cancer. However, the biological and molecular mechanisms of lncRNAs in HCC are not fully understood. Therefore, this novel signature could help us further explore the roles of lncRNA in cancer. In our study, GSEA analyzed the immune and tumor-related pathways of the novel signature in individuals in high- and low-risk groups. Relevant immune function analysis indicated that the high-risk group's patients exhibited significantly reduced cytolytic activity, type II INF response, and T cell coinhibition. However, the high-risk group patients had increased expression of immune checkpoints including HHLA2, NRP1, CD276, TNFRSF9, and TNFSF4. Recently, lncRNAs have been gaining attention as critical regulators in gene expression and regulation via versatile interactivity with DNA, mRNA, or proteins. Notably, lncRNAs play vital roles in developing diverse immune cells by controlling dynamic transcriptional programs that are hallmarks of immune cell activation and inflammatory gene expression [39, 40]. Some studies have found that activation of inflammatory response pathways, such as the IFN response, can ameliorate sensitivity to immune checkpoint inhibitors in cancer patients and have a positive effect on antitumor activity [41], but also that lncRNA Mirt2 functions as a checkpoint to prevent aberrant activation of inflammation [42]. For now, few studies have delved into the association between inflammatory response and lncRNAs and immune checkpoint inhibitors. Thus, inflammatory response-related lncRNAs may be critical factors in the immune microenvironment causing HCC transformation.

Although we revealed a novel inflammatory response-related lncRNA prognostic risk signature and demonstrated the reliability of this risk model, our study has several limitations. This bioinformatics research needs to be confirmed by multicenter experiments with larger samples. Further exploration of the relationship between the seven inflammatory response-related lncRNAs in the model and immune activity deserves further exploration.

## 5. Conclusion

A specific inflammatory response-related lncRNA signature is capable of predicting the prognosis of HCC patients and providing new immune targeted therapies insight.

## Figures and Tables

**Table 1:** The clinical information of HCC patients in the TCGA-LIHC dataset.

Variable	Number of samples
Gender	
Male/female	255/122
Age at diagnosis	
≤65/>65/NA	235/141/1
Grade	
G1/G2/G3/G4/NA	55/180/124/13/5
Stage	
I/II/III/IV/NA	175/87/86/5/24
T	
T1/T2/T3/T4/NA	185/95/81/13/3
M	
M0/M1/NA	272/4/101
N	
N0/N1/NA	257/4/116

## Data Availability

The data used to support the findings of this study are available from the corresponding author upon request.

## Abbreviation

HCC: Hepatocellular carcinoma
TCGA: The Cancer Genome Atlas
GO: Gene Ontology
BP: Biological processes
MF: Molecular function
CC: Cellular components
KEGG: Kyoto Encyclopedia of Genes and Genomes
GSEA: Gene set enrichment analyses
FDR: False discovery rate
SsGSEA: Single-sample gene set enrichment analysis.

## Supplementary Materials

Supplementary Materials Supplementary tables: Supplementary Table 1. Identified inflammatory response-related genes from the Molecular Signatures Database. Supplementary Table 2. The inflammatory response-related DEGs between HCC and noncancerous liver tissues. Supplementary Table 3. The results of univariate Cox regression, LASSO regression, and multivariate Cox regression analysis. Supplementary Table 4. The net benefit of risk score model in DCA analysis. Supplementary Table 5. The results of gene set enrichment analysis. Supplementary Table 6. The immune responses in low- and high-risk groups.
