# Supplementary material for: Inflammatory Response-Related Long Non-Coding RNA Signature Predicts the Prognosis of Hepatocellular Carcinoma
Source: J Oncol. 2022 Mar 17;2022:9917244. doi: 10.1155/2022/9917244 (PMC8947866; doi:10.1155/2022/9917244)
Supplement: Supplementary Materials — Supplementary tables: Supplementary Table 1. Identified inflammatory response-related genes from the Molecular Signatures Database. Supplementary Table 2. The inflammatory response-related DEGs between HCC and noncancerous liver tissues. Supplementary Table 3. The results of univariate Cox regression, LASSO regression, and multivariate Cox regression analysis. Supplementary Table 4. The net benefit of risk score model in DCA analysis. Supplementary Table 5. The results of gene set enrichment analysis. Supplementary Table 6. The immune responses in low- and high-risk groups. [file 9917244.f1.zip › 9917244.f1/Table S1 (1).pdf]

Inflammatory response-related genes	Full names
CD28	T-cell-specific surface glycoprotein CD28 (TP44) (CD antigen CD28)
CD40	Tumor necrosis factor receptor superfamily member 5 (B-cell surface antigen CD40) (Bp50) (CD40L receptor) (CDw40) (CD antigen CD40)
CD40LG	CD40 ligand (CD40-L) (T-cell antigen Gp39) (TNF-related activation protein) (TRAP) (Tumor necrosis factor ligand superfamily member 5) (CD antigen CD154) [Cleaved into: CD40 ligand, membrane form; CD40 ligand, soluble form (sCD40L)]
CD80	T-lymphocyte activation antigen CD80 (Activation B7-1 antigen) (BB1) (CTLA-4 counter-receptor B7.1) (B7) (CD antigen CD80)
CD86	T-lymphocyte activation antigen CD86 (Activation B7-2 antigen) (B70) (BU63) (CTLA-4 counter-receptor B7.2) (FUN-1) (CD antigen CD86)
COL1A1	Collagen alpha-1(I) chain (Alpha-1 type I collagen)
COL1A2	Collagen alpha-2(I) chain (Alpha-2 type I collagen)
COL3A1	Collagen alpha-1(III) chain
FN1	Fibronectin (FN) (Cold-insoluble globulin) (CIG) [Cleaved into: Anastellin; Ugl-Y1; Ugl-Y2; Ugl-Y3]
IFNG	Interferon gamma (IFN-gamma) (Immune interferon)
IL2	Interleukin-2 (IL-2) (T-cell growth factor) (TCGF) (Aldesleukin)
IL2RA	Interleukin-2 receptor subunit alpha (IL-2 receptor subunit alpha) (IL-2-RA) (IL-2R subunit alpha) (IL2-RA) (TAC antigen) (p55) (CD antigen CD25)
IL2RB	Interleukin-2 receptor subunit beta (IL-2 receptor subunit beta) (IL-2R subunit beta) (IL-2RB) (High affinity IL-2 receptor subunit beta) (Interleukin-15 receptor subunit beta) (p70-75) (p75) (CD antigen CD122)
IL2RG	Cytokine receptor common subunit gamma (Interleukin-2 receptor subunit gamma) (IL-2 receptor subunit gamma) (IL-2R subunit gamma) (IL-2RG) (gammaC) (p64) (CD antigen CD132)
IL4	Interleukin-4 (IL-4) (B-cell stimulatory factor 1) (BSF-1) (Binetrakin) (Lymphocyte stimulatory factor 1) (Pitrakinra)
IL4R	Interleukin-4 receptor subunit alpha (IL-4 receptor subunit alpha) (IL-4R subunit alpha) (IL-4R-alpha) (IL-4RA) (CD antigen CD124) [Cleaved into: Soluble interleukin-4 receptor subunit alpha (Soluble IL-4 receptor subunit alpha) (Soluble IL-4R-alpha) (sIL4Ralpha/prot) (IL-4-binding protein) (IL4-BP)]
IL5	Interleukin-5 (IL-5) (B-cell differentiation factor I) (Eosinophil differentiation factor) (T-cell replacing factor) (TRF)
IL5RA	Interleukin-5 receptor subunit alpha (IL-5 receptor subunit alpha) (IL-5R subunit alpha) (IL-5R-alpha) (IL-5RA) (CDw125) (CD antigen CD125)

LAMA5	Laminin subunit alpha-5 (Laminin-10 subunit alpha) (Laminin-11 subunit alpha) (Laminin-15 subunit alpha)
LAMB1	Laminin subunit beta-1 (Laminin B1 chain) (Laminin-1 subunit beta) (Laminin-10 subunit beta) (Laminin-12 subunit beta) (Laminin-2 subunit beta) (Laminin-6 subunit beta) (Laminin-8 subunit beta)
LAMB2	Laminin subunit beta-2 (Laminin B1s chain) (Laminin-11 subunit beta) (Laminin-14 subunit beta) (Laminin-15 subunit beta) (Laminin-3 subunit beta) (Laminin-4 subunit beta) (Laminin-7 subunit beta) (Laminin-9 subunit beta) (S-laminin subunit beta) (S-LAM beta)
LAMB2,LAMC1	Laminin subunit gamma-1 (Laminin B2 chain) (Laminin-1 subunit gamma) (Laminin-10 subunit gamma) (Laminin-11 subunit gamma) (Laminin-2 subunit gamma) (Laminin-3 subunit gamma) (Laminin-4 subunit gamma) (Laminin-6 subunit gamma) (Laminin-7 subunit gamma) (Laminin-8 subunit gamma) (Laminin-9 subunit gamma) (S-laminin subunit gamma) (S-LAM gamma)
LAMC1	Laminin, gamma 1 (Formerly LAMB2), isoform CRA_a
LAMC2	Laminin subunit gamma-2 (Cell-scattering factor 140 kDa subunit) (CSF 140 kDa subunit) (Epiligrin subunit gamma) (Kalinin subunit gamma) (Kalinin/nicein/epiligrin 100 kDa subunit) (Ladsin 140 kDa subunit) (Laminin B2t chain) (Laminin-5 subunit gamma) (Large adhesive scatter factor 140 kDa subunit) (Nicein subunit gamma)
LCK	Tyrosine-protein kinase Lck (EC 2.7.10.2) (Leukocyte C-terminal Src kinase) (LSK) (Lymphocyte cell-specific protein-tyrosine kinase) (Protein YT16) (Proto-oncogene Lck) (T cell-specific protein-tyrosine kinase) (p56-LCK)
THBS1	Thrombospondin-1 (Glycoprotein G)
THBS3	Thrombospondin-3
TNFRSF1A	Tumor necrosis factor receptor superfamily member 1A (Tumor necrosis factor receptor 1) (TNF-R1) (Tumor necrosis factor receptor type I) (TNF-RI) (TNFR-I) (p55) (p60) (CD antigen CD120a) [Cleaved into: Tumor necrosis factor receptor superfamily member 1A, membrane form; Tumor necrosis factor-binding protein 1 (TBPI)]
TNFRSF1B	Tumor necrosis factor receptor superfamily member 1B (Tumor necrosis factor receptor 2) (TNF-R2) (Tumor necrosis factor receptor type II) (TNF-RII) (TNFR-II) (p75) (p80 TNF-alpha receptor) (CD antigen CD120b) (Etanercept) [Cleaved into: Tumor necrosis factor receptor superfamily member 1b, membrane form; Tumor necrosis factor-binding protein 2 (TBP-2) (TBPII)]
VTN	Vitronectin (VN) (S-protein) (Serum-spreading factor) (V75) [Cleaved into: Vitronectin V65 subunit; Vitronectin V10 subunit; Somatomedin-B]
ZAP70	Tyrosine-protein kinase ZAP-70 (EC 2.7.10.2) (70 kDa zeta-chain associated protein) (Syk-related tyrosine kinase)

ABCF1	ATP-binding cassette sub-family F member 1 (ATP-binding cassette 50) (TNF-alpha-stimulated ABC protein)
ADORA1	Adenosine receptor A1
ADORA2A	Adenosine receptor A2a
AGER	Advanced glycosylation end product-specific receptor (Receptor for advanced glycosylation end products)
AIF1	Allograft inflammatory factor 1 (AIF-1) (Ionized calcium-binding adapter molecule 1) (Protein G1)
ALOX15	Polyunsaturated fatty acid lipoxygenase ALOX15 (12/15-lipoxygenase) (Arachidonate 12-lipoxygenase, leukocyte-type) (12-LOX) (EC 1.13.11.31) (Arachidonate 15-lipoxygenase) (15-LOX) (15-LOX-1) (EC 1.13.11.33) (Arachidonate omega-6 lipoxygenase) (Hepoxilin A3 synthase Alox15) (EC 1.13.11.-) (Linoleate 13S-lipoxygenase) (EC 1.13.11.12)
ALOX5	Polyunsaturated fatty acid 5-lipoxygenase (EC 1.13.11.-) (Arachidonate 5-lipoxygenase) (5-LO) (5-lipoxygenase) (EC 1.13.11.34)
ALOX5AP	Arachidonate 5-lipoxygenase-activating protein (FLAP) (MK-886-binding protein)
ANXA1	Annexin A1 (Annexin I) (Annexin-1) (Calpactin II) (Calpactin-2) (Chromobindin-9) (Lipocortin I) (Phospholipase A2 inhibitory protein) (p35)
AOAH	Acyloxyacyl hydrolase (EC 3.1.1.77) [Cleaved into: Acyloxyacyl hydrolase small subunit; Acyloxyacyl hydrolase large subunit]
AOC3	Membrane primary amine oxidase (EC 1.4.3.21) (Copper amine oxidase) (HPAO) (Semicarbazide-sensitive amine oxidase) (SSAO) (Vascular adhesion protein 1) (VAP-1)
AOX1	Aldehyde oxidase (EC 1.2.3.1) (Aldehyde oxidase 1) (Azaheterocycle hydroxylase) (EC 1.17.3.-)
BCL6	B-cell lymphoma 6 protein (BCL-6) (B-cell lymphoma 5 protein) (BCL-5) (Protein LAZ-3) (Zinc finger and BTB domain-containing protein 27) (Zinc finger protein 51)
BDKRB2	B2 bradykinin receptor (B2R) (BK-2 receptor)
C3	Complement C3 (C3 and PZP-like alpha-2-macroglobulin domain-containing protein 1) [Cleaved into: Complement C3 beta chain; C3-beta-c (C3bc); Complement C3 alpha chain; C3a anaphylatoxin; Acylation stimulating protein (ASP) (C3adesArg); Complement C3b alpha' chain; Complement C3c alpha' chain fragment 1; Complement C3dg fragment; Complement C3g fragment; Complement C3d fragment; Complement C3f fragment; Complement C3c alpha' chain fragment 2]
C3AR1	C3a anaphylatoxin chemotactic receptor (C3AR) (C3a-R)
C4B	Complement C4-B (Basic complement C4) (C3 and PZP-like alpha-2-macroglobulin domain-containing protein 3) [Cleaved into: Complement C4 beta chain; Complement C4-B alpha chain; C4a anaphylatoxin; C4b-B; C4d-B; Complement C4 gamma chain]

C5	Complement C5 (C3 and PZP-like alpha-2-macroglobulin domain-containing protein 4) [Cleaved into: Complement C5 beta chain; Complement C5 alpha chain; C5a anaphylatoxin; Complement C5 alpha' chain]
CCL11	Eotaxin (C-C motif chemokine 11) (Eosinophil chemotactic protein) (Small-inducible cytokine A11)
CCL13	C-C motif chemokine 13 (CK-beta-10) (Monocyte chemoattractant protein 4) (Monocyte chemotactic protein 4) (MCP-4) (NCC-1) (Small-inducible cytokine A13) [Cleaved into: C-C motif chemokine 13, long chain; C-C motif chemokine 13, medium chain; C-C motif chemokine 13, short chain]
CCL17	C-C motif chemokine 17 (CC chemokine TARC) (Small-inducible cytokine A17) (Thymus and activation-regulated chemokine)
CCL18	C-C motif chemokine 18 (Alternative macrophage activation-associated CC chemokine 1) (AMAC-1) (CC chemokine PARC) (Dendritic cell chemokine 1) (DC-CK1) (Macrophage inflammatory protein 4) (MIP-4) (Pulmonary and activation-regulated chemokine) (Small-inducible cytokine A18) [Cleaved into: CCL18(1-68); CCL18(3-69); CCL18(4-69)]
CCL19	C-C motif chemokine 19 (Beta-chemokine exodus-3) (CK beta-11) (Epstein-Barr virus-induced molecule 1 ligand chemokine) (EBI1 ligand chemokine) (ELC) (Macrophage inflammatory protein 3 beta) (MIP-3-beta) (Small-inducible cytokine A19)
CCL2	C-C motif chemokine 2 (HC11) (Monocyte chemoattractant protein 1) (Monocyte chemotactic and activating factor) (MCAF) (Monocyte chemotactic protein 1) (MCP-1) (Monocyte secretory protein JE) (Small-inducible cytokine A2)
CCL20	C-C motif chemokine 20 (Beta-chemokine exodus-1) (CC chemokine LARC) (Liver and activation-regulated chemokine) (Macrophage inflammatory protein 3 alpha) (MIP-3-alpha) (Small-inducible cytokine A20) [Cleaved into: CCL20(1-67); CCL20(1-64); CCL20(2-70)]
CCL21	C-C motif chemokine 21 (6Ckine) (Beta-chemokine exodus-2) (Secondary lymphoid-tissue chemokine) (SLC) (Small-inducible cytokine A21)
CCL23	C-C motif chemokine 23 (CK-beta-8) (CKB-8) (Macrophage inflammatory protein 3) (MIP-3) (Myeloid progenitor inhibitory factor 1) (MPIF-1) (Small-inducible cytokine A23) [Cleaved into: CCL23(19-99); CCL23(22-99); CCL23(27-99); CCL23(30-99)]
CCL3	C-C motif chemokine 3 (G0/G1 switch regulatory protein 19-1) (Macrophage inflammatory protein 1-alpha) (MIP-1-alpha) (PAT 464.1) (SIS-beta) (Small-inducible cytokine A3) (Tonsillar lymphocyte LD78 alpha protein) [Cleaved into: MIP-1-alpha(4-69) (LD78-alpha(4-69))]

CCL4	C-C motif chemokine 4 (G-26 T-lymphocyte-secreted protein) (HC21) (Lymphocyte activation gene 1 protein) (LAG-1) (MIP-1-beta(1-69)) (Macrophage inflammatory protein 1-beta) (MIP-1-beta) (PAT 744) (Protein H400) (SIS-gamma) (Small-inducible cytokine A4) (T-cell activation protein 2) (ACT-2) [Cleaved into: MIP-1-beta(3-69)]
CCL7	C-C motif chemokine 7 (Monocyte chemoattractant protein 3) (Monocyte chemotactic protein 3) (MCP-3) (NC28) (Small-inducible cytokine A7)
CCL8	C-C motif chemokine 8 (HC14) (Monocyte chemoattractant protein 2) (Monocyte chemotactic protein 2) (MCP-2) (Small-inducible cytokine A8) [Cleaved into: MCP-2(6-76)]
CCR1	C-C chemokine receptor type 1 (C-C CKR-1) (CC-CKR-1) (CCR-1) (CCR1) (HM145) (LD78 receptor) (Macrophage inflammatory protein 1-alpha receptor) (MIP-1alpha-R) (RANTES-R) (CD antigen CD191)
CCR2	C-C chemokine receptor type 2 (C-C CKR-2) (CC-CKR-2) (CCR-2) (CCR2) (Monocyte chemoattractant protein 1 receptor) (MCP-1-R) (CD antigen CD192)
CCR5	C-C chemokine receptor type 5 (C-C CKR-5) (CC-CKR-5) (CCR-5) (CCR5) (CHEMR13) (HIV-1 fusion coreceptor) (CD antigen CD195)
CD14	Monocyte differentiation antigen CD14 (Myeloid cell-specific leucine-rich glycoprotein) (CD antigen CD14) [Cleaved into: Monocyte differentiation antigen CD14, urinary form; Monocyte differentiation antigen CD14, membrane-bound form]
CEBPB	CCAAT/enhancer-binding protein beta (C/EBP beta) (Liver activator protein) (LAP) (Liver-enriched inhibitory protein) (LIP) (Nuclear factor NF-IL6) (Transcription factor 5) (TCF-5)
CX3CL1	Fractalkine (C-X3-C motif chemokine 1) (CX3C membrane-anchored chemokine) (Neurotactin) (Small-inducible cytokine D1) [Cleaved into: Processed fractalkine]
CXCL1	Growth-regulated alpha protein (C-X-C motif chemokine 1) (GRO-alpha(1-73)) (Melanoma growth stimulatory activity) (MGSA) (Neutrophil-activating protein 3) (NAP-3) [Cleaved into: GRO-alpha(4-73); GRO-alpha(5-73); GRO-alpha(6-73)]
CXCL10	C-X-C motif chemokine 10 (10 kDa interferon gamma-induced protein) (Gamma-IP10) (IP-10) (Small-inducible cytokine B10) [Cleaved into: CXCL10(1-73)]
CXCL11	C-X-C motif chemokine 11 (Beta-R1) (H174) (Interferon gamma-inducible protein 9) (IP-9) (Interferon-inducible T-cell alpha chemoattractant) (I-TAC) (Small-inducible cytokine B11)
CXCL12	Stromal cell-derived factor 1 (SDF-1) (hSDF-1) (C-X-C motif chemokine 12) (Intercrine reduced in hepatomas) (IRH) (hIRH) (Pre-B cell growth-stimulating factor) (PBSF) [Cleaved into: SDF-1-beta(3-72); SDF-1-alpha(3-67)]

CXCL13	C-X-C motif chemokine 13 (Angie) (B cell-attracting chemokine 1) (BCA-1) (B lymphocyte chemoattractant) (CXC chemokine BLC) (Small-inducible cytokine B13)
CXCL14	C-X-C motif chemokine 14 (Chemokine BRAK) (MIP-2G) (Small-inducible cytokine B14)
CXCL2	C-X-C motif chemokine 2 (Growth-regulated protein beta) (Gro-beta) (Macrophage inflammatory protein 2-alpha) (MIP2-alpha) [Cleaved into: GRO-beta(5-73) (GRO-beta-T) (Hematopoietic synergistic factor) (HSF) (SB-251353)]
CXCL5	C-X-C motif chemokine 5 (ENA-78(1-78)) (Epithelial-derived neutrophil-activating protein 78) (Neutrophil-activating peptide ENA-78) (Small-inducible cytokine B5) [Cleaved into: ENA-78(8-78); ENA-78(9-78)]
CXCL6	C-X-C motif chemokine 6 (Chemokine alpha 3) (CKA-3) (Granulocyte chemotactic protein 2) (GCP-2) (Small-inducible cytokine B6) [Cleaved into: Small-inducible cytokine B6, N-processed variant 1; Small-inducible cytokine B6, N-processed variant 2; Small-inducible cytokine B6, N-processed variant 3]
CXCL8	Interleukin-8 (IL-8) (C-X-C motif chemokine 8) (Chemokine (C-X-C motif) ligand 8) (Emoctakin) (Granulocyte chemotactic protein 1) (GCP-1) (Monocyte-derived neutrophil chemotactic factor) (MDNCF) (Monocyte-derived neutrophil-activating peptide) (MONAP) (Neutrophil-activating protein 1) (NAP-1) (Protein 3-10C) (T-cell chemotactic factor) [Cleaved into: MDNCF-a (GCP/IL-8 protein IV) (IL8/NAP1 form I); Interleukin-8 ((Ala-IL-8)77) (GCP/IL-8 protein II) (IL-8(1-77)) (IL8/NAP1 form II) (MDNCF-b); IL-8(5-77); IL-8(6-77) ((Ser-IL-8)72) (GCP/IL-8 protein I) (IL8/NAP1 form III) (Lymphocyte-derived neutrophil-activating factor) (LYNAP) (MDNCF-c) (Neutrophil-activating factor) (NAF); IL-8(7-77) (GCP/IL-8 protein V) (IL8/NAP1 form IV); IL-8(8-77) (GCP/IL-8 protein VI) (IL8/NAP1 form V); IL-8(9-77) (GCP/IL-8 protein III) (IL8/NAP1 form VI)]
CXCL9	C-X-C motif chemokine 9 (Gamma-interferon-induced monokine) (Monokine induced by interferon-gamma) (HuMIG) (MIG) (Small-inducible cytokine B9)
CXCR2	C-X-C chemokine receptor type 2 (CXC-R2) (CXCR-2) (CDw128b) (GRO/MGSA receptor) (High affinity interleukin-8 receptor B) (IL-8R B) (IL-8 receptor type 2) (CD antigen CD182)
CXCR4	C-X-C chemokine receptor type 4 (CXC-R4) (CXCR-4) (FB22) (Fusin) (HM89) (LCR1) (Leukocyte-derived seven transmembrane domain receptor) (LESTR) (Lipopolysaccharide-associated protein 3) (LAP-3) (LPS-associated protein 3) (NPYRL) (Stromal cell-derived factor 1 receptor) (SDF-1 receptor) (CD antigen CD184)
CYBB	Cytochrome b-245 heavy chain (EC 1.-.-.-) (CGD91-phox) (Cytochrome b(558) subunit beta) (Cytochrome b558 subunit beta) (Heme-binding membrane glycoprotein gp91phox) (NADPH

	oxidase 2) (Neutrophil cytochrome b 91 kDa polypeptide) (Superoxide-generating NADPH oxidase heavy chain subunit) (gp91-1) (gp91-phox) (p22 phagocyte B-cytochrome)
FOS	Proto-oncogene c-Fos (Cellular oncogene fos) (G0/G1 switch regulatory protein 7)
FPR1	fMet-Leu-Phe receptor (fMLP receptor) (N-formyl peptide receptor) (FPR) (N-formylpeptide chemoattractant receptor)
FPR2	N-formyl peptide receptor 2 (FMLP-related receptor I) (FMLP-R-I) (Formyl peptide receptor-like 1) (HM63) (Lipoxin A4 receptor) (LXA4 receptor) (RFP)
IL1A	Interleukin-1 alpha (IL-1 alpha) (Hematopoietin-1)
IL1B	Interleukin-1 beta (IL-1 beta) (Catabolin)
IL1R1	Interleukin-1 receptor type 1 (IL-1R-1) (IL-1RT-1) (IL-1RT1) (EC 3.2.2.6) (CD121 antigen-like family member A) (Interleukin-1 receptor alpha) (IL-1R-alpha) (Interleukin-1 receptor type I) (p80) (CD antigen CD121a) [Cleaved into: Interleukin-1 receptor type 1, membrane form (mIL-1R1) (mIL-1RI); Interleukin-1 receptor type 1, soluble form (sIL-1R1) (sIL-1RI)]
IL1RAP	Interleukin-1 receptor accessory protein (IL-1 receptor accessory protein) (IL-1RAcP) (EC 3.2.2.6) (Interleukin-1 receptor 3) (IL-1R-3) (IL-1R3)
IL9	Interleukin-9 (IL-9) (Cytokine P40) (T-cell growth factor P40)
LY75	Lymphocyte antigen 75 (Ly-75) (C-type lectin domain family 13 member B) (DEC-205) (gp200-MR6) (CD antigen CD205)
LY86	Lymphocyte antigen 86 (Ly-86) (Protein MD-1)
LYZ	Lysozyme C (EC 3.2.1.17) (1,4-beta-N-acetylmuramidase C)
MGLL	Monoglyceride lipase (MGL) (EC 3.1.1.23) (HU-K5) (Lysophospholipase homolog) (Lysophospholipase-like) (Monoacylglycerol lipase) (MAGL)
NFATC4	Nuclear factor of activated T-cells, cytoplasmic 4 (NF-ATc4) (NFATc4) (T-cell transcription factor NFAT3) (NF-AT3)
NFKB1	Nuclear factor NF-kappa-B p105 subunit (DNA-binding factor KBF1) (EBP-1) (Nuclear factor of kappa light polypeptide gene enhancer in B-cells 1) [Cleaved into: Nuclear factor NF-kappa-B p50 subunit]
NMI	N-myc-interactor (Nmi) (N-myc and STAT interactor)
ORM1	Alpha-1-acid glycoprotein 1 (AGP 1) (Orosomucoid-1) (OMD 1)
PLA2G7	Platelet-activating factor acetylhydrolase (PAF acetylhydrolase) (EC 3.1.1.47) (1-alkyl-2-acetylglycerophosphocholine esterase) (2-acetyl-1-alkylglycerophosphocholine esterase) (Group-VIIA phospholipase A2) (gVIIA-PLA2) (LDL-associated phospholipase A2) (LDL-PLA(2)) (PAF 2-acylhydrolase)
PTAFR	Platelet-activating factor receptor (PAF-R) (PAFr)
PTGS2	Prostaglandin G/H synthase 2 (EC 1.14.99.1) (Cyclooxygenase-2) (COX-2) (PHS II) (Prostaglandin H2 synthase 2) (PGH synthase 2) (PGHS-2) (Prostaglandin-endoperoxide synthase 2)

PTX3	Pituitary homeobox 3 (Homeobox protein PITX3) (Paired-like homeodomain transcription factor 3)
S100A12	Protein S100-A12 (CGRP) (Calcium-binding protein in amniotic fluid 1) (CAAF1) (Calgranulin-C) (CAGC) (Extracellular newly identified RAGE-binding protein) (EN-RAGE) (Migration inhibitory factor-related protein 6) (MRP-6) (p6) (Neutrophil S100 protein) (S100 calcium-binding protein A12) [Cleaved into: Calcitermin]
S100A9	Protein S100-A9 (Calgranulin-B) (Calprotectin L1H subunit) (Leukocyte L1 complex heavy chain) (Migration inhibitory factor-related protein 14) (MRP-14) (p14) (S100 calcium-binding protein A9)
SAA1	Serum amyloid A-1 protein (SAA) [Cleaved into: Amyloid protein A (Amyloid fibril protein AA); Serum amyloid protein A(2-104); Serum amyloid protein A(3-104); Serum amyloid protein A(2-103); Serum amyloid protein A(2-102); Serum amyloid protein A(4-101)]
TIRAP	Toll/interleukin-1 receptor domain-containing adapter protein (TIR domain-containing adapter protein) (Adaptor protein Wyatt) (MyD88 adapter-like protein) (MyD88-2)
TLR1	Toll-like receptor 1 (EC 3.2.2.6) (Toll/interleukin-1 receptor-like protein) (TIL) (CD antigen CD281)
TLR2	Toll-like receptor 2 (EC 3.2.2.6) (Toll/interleukin-1 receptor-like protein 4) (CD antigen CD282)
TLR3	Toll-like receptor 3 (CD antigen CD283)
TPST1	Protein-tyrosine sulfotransferase 1 (EC 2.8.2.20) (Tyrosylprotein sulfotransferase 1) (TPST-1)
ABCA1	Phospholipid-transporting ATPase ABCA1 (EC 7.6.2.1) (ATP-binding cassette sub-family A member 1) (ATP-binding cassette transporter 1) (ABC-1) (ATP-binding cassette 1) (Cholesterol efflux regulatory protein)
ABI1	Abl interactor 1 (Abelson interactor 1) (Abi-1) (Abl-binding protein 4) (AblBP4) (Eps8 SH3 domain-binding protein) (Eps8-binding protein) (Nap1-binding protein) (Nap1BP) (Spectrin SH3 domain-binding protein 1) (e3B1)
ACVR1B	Activin receptor type-1B (EC 2.7.11.30) (Activin receptor type IB) (ACTR-IB) (Activin receptor-like kinase 4) (ALK-4) (Serine/threonine-protein kinase receptor R2) (SKR2)
ACVR2A	Activin receptor type-2A (EC 2.7.11.30) (Activin receptor type IIA) (ACTR-IIA) (ACTRIIA)
ADGRE1	Adhesion G protein-coupled receptor E1 (EGF-like module receptor 1) (EGF-like module-containing mucin-like hormone receptor-like 1) (EMR1 hormone receptor)
ADM	Pro-adrenomedullin [Cleaved into: Adrenomedullin (AM); Proadrenomedullin N-20 terminal peptide (ProAM N-terminal 20 peptide) (PAMP) (ProAM-N20)]
ADORA2B	Adenosine receptor A2b

ADRM1	Proteasomal ubiquitin receptor ADRM1 (110 kDa cell membrane glycoprotein) (Gp110) (Adhesion-regulating molecule 1) (ARM-1) (Proteasome regulatory particle non-ATPase 13) (hRpn13) (Rpn13 homolog)
AHR	Aryl hydrocarbon receptor (Ah receptor) (AhR) (Class E basic helix-loop-helix protein 76) (bHLHe76)
APLNR	Apelin receptor (Angiotensin receptor-like 1) (G-protein coupled receptor APJ) (G-protein coupled receptor HG11)
AQP9	Aquaporin-7 (AQP-7) (Aquaglyceroporin-7) (Aquaporin adipose) (AQPap) (Aquaporin-7-like)
ATP2A2	Sarcoplasmic/endoplasmic reticulum calcium ATPase 2 (SERCA2) (SR Ca(2+)-ATPase 2) (EC 7.2.2.10) (Calcium pump 2) (Calcium-transporting ATPase sarcoplasmic reticulum type, slow twitch skeletal muscle isoform) (Endoplasmic reticulum class 1/2 Ca(2+) ATPase)
ATP2B1	Plasma membrane calcium-transporting ATPase 1 (EC 7.2.2.10) (Plasma membrane calcium ATPase isoform 1) (PMCA1) (Plasma membrane calcium pump isoform 1)
ATP2C1	Calcium-transporting ATPase type 2C member 1 (ATPase 2C1) (EC 7.2.2.10) (ATP-dependent Ca(2+) pump PMR1) (Ca(2+)/Mn(2+)-ATPase 2C1) (Secretory pathway Ca(2+)-transporting ATPase type 1) (SPCA1)
AXL	Tyrosine-protein kinase receptor UFO (EC 2.7.10.1) (AXL oncogene)
BDKRB1	B1 bradykinin receptor (B1R) (BK-1 receptor)
BDKRB2,BDKRB1	B1 bradykinin receptor
BEST1	Bestrophin-1 (TU15B) (Vitelliform macular dystrophy protein 2)
BST2	Bone marrow stromal antigen 2 (BST-2) (HM1.24 antigen) (Tetherin) (CD antigen CD317)
BTG2	Protein BTG2 (BTG family member 2) (NGF-inducible anti-proliferative protein PC3)
C5AR1	C5a anaphylatoxin chemotactic receptor 1 (C5a anaphylatoxin chemotactic receptor) (C5a-R) (C5aR) (CD antigen CD88)
CALCRL	Calcitonin gene-related peptide type 1 receptor (CGRP type 1 receptor) (Calcitonin receptor-like receptor)
CCL22	C-C motif chemokine 22 (CC chemokine STCP-1) (MDC(1-69)) (Macrophage-derived chemokine) (Small-inducible cytokine A22) (Stimulated T-cell chemotactic protein 1) [Cleaved into: MDC(3-69); MDC(5-69); MDC(7-69)]
CCL24	C-C motif chemokine 24 (CK-beta-6) (Eosinophil chemotactic protein 2) (Eotaxin-2) (Myeloid progenitor inhibitory factor 2) (MPIF-2) (Small-inducible cytokine A24)
CCL5	C-C motif chemokine 5 (EoCP) (Eosinophil chemotactic cytokine) (SIS-delta) (Small-inducible cytokine A5) (T cell-specific protein P228) (TCP228) (T-cell-specific protein RANTES) [Cleaved into: RANTES(3-68); RANTES(4-68)]

CCR7	C-C chemokine receptor type 7 (C-C CKR-7) (CC-CKR-7) (CCR-7) (BLR2) (CDw197) (Epstein-Barr virus-induced G-protein coupled receptor 1) (EBI1) (EBV-induced G-protein coupled receptor 1) (MIP-3 beta receptor) (CD antigen CD197)
CCRL2	C-C chemokine receptor-like 2 (Chemokine receptor CCR11) (Chemokine receptor X) (Putative MCP-1 chemokine receptor)
CD48	CD48 antigen (B-lymphocyte activation marker BLAST-1) (BCM1 surface antigen) (Leukocyte antigen MEM-102) (SLAM family member 2) (SLAMF2) (Signaling lymphocytic activation molecule 2) (TCT.1) (CD antigen CD48)
CD55	Complement decay-accelerating factor (CD antigen CD55)
CD69	Early activation antigen CD69 (Activation inducer molecule) (AIM) (BL-AC/P26) (C-type lectin domain family 2 member C) (EA1) (Early T-cell activation antigen p60) (GP32/28) (Leukocyte surface antigen Leu-23) (MLR-3) (CD antigen CD69)
CD70	CD70 antigen (CD27 ligand) (CD27-L) (Tumor necrosis factor ligand superfamily member 7) (CD antigen CD70)
CD82	CD82 antigen (C33 antigen) (IA4) (Inducible membrane protein R2) (Metastasis suppressor Kangai-1) (Suppressor of tumorigenicity 6 protein) (Tetraspanin-27) (Tspan-27) (CD antigen CD82)
CDKN1A	Cyclin-dependent kinase inhibitor 1 (CDK-interacting protein 1) (Melanoma differentiation-associated protein 6) (MDA-6) (p21)
CHST2	Carbohydrate sulfotransferase 2 (EC 2.8.2.-) (Galactose/N-acetylglucosamine/N-acetylglucosamine 6-O-sulfotransferase 2) (GST-2) (N-acetylglucosamine 6-O-sulfotransferase 1) (GlcNAc6ST-1) (Gn6ST-1)
CLEC5A	C-type lectin domain family 5 member A (C-type lectin superfamily member 5) (Myeloid DAP12-associating lectin 1) (MDL-1)
CMKLR1	Chemokine-like receptor 1 (G-protein coupled receptor ChemR23) (G-protein coupled receptor DEZ)
CSF1	Macrophage colony-stimulating factor 1 (CSF-1) (M-CSF) (MCSF) (Lanimostim) [Cleaved into: Processed macrophage colony-stimulating factor 1]
CSF3	Granulocyte colony-stimulating factor (G-CSF) (Pluripoietin) (Filgrastim) (Lenograstim)
CSF3R	Granulocyte colony-stimulating factor receptor (G-CSF receptor) (G-CSF-R) (CD antigen CD114)
CXCR6	C-X-C chemokine receptor type 6 (CXC-R6) (CXCR-6) (CDw186) (G-protein coupled receptor STRL33) (G-protein coupled receptor bonzo) (CD antigen CD186)
DCBLD2	Discoidin, CUB and LCCL domain-containing protein 2 (CUB, LCCL and coagulation factor V/VIII-homology domains protein 1) (Endothelial and smooth muscle cell-derived neuropilin-like protein)
EBI3	Interleukin-27 subunit beta (IL-27 subunit beta) (IL-27B) (Epstein-Barr virus-induced gene 3 protein) (EBV-induced gene 3 protein)

EDN1	Endothelin-1 (Preproendothelin-1) (PPET1) [Cleaved into: Endothelin-1 (ET-1); Big endothelin-1]
EIF2AK2	Interferon-induced, double-stranded RNA-activated protein kinase (EC 2.7.11.1) (Eukaryotic translation initiation factor 2-alpha kinase 2) (eIF-2A protein kinase 2) (Interferon-inducible RNA-dependent protein kinase) (P1/eIF-2A protein kinase) (Protein kinase RNA-activated) (PKR) (Protein kinase R) (Tyrosine-protein kinase EIF2AK2) (EC 2.7.10.2) (p68 kinase)
EMP3	Epithelial membrane protein 3 (EMP-3) (Hematopoietic neural membrane protein 1) (HNMP-1) (Protein YMP)
EREG	Proepiregulin [Cleaved into: Epiregulin (EPR)]
F3	Tissue factor (TF) (Coagulation factor III) (Thromboplastin) (CD antigen CD142)
FFAR2	Free fatty acid receptor 2 (G-protein coupled receptor 43)
FZD5	Frizzled-5 (Fz-5) (hFz5) (FzE5)
GABBR1	Gamma-aminobutyric acid type B receptor subunit 1 (GABA-B receptor 1) (GABA-B-R1) (GABA-BR1) (GABABR1) (Gb1)
GCH1	GTP cyclohydrolase 1 (EC 3.5.4.16) (GTP cyclohydrolase I) (GTP-CH-I)
GNA15	Guanine nucleotide-binding protein subunit alpha-15 (G alpha-15) (G-protein subunit alpha-15) (Epididymis tissue protein Li 17E) (Guanine nucleotide-binding protein subunit alpha-16) (G alpha-16) (G-protein subunit alpha-16)
GNAI3	Guanine nucleotide-binding protein G(i) subunit alpha-3 (G(i) alpha-3)
GP1BA	Platelet glycoprotein Ib alpha chain (GP-Ib alpha) (GPIb-alpha) (GPIbA) (Glycoprotein Ibalpha) (Antigen CD42b-alpha) (CD antigen CD42b) [Cleaved into: Glycocalicin]
GPC3	Glypican-3 (GTR2-2) (Intestinal protein OCI-5) (MXR7) [Cleaved into: Glypican-3 alpha subunit; Glypican-3 beta subunit]
GPR132	Probable G-protein coupled receptor 132 (G2 accumulation protein)
GPR183	G-protein coupled receptor 183 (Epstein-Barr virus-induced G-protein coupled receptor 2) (EBI2) (EBV-induced G-protein coupled receptor 2) (hEBI2)
HAS2	Hyaluronan synthase 2 (EC 2.4.1.212) (Hyaluronate synthase 2) (Hyaluronic acid synthase 2) (HA synthase 2)
HBEGF	Proheparin-binding EGF-like growth factor [Cleaved into: Heparin-binding EGF-like growth factor (HB-EGF) (HBEGF) (Diphtheria toxin receptor) (DT-R)]
HIF1A	Hypoxia-inducible factor 1-alpha (HIF-1-alpha) (HIF1-alpha) (ARNT-interacting protein) (Basic-helix-loop-helix-PAS protein MOP1) (Class E basic helix-loop-helix protein 78) (bHLHe78) (Member of PAS protein 1) (PAS domain-containing protein 8)
HPN	Serine protease hepsin (EC 3.4.21.106) (Transmembrane protease serine 1) [Cleaved into: Serine protease hepsin non-catalytic chain;

	Serine protease hepsin catalytic chain]
HRH1	Histamine H1 receptor (H1R) (HH1R)
ICAM1	Intercellular adhesion molecule 1 (ICAM-1) (Major group rhinovirus receptor) (CD antigen CD54)
ICAM4	Intercellular adhesion molecule 4 (ICAM-4) (Landsteiner-Wiener blood group glycoprotein) (LW blood group protein) (CD antigen CD242)
ICOSLG	ICOS ligand (B7 homolog 2) (B7-H2) (B7-like protein Gl50) (B7-related protein 1) (B7RP-1) (CD antigen CD275)
IFITM1	Interferon-induced transmembrane protein 1 (Dispanin subfamily A member 2a) (DSPA2a) (Interferon-induced protein 17) (Interferon-inducible protein 9-27) (Leu-13 antigen) (CD antigen CD225)
IFNAR1	Interferon alpha/beta receptor 1 (IFN-R-1) (IFN-alpha/beta receptor 1) (Cytokine receptor class-II member 1) (Cytokine receptor family 2 member 1) (CRF2-1) (Type I interferon receptor 1)
IFNGR2	Interferon gamma receptor 2 (IFN-gamma receptor 2) (IFN-gamma-R2) (Interferon gamma receptor accessory factor 1) (AF-1) (Interferon gamma receptor beta-chain) (IFN-gamma-R-beta) (Interferon gamma transducer 1)
IL10	Interleukin-10 (IL-10) (Cytokine synthesis inhibitory factor) (CSIF)
IL10RA	Interleukin-10 receptor subunit alpha (IL-10 receptor subunit alpha) (IL-10R subunit alpha) (IL-10RA) (CDw210a) (Interleukin-10 receptor subunit 1) (IL-10R subunit 1) (IL-10R1) (CD antigen CD210)
IL12B	Interleukin-12 subunit beta (IL-12B) (Cytotoxic lymphocyte maturation factor 40 kDa subunit) (CLMF p40) (IL-12 subunit p40) (NK cell stimulatory factor chain 2) (NKSF2)
IL15	Interleukin-15 (IL-15)
IL15RA	Interleukin-15 receptor subunit alpha (IL-15 receptor subunit alpha) (IL-15R-alpha) (IL-15RA) (CD antigen CD215) [Cleaved into: Soluble interleukin-15 receptor subunit alpha (sIL-15 receptor subunit alpha) (sIL-15R-alpha) (sIL-15RA)]
IL18	Interleukin-18 (IL-18) (Iboctadekin) (Interferon gamma-inducing factor) (IFN-gamma-inducing factor) (Interleukin-1 gamma) (IL-1 gamma)
IL18R1	Interleukin-18 receptor 1 (IL-18R-1) (IL-18R1) (EC 3.2.2.6) (CD218 antigen-like family member A) (CDw218a) (IL1 receptor-related protein) (IL-1Rrp) (IL1R-rp) (Interleukin-18 receptor alpha) (IL-18R-alpha) (IL-18Ralpha) (CD antigen CD218a)
IL18RAP	Interleukin-18 receptor accessory protein (IL-18 receptor accessory protein) (IL-18RAcP) (EC 3.2.2.6) (Accessory protein-like) (AcPL) (CD218 antigen-like family member B) (CDw218b) (IL-1R accessory protein-like) (IL-1RAcPL) (Interleukin-1 receptor 7) (IL-1R-7) (IL-

	1R7) (Interleukin-18 receptor accessory protein-like) (Interleukin-18 receptor beta) (IL-18R-beta) (IL-18Rbeta) (CD antigen CD218b)
IL6	Interleukin-6 (IL-6) (B-cell stimulatory factor 2) (BSF-2) (CTL differentiation factor) (CDF) (Hybridoma growth factor) (Interferon beta-2) (IFN-beta-2)
IL7R	Interleukin-7 receptor subunit alpha (IL-7 receptor subunit alpha) (IL-7R subunit alpha) (IL-7R-alpha) (IL-7RA) (CDw127) (CD antigen CD127)
INHBA	Inhibin beta A chain (Activin beta-A chain) (Erythroid differentiation protein) (EDF)
IRAK2	Interleukin-1 receptor-associated kinase-like 2 (IRAK-2)
IRF1	Interferon regulatory factor 1 (IRF-1)
IRF7	Interferon regulatory factor 7 (IRF-7)
ITGA5	Integrin alpha-5 (CD49 antigen-like family member E) (Fibronectin receptor subunit alpha) (Integrin alpha-F) (VLA-5) (CD antigen CD49e) [Cleaved into: Integrin alpha-5 heavy chain; Integrin alpha-5 light chain]
ITGB3	Integrin beta-3 (Platelet membrane glycoprotein IIIa) (GPIIIa) (CD antigen CD61)
ITGB8	Integrin beta-8
KCNA3	Potassium voltage-gated channel subfamily A member 3 (HGK5) (HLK3) (HPCN3) (Voltage-gated K(+) channel HuKIII) (Voltage-gated potassium channel subunit Kv1.3)
KCNJ2	Inward rectifier potassium channel 2 (Cardiac inward rectifier potassium channel) (Inward rectifier K(+) channel Kir2.1) (IRK-1) (hIRK1) (Potassium channel, inwardly rectifying subfamily J member 2)
KCNMB2	Calcium-activated potassium channel subunit beta-3 (BK channel subunit beta-3) (BKbeta3) (Hbeta3) (Calcium-activated potassium channel, subfamily M subunit beta-3) (Charybdotoxin receptor subunit beta-3) (K(VCA)beta-3) (Maxi K channel subunit beta-3) (Slo-beta-3)
KIF1B	Kinesin-like protein KIF1B (Klp)
KLF6	Krueppel-like factor 6 (B-cell-derived protein 1) (Core promoter element-binding protein) (GC-rich sites-binding factor GBF) (Proto-oncogene BCD1) (Suppressor of tumorigenicity 12 protein) (Transcription factor Zf9)
LAMP3	Lysosome-associated membrane glycoprotein 3 (LAMP-3) (Lysosomal-associated membrane protein 3) (DC-lysosome-associated membrane glycoprotein) (DC LAMP) (Protein TSC403) (CD antigen CD208)
LCP2	Lymphocyte cytosolic protein 2 (SH2 domain-containing leukocyte protein of 76 kDa) (SLP-76 tyrosine phosphoprotein) (SLP76)
LDLR	Low-density lipoprotein receptor (LDL receptor)

LIF	Leukemia inhibitory factor (LIF) (Differentiation-stimulating factor) (D factor) (Melanoma-derived LPL inhibitor) (MLPLI) (Emfilermin)
LPAR1	Lysophosphatidic acid receptor 1 (LPA receptor 1) (LPA-1) (Lysophosphatidic acid receptor Edg-2)
LTA	Lymphotoxin-alpha (LT-alpha) (TNF-beta) (Tumor necrosis factor ligand superfamily member 1)
LY6E	Lymphocyte antigen 6E (Ly-6E) (Retinoic acid-induced gene E protein) (RIG-E) (Stem cell antigen 2) (SCA-2) (Thymic shared antigen 1) (TSA-1)
LYN	Tyrosine-protein kinase Lyn (EC 2.7.10.2) (Lck/Yes-related novel protein tyrosine kinase) (V-yes-1 Yamaguchi sarcoma viral related oncogene homolog) (p53Lyn) (p56Lyn)
MARCO	Macrophage receptor MARCO (Macrophage receptor with collagenous structure) (Scavenger receptor class A member 2)
MEFV	Pyrin (Marenostrin)
MEP1A	Meprin A subunit alpha (EC 3.4.24.18) (Endopeptidase-2) (N-benzoyl-L-tyrosyl-P-amino-benzoic acid hydrolase subunit alpha) (PABA peptide hydrolase) (PPH alpha)
MET	Hepatocyte growth factor receptor (HGF receptor) (EC 2.7.10.1) (HGF/SF receptor) (Proto-oncogene c-Met) (Scatter factor receptor) (SF receptor) (Tyrosine-protein kinase Met)
MMP14	Matrix metalloproteinase-14 (MMP-14) (EC 3.4.24.80) (MMP-X1) (Membrane-type matrix metalloproteinase 1) (MT-MMP 1) (MTMMP1) (Membrane-type-1 matrix metalloproteinase) (MT1-MMP) (MT1MMP)
MSR1	Macrophage scavenger receptor types I and II (Macrophage acetylated LDL receptor I and II) (Scavenger receptor class A member 1) (CD antigen CD204)
MXD1	Max dimerization protein 1 (Max dimerizer 1) (Protein MAD)
MYC	Myc proto-oncogene protein (Class E basic helix-loop-helix protein 39) (bHLHe39) (Proto-oncogene c-Myc) (Transcription factor p64)
NAMPT	Nicotinamide phosphoribosyltransferase (NAmPRTase) (Nampt) (EC 2.4.2.12) (Pre-B-cell colony-enhancing factor 1) (Pre-B cell-enhancing factor) (Visfatin)
NDP	Norrin (Norrie disease protein) (X-linked exudative vitreoretinopathy 2 protein)
NFKBIA	NF-kappa-B inhibitor alpha (I-kappa-B-alpha) (IkB-alpha) (IkappaBalpha) (Major histocompatibility complex enhancer-binding protein MAD3)
NLRP3	NACHT, LRR and PYD domains-containing protein 3 (Angiotensin/vasopressin receptor AII/AVP-like) (Caterpiller protein 1.1) (CLR1.1) (Cold-induced autoinflammatory syndrome 1 protein) (Cryopyrin) (PYRIN-containing APAF1-like protein 1)
NMUR1	Neuromedin-U receptor 1 (NMU-R1) (G-protein coupled receptor 66) (G-protein coupled receptor FM-3)

NOD2	Nucleotide-binding oligomerization domain-containing protein 2 (Caspase recruitment domain-containing protein 15) (Inflammatory bowel disease protein 1)
NPFFR2	Neuropeptide FF receptor 2 (G-protein coupled receptor 74) (G-protein coupled receptor HLWAR77) (Neuropeptide G-protein coupled receptor)
OLR1	Oxidized low-density lipoprotein receptor 1 (Ox-LDL receptor 1) (C-type lectin domain family 8 member A) (Lectin-like oxidized LDL receptor 1) (LOX-1) (Lectin-like oxLDL receptor 1) (hLOX-1) (Lectin-type oxidized LDL receptor 1) [Cleaved into: Oxidized low-density lipoprotein receptor 1, soluble form]
OPRK1	Kappa-type opioid receptor (K-OR-1) (KOR-1)
OSM	Oncostatin-M (OSM)
OSMR	Oncostatin-M-specific receptor subunit beta (Interleukin-31 receptor subunit beta) (IL-31 receptor subunit beta) (IL-31R subunit beta) (IL-31R-beta) (IL-31RB)
P2RX4	P2X purinoceptor 4 (P2X4) (ATP receptor) (Purinergic receptor)
P2RX7	P2X purinoceptor 7 (P2X7) (ATP receptor) (P2Z receptor) (Purinergic receptor)
P2RY2	P2Y purinoceptor 2 (P2Y2) (ATP receptor) (P2U purinoceptor 1) (P2U1) (P2U receptor 1) (Purinergic receptor)
PCDH7	Protocadherin-7 (Brain-heart protocadherin) (BH-Pcdh)
PDE4B	cAMP-specific 3',5'-cyclic phosphodiesterase 4B (EC 3.1.4.53) (DPDE4) (PDE32)
PDPN	Podoplanin (Aggrus) (Glycoprotein 36) (Gp36) (PA2.26 antigen) (T1-alpha) (T1A) [Cleaved into: 29kDa cytosolic podoplanin intracellular domain (PICD)]
PIK3R5	Phosphoinositide 3-kinase regulatory subunit 5 (PI3-kinase regulatory subunit 5) (PI3-kinase p101 subunit) (Phosphatidylinositol 4,5-bisphosphate 3-kinase regulatory subunit) (PtdIns-3-kinase regulatory subunit) (Protein FOAP-2) (PtdIns-3-kinase p101) (p101-PI3K)
PLAUR	Urokinase plasminogen activator surface receptor (U-PAR) (uPAR) (Monocyte activation antigen Mo3) (CD antigen CD87)
PROK2	Prokineticin-2 (PK2) (Protein Bv8 homolog)
PSEN1	Presenilin-1 (PS-1) (EC 3.4.23.-) (Protein S182) [Cleaved into: Presenilin-1 NTF subunit; Presenilin-1 CTF subunit; Presenilin-1 CTF12 (PS1-CTF12)]
PTGER2	Prostaglandin E2 receptor EP2 subtype (PGE receptor EP2 subtype) (PGE2 receptor EP2 subtype) (Prostanoid EP2 receptor)
PTGER2,PTGER4	Prostaglandin E2 receptor EP4 subtype (PGE receptor EP4 subtype) (PGE2 receptor EP4 subtype) (Prostanoid EP4 receptor)
PTGER4	Prostaglandin E2 receptor EP4 subtype (Prostanoid EP4 receptor) (Fragment)
PTGIR	Prostacyclin receptor (Prostaglandin I2 receptor) (PGI receptor) (PGI2 receptor) (Prostanoid IP receptor)

PTPRE	Receptor-type tyrosine-protein phosphatase epsilon (Protein-tyrosine phosphatase epsilon) (R-PTP-epsilon) (EC 3.1.3.48)
PVR	Poliovirus receptor (Nectin-like protein 5) (NECL-5) (CD antigen CD155)
RAF1	RAF proto-oncogene serine/threonine-protein kinase (EC 2.7.11.1) (Proto-oncogene c-RAF) (cRaf) (Raf-1)
RASGRP1	RAS guanyl-releasing protein 1 (Calcium and DAG-regulated guanine nucleotide exchange factor II) (CalDAG-GEFII) (Ras guanyl-releasing protein)
RELA	Transcription factor p65 (Nuclear factor NF-kappa-B p65 subunit) (Nuclear factor of kappa light polypeptide gene enhancer in B-cells 3)
RGS1	Regulator of G-protein signaling 1 (RGS1) (B-cell activation protein BL34) (Early response protein 1R20)
RGS16	Regulator of G-protein signaling 16 (RGS16) (A28-RGS14P) (Retinal-specific RGS) (RGS-r) (hRGS-r) (Retinally abundant regulator of G-protein signaling)
RHOG	Rho-related GTP-binding protein RhoG
RIPK2	Receptor-interacting serine/threonine-protein kinase 2 (EC 2.7.11.1) (CARD-containing interleukin-1 beta-converting enzyme-associated kinase) (CARD-containing IL-1 beta ICE-kinase) (RIP-like-interacting CLARP kinase) (Receptor-interacting protein 2) (RIP-2) (Tyrosine-protein kinase RIPK2) (EC 2.7.10.2)
RNF144B	E3 ubiquitin-protein ligase RNF144B (EC 2.3.2.31) (IBR domain-containing protein 2) (RING finger protein 144B) (p53-inducible RING finger protein)
ROS1	Proto-oncogene tyrosine-protein kinase ROS (EC 2.7.10.1) (Proto-oncogene c-Ros) (Proto-oncogene c-Ros-1) (Receptor tyrosine kinase c-ros oncogene 1) (c-Ros receptor tyrosine kinase)
RTP4	Receptor-transporting protein 4 (28 kDa interferon-responsive protein) (3CxxC-type zinc finger protein 4)
SCARF1	Scavenger receptor class F member 1 (Acetyl LDL receptor) (Scavenger receptor expressed by endothelial cells 1) (SREC-I)
SCN1B	Sodium channel subunit beta-1
SELE	E-selectin (CD62 antigen-like family member E) (Endothelial leukocyte adhesion molecule 1) (ELAM-1) (Leukocyte-endothelial cell adhesion molecule 2) (LECAM2) (CD antigen CD62E)
SELENOS	Selenoprotein S (SelS) (VCP-interacting membrane protein)
SELL	L-selectin (CD62 antigen-like family member L) (Leukocyte adhesion molecule 1) (LAM-1) (Leukocyte surface antigen Leu-8) (Leukocyte-endothelial cell adhesion molecule 1) (LECAM1) (Lymph node homing receptor) (TQ1) (gp90-MEL) (CD antigen CD62L)
SEMA4D	Semaphorin-4D (A8) (BB18) (GR3) (CD antigen CD100)
SERPINE1	Plasminogen activator inhibitor 1 (PAI) (PAI-1) (Endothelial plasminogen activator inhibitor) (Serpin E1)

SGMS2	Phosphatidylcholine:ceramide cholinephosphotransferase 2 (EC 2.7.8.27) (Sphingomyelin synthase 2)
SLAMF1	Signaling lymphocytic activation molecule (CDw150) (IPO-3) (SLAM family member 1) (CD antigen CD150)
SLC11A2	Natural resistance-associated macrophage protein 2 (NRAMP 2) (Divalent cation transporter 1) (Divalent metal transporter 1) (DMT-1) (Solute carrier family 11 member 2)
SLC1A2	Excitatory amino acid transporter 2 (Glutamate/aspartate transporter II) (Sodium-dependent glutamate/aspartate transporter 2) (Solute carrier family 1 member 2)
SLC28A2	Sodium/nucleoside cotransporter 2 (Concentrative nucleoside transporter 2) (CNT 2) (hCNT2) (Na(+)/nucleoside cotransporter 2) (Sodium-coupled nucleoside transporter 2) (Sodium/purine nucleoside co-transporter) (SPNT) (Solute carrier family 28 member 2)
SLC31A1	High affinity copper uptake protein 1 (Copper transporter 1) (hCTR1) (Solute carrier family 31 member 1)
SLC31A2	Probable low affinity copper uptake protein 2 (Copper transporter 2) (hCTR2) (Solute carrier family 31 member 2)
SLC4A4	Electrogenic sodium bicarbonate cotransporter 1 (Sodium bicarbonate cotransporter) (Na(+)/HCO3(-) cotransporter) (Solute carrier family 4 member 4) (kNBC1)
SLC7A1	High affinity cationic amino acid transporter 1 (CAT-1) (CAT1) (Ecotropic retroviral leukemia receptor homolog) (Ecotropic retrovirus receptor homolog) (Solute carrier family 7 member 1) (System Y+ basic amino acid transporter)
SLC7A2	Cationic amino acid transporter 2 (CAT-2) (CAT2) (Low affinity cationic amino acid transporter 2) (Solute carrier family 7 member 2)
SPHK1	Sphingosine kinase 1 (SK 1) (SPK 1) (EC 2.7.1.91) (Acetyltransferase SPHK1) (EC 2.3.1.-)
SRI	Sorcin (22 kDa protein) (CP-22) (CP22) (V19)
STAB1	Stabilin-1 (Fasciclin, EGF-like, laminin-type EGF-like and link domain-containing scavenger receptor 1) (FEEL-1) (MS-1 antigen)
TACR1	Substance-P receptor (SPR) (NK-1 receptor) (NK-1R) (Tachykinin receptor 1)
TACR3	Neuromedin-K receptor (NKR) (NK-3 receptor) (NK-3R) (Neurokinin B receptor) (Tachykinin receptor 3)
TAPBP	Tapasin (TPN) (TPSN) (NGS-17) (TAP-associated protein) (TAP-binding protein)
TIMP1	Metalloproteinase inhibitor 1 (Erythroid-potentiating activity) (EPA) (Fibroblast collagenase inhibitor) (Collagenase inhibitor) (Tissue inhibitor of metalloproteinases 1) (TIMP-1)
TNFAIP6	Tumor necrosis factor-inducible gene 6 protein (Hyaluronate-binding protein) (TNF-stimulated gene 6 protein) (TSG-6) (Tumor necrosis factor alpha-induced protein 6) (TNF alpha-induced

	protein 6)
TNFRSF9	Tumor necrosis factor receptor superfamily member 9 (4-1BB ligand receptor) (CDw137) (T-cell antigen 4-1BB homolog) (T-cell antigen ILA) (CD antigen CD137)
TNFSF10	Tumor necrosis factor ligand superfamily member 10 (Apo-2 ligand) (Apo-2L) (TNF-related apoptosis-inducing ligand) (Protein TRAIL) (CD antigen CD253)
TNFSF15	Tumor necrosis factor ligand superfamily member 15 (TNF ligand-related molecule 1) (Vascular endothelial cell growth inhibitor) [Cleaved into: Tumor necrosis factor ligand superfamily member 15, membrane form; Tumor necrosis factor ligand superfamily member 15, secreted form]
TNFSF9	Tumor necrosis factor ligand superfamily member 9 (4-1BB ligand) (4-1BBL)
TPBG	Trophoblast glycoprotein (5T4 oncofetal antigen) (5T4 oncofetal trophoblast glycoprotein) (5T4 oncotrophoblast glycoprotein) (M6P1) (Wnt-activated inhibitory factor 1) (WAIF1)
VIP	VIP peptides [Cleaved into: Intestinal peptide PHV-42 (Peptide histidine valine 42); Intestinal peptide PHM-27 (Peptide histidine methioninamide 27); Vasoactive intestinal peptide (VIP) (Vasoactive intestinal polypeptide)]
ABCC1	Multidrug resistance-associated protein 1 (EC 7.6.2.2) (ATP-binding cassette sub-family C member 1) (Glutathione-S-conjugate-translocating ATPase ABCC1) (EC 7.6.2.3) (Leukotriene C(4) transporter) (LTC4 transporter)
ABHD12	Lysophosphatidylserine lipase ABHD12 (EC 3.1.-.-) (2-arachidonoylglycerol hydrolase ABHD12) (Abhydrolase domain-containing protein 12) (hABHD12) (Monoacylglycerol lipase ABHD12) (EC 3.1.1.23) (Oxidized phosphatidylserine lipase ABHD12) (EC 3.1.-.-)
ACE2	Angiotensin-converting enzyme 2 (EC 3.4.17.23) (Angiotensin-converting enzyme homolog) (ACEH) (Angiotensin-converting enzyme-related carboxypeptidase) (ACE-related carboxypeptidase) (EC 3.4.17.-) (Metalloprotease MPROT15) [Cleaved into: Processed angiotensin-converting enzyme 2]
ACOD1	Cis-aconitate decarboxylase (CAD) (EC 4.1.1.6) (Aconitate decarboxylase) (Aconitate decarboxylase 1) (Cis-aconitic acid decarboxylase) (Immune-responsive gene 1 protein)
ADA	Adenosine deaminase (EC 3.5.4.4) (Adenosine aminohydrolase)
ADAM8	Disintegrin and metalloproteinase domain-containing protein 8 (ADAM 8) (EC 3.4.24.-) (Cell surface antigen MS2) (CD antigen CD156a)
ADAMTS12	A disintegrin and metalloproteinase with thrombospondin motifs 12 (ADAM-TS 12) (ADAM-TS12) (ADAMTS-12) (EC 3.4.24.-)

ADCYAP1	Pituitary adenylate cyclase-activating polypeptide (PACAP) [Cleaved into: PACAP-related peptide (PRP-48); Pituitary adenylate cyclase-activating polypeptide 27 (PACAP-27) (PACAP27); Pituitary adenylate cyclase-activating polypeptide 38 (PACAP-38) (PACAP38)]
ADIPOQ	Adiponectin (30 kDa adipocyte complement-related protein) (Adipocyte complement-related 30 kDa protein) (ACRP30) (Adipocyte, C1q and collagen domain-containing protein) (Adipose most abundant gene transcript 1 protein) (apM-1) (Gelatin-binding protein)
AGT	Angiotensinogen (Serpin A8) [Cleaved into: Angiotensin-1 (Angiotensin 1-10) (Angiotensin I) (Ang I); Angiotensin-2 (Angiotensin 1-8) (Angiotensin II) (Ang II); Angiotensin-3 (Angiotensin 2-8) (Angiotensin III) (Ang III) (Des-Asp[1]-angiotensin II); Angiotensin-4 (Angiotensin 3-8) (Angiotensin IV) (Ang IV); Angiotensin 1-9; Angiotensin 1-7; Angiotensin 1-5; Angiotensin 1-4]
AGTR1	Type-1 angiotensin II receptor (AT1AR) (AT1BR) (Angiotensin II type-1 receptor) (AT1)
AHSG	Alpha-2-HS-glycoprotein (Alpha-2-Z-globulin) (Ba-alpha-2-glycoprotein) (Fetuin-A) [Cleaved into: Alpha-2-HS-glycoprotein chain A; Alpha-2-HS-glycoprotein chain B]
AKNA	Microtubule organization protein AKNA (AT-hook-containing transcription factor)
APCS	Serum amyloid P-component (SAP) (9.5S alpha-1-glycoprotein) [Cleaved into: Serum amyloid P-component(1-203)]
APOA1	Apolipoprotein A-I (Apo-AI) (ApoA-I) (Apolipoprotein A1) [Cleaved into: Proapolipoprotein A-I (ProapoA-I); Truncated apolipoprotein A-I (Apolipoprotein A-I(1-242))]
APOE	Apolipoprotein E (Apo-E)
APP	Amyloid-beta precursor protein (APP) (ABPP) (APPI) (Alzheimer disease amyloid protein) (Amyloid precursor protein) (Amyloid-beta A4 protein) (Cerebral vascular amyloid peptide) (CVAP) (PreA4) (Protease nexin-II) (PN-II) [Cleaved into: N-APP; Soluble APP-alpha (S-APP-alpha); Soluble APP-beta (S-APP-beta); C99 (Beta-secretase C-terminal fragment) (Beta-CTF); Amyloid-beta protein 42 (Abeta42) (Beta-APP42); Amyloid-beta protein 40 (Abeta40) (Beta-APP40); C83 (Alpha-secretase C-terminal fragment) (Alpha-CTF); P3(42); P3(40); C80; Gamma-secretase C-terminal fragment 59 (Amyloid intracellular domain 59) (AICD-59) (AID(59)) (Gamma-CTF(59)); Gamma-secretase C-terminal fragment 57 (Amyloid intracellular domain 57) (AICD-57) (AID(57)) (Gamma-CTF(57)); Gamma-secretase C-terminal fragment 50 (Amyloid intracellular domain 50) (AICD-50) (AID(50)) (Gamma-CTF(50)); C31]

AREL1	Apoptosis-resistant E3 ubiquitin protein ligase 1 (EC 2.3.2.26) (Apoptosis-resistant HECT-type E3 ubiquitin transferase 1)
ASH1L	Histone-lysine N-methyltransferase ASH1L (EC 2.1.1.359) (EC 2.1.1.367) (ASH1-like protein) (huASH1) (Absent small and homeotic disks protein 1 homolog) (Lysine N-methyltransferase 2H)
ATM	Serine-protein kinase ATM (EC 2.7.11.1) (Ataxia telangiectasia mutated) (A-T mutated)
BAP1	Ubiquitin carboxyl-terminal hydrolase BAP1 (EC 3.4.19.12) (BRCA1-associated protein 1) (Cerebral protein 6)
BCL6B	B-cell CLL/lymphoma 6 member B protein (Bcl6-associated zinc finger protein) (Zinc finger protein 62)
BIRC2	Baculoviral IAP repeat-containing protein 2 (EC 2.3.2.27) (Cellular inhibitor of apoptosis 1) (C-IAP1) (IAP homolog B) (Inhibitor of apoptosis protein 2) (hIAP-2) (hIAP2) (RING finger protein 48) (RING-type E3 ubiquitin transferase BIRC2) (TNFR2-TRAF-signaling complex protein 2)
BIRC3	Baculoviral IAP repeat-containing protein 3 (EC 2.3.2.27) (Apoptosis inhibitor 2) (API2) (Cellular inhibitor of apoptosis 2) (C-IAP2) (IAP homolog C) (Inhibitor of apoptosis protein 1) (hIAP-1) (hIAP1) (RING finger protein 49) (RING-type E3 ubiquitin transferase BIRC3) (TNFR2-TRAF-signaling complex protein 1)
BRD4	Bromodomain-containing protein 4 (Protein HUNK1)
BST1	ADP-ribosyl cyclase/cyclic ADP-ribose hydrolase 2 (EC 3.2.2.6) (ADP-ribosyl cyclase 2) (Bone marrow stromal antigen 1) (BST-1) (Cyclic ADP-ribose hydrolase 2) (cADPr hydrolase 2) (CD antigen CD157)
BTK	Tyrosine-protein kinase BTK (EC 2.7.10.2) (Agammaglobulinemia tyrosine kinase) (ATK) (B-cell progenitor kinase) (BPK) (Bruton tyrosine kinase)
C1QTNF12	Adipolin (Adipose-derived insulin-sensitizing factor) (C1q and TNF related protein 12) (Complement C1q tumor necrosis factor-related protein 12) [Cleaved into: Adipolin fC1QTNF12 (Adipolin fCTRP12) (Adipolin full-length form); Adipolin gC1QTNF12 (Adipolin cleaved form) (Adipolin gCTRP12)]
C1QTNF3	Complement C1q tumor necrosis factor-related protein 3 (Collagenous repeat-containing sequence 26 kDa protein) (CORS26) (Secretory protein CORS26)
C2CD4A	C2 calcium-dependent domain-containing protein 4A (Nuclear-localized factor 1) (Protein FAM148A)
C2CD4B	C2 calcium-dependent domain-containing protein 4B (Nuclear-localized factor 2) (Protein FAM148B)
CASP1	Caspase-1 (CASP-1) (EC 3.4.22.36) (Interleukin-1 beta convertase) (IL-1BC) (Interleukin-1 beta-converting enzyme) (ICE) (IL-1 beta-converting enzyme) (p45) [Cleaved into: Caspase-1 subunit p20; Caspase-1 subunit p10]

CASP12	Inactive caspase-12 (CASP-12)
CASP4	Caspase-4 (CASP-4) (EC 3.4.22.57) (ICE and Ced-3 homolog 2) (ICH-2) (ICE(rel)-II) (Mih1) (Protease TX) [Cleaved into: Caspase-4 subunit p10; Caspase-4 subunit p20]
CASP5	Caspase-5 (CASP-5) (EC 3.4.22.58) (ICE(rel)-III) (Protease ICH-3) (Protease TY) [Cleaved into: Caspase-5 subunit p20; Caspase-5 subunit p10]
CCL1	C-C motif chemokine 1 (Small-inducible cytokine A1) (T lymphocyte-secreted protein I-309)
CCN3	CCN family member 3 (Cellular communication network factor 3) (Insulin-like growth factor-binding protein 9) (IBP-9) (IGF-binding protein 9) (IGFBP-9) (Nephro blastoma-overexpressed gene protein homolog) (Protein NOV homolog) (NovH)
CCN4	CCN family member 4 (WNT1-inducible-signaling pathway protein 1) (WISP-1) (Wnt-1-induced secreted protein)
CD200	OX-2 membrane glycoprotein (CD antigen CD200)
CD200R1	Cell surface glycoprotein CD200 receptor 1 (CD200 cell surface glycoprotein receptor) (Cell surface glycoprotein OX2 receptor 1)
CD200R1L	Cell surface glycoprotein CD200 receptor 2 (CD200 cell surface glycoprotein receptor-like 2) (CD200 receptor-like 2) (HuCD200R2) (CD200 cell surface glycoprotein receptor-like a) (CD200RLa) (Cell surface glycoprotein CD200 receptor 1-like) (Cell surface glycoprotein OX2 receptor 2)
CD47	Leukocyte surface antigen CD47 (Antigenic surface determinant protein OA3) (Integrin-associated protein) (IAP) (Protein MER6) (CD antigen CD47)
CD81	CD81 antigen (26 kDa cell surface protein TAPA-1) (Target of the antiproliferative antibody 1) (Tetraspanin-28) (Tspan-28) (CD antigen CD81)
CDH5	Cadherin-5 (7B4 antigen) (Vascular endothelial cadherin) (VE-cadherin) (CD antigen CD144)
CDK19	Cyclin-dependent kinase 19 (EC 2.7.11.22) (CDC2-related protein kinase 6) (Cell division cycle 2-like protein kinase 6) (Cell division protein kinase 19) (Cyclin-dependent kinase 11) (Death-preventing kinase)
CEBPA	CCAAT/enhancer-binding protein alpha (C/EBP alpha)
CELF1	CUGBP Elav-like family member 1 (CELF-1) (50 kDa nuclear polyadenylated RNA-binding protein) (Bruno-like protein 2) (CUG triplet repeat RNA-binding protein 1) (CUG-BP1) (CUG-BP- and ETR-3-like factor 1) (Deadenylation factor CUG-BP) (Embryo deadenylation element-binding protein homolog) (EDEN-BP homolog) (RNA-binding protein BRUNOL-2)
CLOCK	Circadian locomoter output cycles protein kaput (hCLOCK) (EC 2.3.1.48) (Class E basic helix-loop-helix protein 8) (bHLHe8)
CMA1	Chymase (EC 3.4.21.39) (Alpha-chymase) (Mast cell protease I)
CNR1	Cannabinoid receptor 1 (CB-R) (CB1) (CANN6)

CNR2	Cannabinoid receptor 2 (CB-2) (CB2) (hCB2) (CX5)
CREB3L3	Cyclic AMP-responsive element-binding protein 3-like protein 3 (cAMP-responsive element-binding protein 3-like protein 3) (Transcription factor CREB-H) [Cleaved into: Processed cyclic AMP-responsive element-binding protein 3-like protein 3]
CST7	Cystatin-F (Cystatin-7) (Cystatin-like metastasis-associated protein) (CMAP) (Leukocystatin)
CTSC	Dipeptidyl peptidase 1 (EC 3.4.14.1) (Cathepsin C) (Cathepsin J) (Dipeptidyl peptidase I) (DPP-I) (DPPI) (Dipeptidyl transferase) [Cleaved into: Dipeptidyl peptidase 1 exclusion domain chain (Dipeptidyl peptidase I exclusion domain chain); Dipeptidyl peptidase 1 heavy chain (Dipeptidyl peptidase I heavy chain); Dipeptidyl peptidase 1 light chain (Dipeptidyl peptidase I light chain)]
CXCL17	C-X-C motif chemokine 17 (6-Cys CXCL17) (Dendritic cell and monocyte chemokine-like protein) (DMC) (VEGF coregulated chemokine 1) [Cleaved into: 4-Cys CXCL17]
CYLD	Ubiquitin carboxyl-terminal hydrolase CYLD (EC 3.4.19.12) (Deubiquitinating enzyme CYLD) (Ubiquitin thioesterase CYLD) (Ubiquitin-specific-processing protease CYLD)
DAGLA	Diacylglycerol lipase-alpha (DAGL-alpha) (DGL-alpha) (EC 3.1.1.-) (Neural stem cell-derived dendrite regulator) (Sn1-specific diacylglycerol lipase alpha)
DAGLB	Diacylglycerol lipase-beta (DAGL-beta) (DGL-beta) (EC 3.1.1.-) (KCCR13L) (PUFA-specific triacylglycerol lipase) (EC 3.1.1.3) (Sn1-specific diacylglycerol lipase beta)
DDT	D-dopachrome decarboxylase (EC 4.1.1.84) (D-dopachrome tautomerase) (Phenylpyruvate tautomerase II)
DEFB114	Beta-defensin 114 (Beta-defensin 14) (DEFB-14) (Defensin, beta 114)
DHX9	ATP-dependent RNA helicase A (EC 3.6.4.13) (DEAH box protein 9) (DExH-box helicase 9) (Leukophysin) (LKP) (Nuclear DNA helicase II) (NDH II) (RNA helicase A)
DNASE1	Deoxyribonuclease-1 (EC 3.1.21.1) (Deoxyribonuclease I) (DNase I) (Dornase alfa)
DNASE1L3	Deoxyribonuclease gamma (DNase gamma) (EC 3.1.21.-) (DNase I homolog protein DHP2) (Deoxyribonuclease I-like 3) (DNase I-like 3) (Liver and spleen DNase) (LS-DNase) (LSD)
DROSHA	Ribonuclease 3 (EC 3.1.26.3) (Protein Drosha) (Ribonuclease III) (RNase III) (p241)
DUOXA1	Dual oxidase maturation factor 1 (Dual oxidase activator 1) (Numb-interacting protein)
DUOXA2	Dual oxidase maturation factor 2 (Dual oxidase activator 2)
DUSP10	Dual specificity protein phosphatase 10 (EC 3.1.3.16) (EC 3.1.3.48) (Mitogen-activated protein kinase phosphatase 5) (MAP kinase phosphatase 5) (MKP-5)

EDNRB	Endothelin receptor type B (ET-B) (ET-BR) (Endothelin receptor non-selective type)
EGFR	Epidermal growth factor receptor (EC 2.7.10.1) (Proto-oncogene c-ErbB-1) (Receptor tyrosine-protein kinase erbB-1)
ELANE	Neutrophil elastase (EC 3.4.21.37) (Bone marrow serine protease) (Elastase-2) (Human leukocyte elastase) (HLE) (Medullasin) (PMN elastase)
ENPP3	Ectonucleotide pyrophosphatase/phosphodiesterase family member 3 (E-NPP 3) (NPP3) (Phosphodiesterase I beta) (PD-Ibeta) (Phosphodiesterase I/nucleotide pyrophosphatase 3) (CD antigen CD203c) [Includes: Alkaline phosphodiesterase I (EC 3.1.4.1); Nucleotide pyrophosphatase (NPPase) (EC 3.6.1.9) (Nucleotide diphosphatase)]
ESR1	Estrogen receptor (ER) (ER-alpha) (Estradiol receptor) (Nuclear receptor subfamily 3 group A member 1)
ETS1	Protein C-ets-1 (p54)
F12	Coagulation factor XII (EC 3.4.21.38) (Hageman factor) (HAF) [Cleaved into: Coagulation factor XIIa heavy chain; Beta-factor XIIa part 1; Coagulation factor XIIa light chain (Beta-factor XIIa part 2)]
FABP4	Fatty acid-binding protein, adipocyte (Adipocyte lipid-binding protein) (ALBP) (Adipocyte-type fatty acid-binding protein) (A-FABP) (AFABP) (Fatty acid-binding protein 4)
FANCA	Fanconi anemia group A protein (Protein FACA)
FANCD2	Fanconi anemia group D2 protein (Protein FACD2)
FCGR2B	Low affinity immunoglobulin gamma Fc region receptor II-b (IgG Fc receptor II-b) (CDw32) (Fc-gamma RII-b) (Fc-gamma-RIIb) (FcRII-b) (CD antigen CD32)
FEM1A	Protein fem-1 homolog A (FEM1a) (FEM1-alpha) (Prostaglandin E receptor 4-associated protein)
FFAR3	Free fatty acid receptor 3 (G-protein coupled receptor 41)
FFAR4	Free fatty acid receptor 4 (G-protein coupled receptor 120) (G-protein coupled receptor 129) (G-protein coupled receptor GT01) (G-protein coupled receptor PGR4) (Omega-3 fatty acid receptor 1)
FNDC4	Fibronectin type III domain-containing protein 4 (Fibronectin type III repeat-containing protein 1)
FOXF1	Forkhead box protein F1 (Forkhead-related activator 1) (FREAC-1) (Forkhead-related protein FKHL5) (Forkhead-related transcription factor 1)
FOXP1	Forkhead box protein P1 (Mac-1-regulated forkhead) (MFH)
FOXP3	Forkhead box protein P3 (Scurfin) [Cleaved into: Forkhead box protein P3, C-terminally processed; Forkhead box protein P3 41 kDa form]
FUT7	Alpha-(1,3)-fucosyltransferase 7 (EC 2.4.1.-) (Fucosyltransferase 7) (Fucosyltransferase VII) (Fuc-TVII) (FucT-VII) (Galactoside 3-L-fucosyltransferase) (Selectin ligand synthase)

GATA3	Trans-acting T-cell-specific transcription factor GATA-3 (GATA-binding factor 3)
GBA	Lysosomal acid glucosylceramidase (Lysosomal acid GCase) (EC 3.2.1.45) (Acid beta-glucosidase) (Alglucerase) (Beta-glucocerebrosidase) (Beta-GC) (Cholesterol glucosyltransferase) (SGTase) (EC 2.4.1.-) (Cholesteryl-beta-glucosidase) (EC 3.2.1.-) (D-glucosyl-N-acylsphingosine glucohydrolase) (Imiglucerase)
GGT1	Glutathione hydrolase 1 proenzyme (EC 3.4.19.13) (Gamma-glutamyltransferase 1) (Gamma-glutamyltranspeptidase 1) (GGT 1) (EC 2.3.2.2) (Leukotriene-C4 hydrolase) (EC 3.4.19.14) (CD antigen CD224) [Cleaved into: Glutathione hydrolase 1 heavy chain; Glutathione hydrolase 1 light chain]
GGT2	Inactive glutathione hydrolase 2 (Gamma-glutamyltransferase 2) (Inactive gamma-glutamyltranspeptidase 2) (GGT 2)
GGT3P	Putative glutathione hydrolase 3 proenzyme (EC 3.4.19.13) (Gamma-glutamyltransferase 3) (Putative gamma-glutamyltranspeptidase 3) (GGT 3) (EC 2.3.2.2) [Cleaved into: Putative glutathione hydrolase 3 heavy chain; Putative glutathione hydrolase 3 light chain]
GHRL	Appetite-regulating hormone (Growth hormone secretagogue) (Growth hormone-releasing peptide) (Motilin-related peptide) (Protein M46) [Cleaved into: Ghrelin-27; Ghrelin-28 (Ghrelin); Obestatin]
GHSR	Growth hormone secretagogue receptor type 1 (GHS-R) (GH-releasing peptide receptor) (GHRP) (Ghrelin receptor)
GIT1	ARF GTPase-activating protein GIT1 (ARF GAP GIT1) (Cool-associated and tyrosine-phosphorylated protein 1) (CAT-1) (CAT1) (G protein-coupled receptor kinase-interactor 1) (GRK-interacting protein 1) (p95-APP1)
GPER1	G-protein coupled estrogen receptor 1 (Chemoattractant receptor-like 2) (Flow-induced endothelial G-protein coupled receptor 1) (FEG-1) (G protein-coupled estrogen receptor 1) (G-protein coupled receptor 30) (GPCR-Br) (IL8-related receptor DRY12) (Lymphocyte-derived G-protein coupled receptor) (LYGPR) (Membrane estrogen receptor) (mER)
GPR17	Uracil nucleotide/cysteinyl leukotriene receptor (UDP/CysLT receptor) (G-protein coupled receptor 17) (P2Y-like receptor) (R12)
GPR31	12-(S)-hydroxy-5,8,10,14-eicosatetraenoic acid receptor (12-(S)-HETE receptor) (12-HETER) (G-protein coupled receptor 31) (GPR31/12-HETER)
GPR4	G-protein coupled receptor 4 (G-protein coupled receptor 6C.I) (GPR6C.I)
GPRC5B	G-protein coupled receptor family C group 5 member B (A-69G12.1) (Retinoic acid-induced gene 2 protein) (RAIG-2)
GPS2	G protein pathway suppressor 2 (GPS-2)
GPSM3	G-protein-signaling modulator 3 (Activator of G-protein signaling

	4) (G18.1b) (Protein G18)
GPX1	Glutathione peroxidase 1 (GPx-1) (GSHPx-1) (EC 1.11.1.9) (Cellular glutathione peroxidase)
GRN	Progranulin (PGRN) (Acrogranin) (Epithelin precursor) (Glycoprotein of 88 Kda) (GP88) (Glycoprotein 88) (Granulin precursor) (PC cell-derived growth factor) (PCDGF) (Proepithelin) (PEPI) [Cleaved into: Paragranulin; Granulin-1 (Granulin G); Granulin-2 (Granulin F); Granulin-3 (Epithelin-2) (Granulin B); Granulin-4 (Epithelin-1) (Granulin A); Granulin-5 (Granulin C); Granulin-6 (Granulin D); Granulin-7 (Granulin E)]
GSTP1	Glutathione S-transferase P (EC 2.5.1.18) (GST class-pi) (GSTP1-1)
HAMP	Hepcidin (Liver-expressed antimicrobial peptide 1) (LEAP-1) (Putative liver tumor regressor) (PLTR) [Cleaved into: Hepcidin-25 (Hepc25); Hepcidin-20 (Hepc20)]
HCK	Tyrosine-protein kinase HCK (EC 2.7.10.2) (Hematopoietic cell kinase) (Hemopoietic cell kinase) (p59-HCK/p60-HCK) (p59Hck) (p61Hck)
HGF	Hepatocyte growth factor (Hepatopoietin-A) (Scatter factor) (SF) [Cleaved into: Hepatocyte growth factor alpha chain; Hepatocyte growth factor beta chain]
HLA-DRB1	HLA class II histocompatibility antigen, DRB1 beta chain (Human leukocyte antigen DRB1) (HLA-DRB1)
HLA-E	HLA class I histocompatibility antigen, alpha chain E (MHC class I antigen E) [Cleaved into: Soluble HLA class I histocompatibility antigen, alpha chain E (sHLA-E)]
HYAL2	Hyaluronidase-2 (Hyal-2) (EC 3.2.1.35) (Hyaluronoglucosaminidase-2) (Lung carcinoma protein 2) (LuCa-2)
IDO1	Indoleamine 2,3-dioxygenase 1 (IDO-1) (EC 1.13.11.52) (Indoleamine-pyrrole 2,3-dioxygenase)
IFI35	Interferon-induced 35 kDa protein (IFP 35) (Ifi-35)
IGF1	Insulin-like growth factor I (IGF-I) (Mechano growth factor) (MGF) (Somatomedin-C)
IL13	Interleukin-13 (IL-13)
IL16	Pro-interleukin-16 [Cleaved into: Interleukin-16 (IL-16) (Lymphocyte chemoattractant factor) (LCF)]
IL1RL1	Interleukin-1 receptor-like 1 (EC 3.2.2.6) (Protein ST2)
IL1RL2	Interleukin-1 receptor-like 2 (EC 3.2.2.6) (IL-36 receptor) (IL-36R) (Interleukin-1 receptor-related protein 2) (IL-1Rrp2) (IL1R-rp2)
IL20	Interleukin-20 (IL-20) (Cytokine Zcyto10)
IL20RB	Interleukin-20 receptor subunit beta (IL-20 receptor subunit beta) (IL-20R-beta) (IL-20RB) (Fibronectin type III domain containing 6) (FNDC6) (IL-20R2)
IL21	Interleukin-21 (IL-21) (Za11)

IL22RA2	Interleukin-22 receptor subunit alpha-2 (IL-22 receptor subunit alpha-2) (IL-22R-alpha-2) (IL-22RA2) (Cytokine receptor class-II member 10) (Cytokine receptor family 2 member 10) (CRF2-10) (Cytokine receptor family type 2, soluble 1) (CRF2-S1) (Interleukin-22-binding protein) (IL-22BP) (IL22BP) (ZcytoR16)
IL23A	Interleukin-23 subunit alpha (IL-23 subunit alpha) (IL-23-A) (Interleukin-23 subunit p19) (IL-23p19)
IL33	Interleukin-33 (IL-33) (Interleukin-1 family member 11) (IL-1F11) (Nuclear factor from high endothelial venules) (NF-HEV) [Cleaved into: Interleukin-33 (95-270); Interleukin-33 (99-270); Interleukin-33 (109-270)]
IL37	Interleukin-37 (IL-37) (FIL1 zeta) (IL-1X) (Interleukin-1 family member 7) (IL-1F7) (Interleukin-1 homolog 4) (IL-1H) (IL-1H4) (Interleukin-1 zeta) (IL-1 zeta) (Interleukin-1-related protein) (IL-1RP1) (Interleukin-23) (IL-23)
IL6ST	Interleukin-6 receptor subunit beta (IL-6 receptor subunit beta) (IL-6R subunit beta) (IL-6R-beta) (IL-6RB) (CDw130) (Interleukin-6 signal transducer) (Membrane glycoprotein 130) (gp130) (Oncostatin-M receptor subunit alpha) (CD antigen CD130)
INS	Insulin [Cleaved into: Insulin B chain; Insulin A chain]
IRF3	Interferon regulatory factor 3 (IRF-3)
ISL1	Insulin gene enhancer protein ISL-1 (Islet-1)
ITGA2	Integrin alpha-2 (CD49 antigen-like family member B) (Collagen receptor) (Platelet membrane glycoprotein Ia) (GPIa) (VLA-2 subunit alpha) (CD antigen CD49b)
JAK2	Tyrosine-protein kinase JAK2 (EC 2.7.10.2) (Janus kinase 2) (JAK-2)
KARS1	Lysine--tRNA ligase (EC 2.7.7.-) (EC 6.1.1.6) (Lysyl-tRNA synthetase) (LysRS)
KLF4	Krueppel-like factor 4 (Epithelial zinc finger protein EZF) (Gut-enriched krueppel-like factor)
KLKB1	Plasma kallikrein (EC 3.4.21.34) (Fletcher factor) (Kininogenin) (Plasma prekallikrein) (PKK) [Cleaved into: Plasma kallikrein heavy chain; Plasma kallikrein light chain]
KRT1	Keratin, type II cytoskeletal 1 (67 kDa cytokeratin) (Cytokeratin-1) (CK-1) (Hair alpha protein) (Keratin-1) (K1) (Type-II keratin Kb1)
LACC1	Purine nucleoside phosphorylase LACC1 (EC 2.4.2.1) (Adenosine deaminase LACC1) (EC 3.5.4.4) (Fatty acid metabolism-immunity nexus) (Guanosine phosphorylase LACC1) (Laccase domain-containing protein 1) (S-methyl-5'-thioadenosine phosphorylase LACC1) (EC 2.4.2.28)
LBP	Lipopolysaccharide-binding protein (LBP)
LILRA5	Leukocyte immunoglobulin-like receptor subfamily A member 5 (CD85 antigen-like family member F) (Immunoglobulin-like transcript 11) (ILT-11) (Leukocyte immunoglobulin-like receptor 9) (LIR-9) (CD antigen CD85f)

LPCAT3	Lysophospholipid acyltransferase 5 (LPLAT 5) (EC 2.3.1.-) (1-acylglycerophosphocholine O-acyltransferase) (EC 2.3.1.23) (1-acylglycerophosphoethanolamine O-acyltransferase) (EC 2.3.1.n7) (1-acylglycerophosphoserine O-acyltransferase) (EC 2.3.1.n6) (Lysophosphatidylcholine acyltransferase) (LPCAT) (Lyso-PC acyltransferase) (Lysophosphatidylcholine acyltransferase 3) (Lyso-PC acyltransferase 3) (Lysophosphatidylserine acyltransferase) (LPSAT) (Lyso-PS acyltransferase) (Membrane-bound O-acyltransferase domain-containing protein 5) (O-acyltransferase domain-containing protein 5)
LPL	Lipoprotein lipase (LPL) (EC 3.1.1.34) (Phospholipase A1) (EC 3.1.1.32)
LRFN5	Leucine-rich repeat and fibronectin type-III domain-containing protein 5
LRRC19	Leucine-rich repeat-containing protein 19
LRRK2	Leucine-rich repeat serine/threonine-protein kinase 2 (EC 2.7.11.1) (EC 3.6.5.-) (Dardarin)
MACIR	Macrophage immunometabolism regulator
MAPK13	Mitogen-activated protein kinase 13 (MAP kinase 13) (MAPK 13) (EC 2.7.11.24) (Mitogen-activated protein kinase p38 delta) (MAP kinase p38 delta) (Stress-activated protein kinase 4)
MAPK7	Mitogen-activated protein kinase 7 (MAP kinase 7) (MAPK 7) (EC 2.7.11.24) (Big MAP kinase 1) (BMK-1) (Extracellular signal-regulated kinase 5) (ERK-5)
MAS1	Proto-oncogene Mas
MCPH1	Microcephalin
MDK	Midkine (MK) (Amphiregulin-associated protein) (ARAP) (Midgestation and kidney protein) (Neurite outgrowth-promoting factor 2) (Neurite outgrowth-promoting protein)
METRNL	Meteorin-like protein (Subfatin)
MFHAS1	Malignant fibrous histiocytoma-amplified sequence 1 (Malignant fibrous histiocytoma-amplified sequence with leucine-rich tandem repeats 1)
MGST2	Microsomal glutathione S-transferase 2 (Microsomal GST-2) (EC 2.5.1.18) (Glutathione peroxidase MGST2) (EC 1.11.1.-) (Leukotriene C4 synthase MGST2) (EC 4.4.1.20) (Microsomal glutathione S-transferase II) (Microsomal GST-II)
MMP26	Matrix metalloproteinase-26 (MMP-26) (EC 3.4.24.-) (Endometase) (Matrilysin-2)
MMP3	Stromelysin-1 (SL-1) (EC 3.4.24.17) (Matrix metalloproteinase-3) (MMP-3) (Transin-1)
MMP8	Neutrophil collagenase (EC 3.4.24.34) (Matrix metalloproteinase-8) (MMP-8) (PMNL collagenase) (PMNL-CL)
MMP9	Matrix metalloproteinase-9 (MMP-9) (EC 3.4.24.35) (92 kDa gelatinase) (92 kDa type IV collagenase) (Gelatinase B) (GELB) [Cleaved into: 67 kDa matrix metalloproteinase-9; 82 kDa matrix

	metalloproteinase-9]
MVK	Mevalonate kinase (MK) (EC 2.7.1.36)
MYD88	Myeloid differentiation primary response protein MyD88
NAPEPLD	N-acyl-phosphatidylethanolamine-hydrolyzing phospholipase D (N-acyl phosphatidylethanolamine phospholipase D) (NAPE-PLD) (NAPE-hydrolyzing phospholipase D) (EC 3.1.4.54)
NDFIP1	NEDD4 family-interacting protein 1 (Breast cancer-associated protein SGA-1M) (NEDD4 WW domain-binding protein 5) (Putative MAPK-activating protein PM13) (Putative NF-kappa-B-activating protein 164) (Putative NFKB and MAPK-activating protein)
NFKBIZ	NF-kappa-B inhibitor zeta (I-kappa-B-zeta) (IkB-zeta) (IkappaBzeta) (IL-1 inducible nuclear ankyrin-repeat protein) (INAP) (Molecule possessing ankyrin repeats induced by lipopolysaccharide) (MAIL)
NLRC3	NLR family CARD domain-containing protein 3 (CARD15-like protein) (Caterpiller protein 16.2) (CLR16.2) (NACHT, LRR and CARD domains-containing protein 3) (Nucleotide-binding oligomerization domain protein 3)
NLRP1	NACHT, LRR and PYD domains-containing protein 1 (EC 3.4.-.-) (EC 3.6.4.-) (Caspase recruitment domain-containing protein 7) (Death effector filament-forming ced-4-like apoptosis protein) (Nucleotide-binding domain and caspase recruitment domain) [Cleaved into: NACHT, LRR and PYD domains-containing protein 1, C-terminus (NLRP1-CT); NACHT, LRR and PYD domains-containing protein 1, N-terminus (NLRP1-NT)]
NLRP10	NACHT, LRR and PYD domains-containing protein 10 (Nucleotide-binding oligomerization domain protein 8)
NLRP12	NACHT, LRR and PYD domains-containing protein 12 (Monarch-1) (PYRIN-containing APAF1-like protein 7) (Regulated by nitric oxide)
NLRP6	NACHT, LRR and PYD domains-containing protein 6 (Angiotensin II/vasopressin receptor) (PYRIN-containing APAF1-like protein 5)
NLRX1	NLR family member X1 (Caterpiller protein 11.3) (CLR11.3) (Nucleotide-binding oligomerization domain protein 26) (Nucleotide-binding oligomerization domain protein 5) (Nucleotide-binding oligomerization domain protein 9)
NPY5R	Neuropeptide Y receptor type 5 (NPY5-R) (NPY-Y5 receptor) (NPYY5-R) (Y5 receptor)
NR1D1	Nuclear receptor subfamily 1 group D member 1 (Rev-erbA-alpha) (V-erbA-related protein 1) (EAR-1)
NR1D2	Nuclear receptor subfamily 1 group D member 2 (Orphan nuclear hormone receptor BD73) (Rev-erb alpha-related receptor) (RVR) (Rev-erb-beta) (V-erbA-related protein 1-related) (EAR-1R)
NR1H3	Oxysterols receptor LXR-alpha (Liver X receptor alpha) (Nuclear

	receptor subfamily 1 group H member 3)
NR1H4	Bile acid receptor (Farnesoid X-activated receptor) (Farnesol receptor HRR-1) (Nuclear receptor subfamily 1 group H member 4) (Retinoid X receptor-interacting protein 14) (RXR-interacting protein 14)
NT5E	5'-nucleotidase (5'-NT) (EC 3.1.3.5) (Ecto-5'-nucleotidase) (CD antigen CD73)
NUPR1	Nuclear protein 1 (Candidate of metastasis 1) (Protein p8)
OTULIN	Ubiquitin thioesterase otulin (EC 3.4.19.12) (Deubiquitinating enzyme otulin) (OTU domain-containing deubiquitinase with linear linkage specificity) (Ubiquitin thioesterase Gumby)
PARK7	Parkinson disease protein 7 (Maillard deglycase) (Oncogene DJ1) (Parkinsonism-associated deglycase) (Protein DJ-1) (DJ-1) (Protein/nucleic acid deglycase DJ-1) (EC 3.1.2.-) (EC 3.5.1.-) (EC 3.5.1.124)
PBK	Lymphokine-activated killer T-cell-originated protein kinase (EC 2.7.12.2) (Cancer/testis antigen 84) (CT84) (MAPKK-like protein kinase) (Nori-3) (PDZ-binding kinase) (Spermatogenesis-related protein kinase) (SPK) (T-LAK cell-originated protein kinase)
PDCD4	Programmed cell death protein 4 (Neoplastic transformation inhibitor protein) (Nuclear antigen H731-like) (Protein 197/15a)
PDE2A	cGMP-dependent 3',5'-cyclic phosphodiesterase (EC 3.1.4.17) (Cyclic GMP-stimulated phosphodiesterase) (CGS-PDE) (cGSPDE)
PGLYRP2	N-acetylmuramoyl-L-alanine amidase (EC 3.5.1.28) (Peptidoglycan recognition protein 2) (Peptidoglycan recognition protein long) (PGRP-L)
PIK3AP1	Phosphoinositide 3-kinase adapter protein 1 (B-cell adapter for phosphoinositide 3-kinase) (B-cell phosphoinositide 3-kinase adapter protein 1)
PIK3CG	Phosphatidylinositol 4,5-bisphosphate 3-kinase catalytic subunit gamma isoform (PI3-kinase subunit gamma) (PI3K-gamma) (PI3Kgamma) (PtdIns-3-kinase subunit gamma) (EC 2.7.1.137) (EC 2.7.1.153) (EC 2.7.1.154) (Phosphatidylinositol 4,5-bisphosphate 3-kinase 110 kDa catalytic subunit gamma) (PtdIns-3-kinase subunit p110-gamma) (p110gamma) (Phosphoinositide-3-kinase catalytic gamma polypeptide) (Serine/threonine protein kinase PIK3CG) (EC 2.7.11.1) (p120-PI3K)
PLA2G2A	Phospholipase A2, membrane associated (EC 3.1.1.4) (GIIC sPLA2) (Group IIA phospholipase A2) (Non-pancreatic secretory phospholipase A2) (NPS-PLA2) (Phosphatidylcholine 2-acylhydrolase 2A)
PLA2G2D	Group IID secretory phospholipase A2 (GIID sPLA2) (sPLA2-IID) (EC 3.1.1.4) (PLA2IID) (Phosphatidylcholine 2-acylhydrolase 2D) (Secretory-type PLA, stroma-associated homolog)
PLA2G3	Group 3 secretory phospholipase A2 (EC 3.1.1.4) (Group III secretory phospholipase A2) (GIII sPLA2) (sPLA2-III)

	(Phosphatidylcholine 2-acylhydrolase 3)
PPARA	Peroxisome proliferator-activated receptor alpha (PPAR-alpha) (Nuclear receptor subfamily 1 group C member 1)
PPARD	Peroxisome proliferator-activated receptor delta (PPAR-delta) (NUCI) (Nuclear hormone receptor 1) (NUC1) (Nuclear receptor subfamily 1 group C member 2) (Peroxisome proliferator-activated receptor beta) (PPAR-beta)
PPARG	Peroxisome proliferator-activated receptor gamma (PPAR-gamma) (Nuclear receptor subfamily 1 group C member 3)
PRKCD	Protein kinase C delta type (EC 2.7.11.13) (Tyrosine-protein kinase PRKCD) (EC 2.7.10.2) (nPKC-delta) [Cleaved into: Protein kinase C delta type regulatory subunit; Protein kinase C delta type catalytic subunit (Sphingosine-dependent protein kinase-1) (SDK1)]
PROC	Vitamin K-dependent protein C (EC 3.4.21.69) (Anticoagulant protein C) (Autoprothrombin IIA) (Blood coagulation factor XIV) [Cleaved into: Vitamin K-dependent protein C light chain; Vitamin K-dependent protein C heavy chain; Activation peptide]
PSMA1	Proteasome subunit alpha type-1 (30 kDa prosomal protein) (PROS-30) (Macropain subunit C2) (Multicatalytic endopeptidase complex subunit C2) (Proteasome component C2) (Proteasome nu chain)
PSMA6	Proteasome subunit alpha type-6 (27 kDa prosomal protein) (PROS-27) (p27K) (Macropain iota chain) (Multicatalytic endopeptidase complex iota chain) (Proteasome iota chain)
PSMB4	Proteasome subunit beta type-4 (26 kDa prosomal protein) (HsBPROS26) (PROS-26) (Macropain beta chain) (Multicatalytic endopeptidase complex beta chain) (Proteasome beta chain) (Proteasome chain 3) (HsN3)
PTGER3	Prostaglandin E2 receptor EP3 subtype (PGE receptor EP3 subtype) (PGE2 receptor EP3 subtype) (PGE2-R) (Prostanoid EP3 receptor)
PTGES	Prostaglandin E synthase (EC 5.3.99.3) (Glutathione peroxidase PTGES) (EC 1.11.1.-) (Glutathione transferase PTGES) (EC 2.5.1.18) (Microsomal glutathione S-transferase 1-like 1) (MGST1-L1) (Microsomal prostaglandin E synthase 1) (MPGES-1) (p53-induced gene 12 protein)
PTGIS	Prostacyclin synthase (EC 5.3.99.4) (Hydroperoxy icosatetraenoate dehydratase) (EC 4.2.1.152) (Prostaglandin I2 synthase)
PTPN2	Tyrosine-protein phosphatase non-receptor type 2 (EC 3.1.3.48) (T-cell protein-tyrosine phosphatase) (TCPTP)
PTPRC	Receptor-type tyrosine-protein phosphatase C (EC 3.1.3.48) (Leukocyte common antigen) (L-CA) (T200) (CD antigen CD45)
PYCARD	Apoptosis-associated speck-like protein containing a CARD (hASC) (Caspase recruitment domain-containing protein 5) (PYD and CARD domain-containing protein) (Target of methylation-induced silencing 1)

PYDC2	Pyrin domain-containing protein 2 (Pyrin-only protein 2) (cellular POP2) (cPOP2)
RB1	Retinoblastoma-associated protein (p105-Rb) (p110-RB1) (pRb) (Rb) (pp110)
RHBDD3	Rhomboid domain-containing protein 3
RICTOR	Rapamycin-insensitive companion of mTOR (AVO3 homolog) (hAVO3)
RIPK1	Receptor-interacting serine/threonine-protein kinase 1 (EC 2.7.11.1) (Cell death protein RIP) (Receptor-interacting protein 1) (RIP-1)
RORA	Nuclear receptor ROR-alpha (Nuclear receptor RZR-alpha) (Nuclear receptor subfamily 1 group F member 1) (RAR-related orphan receptor A) (Retinoid-related orphan receptor-alpha)
RPS19	40S ribosomal protein S19 (Small ribosomal subunit protein eS19)
S100A8	Protein S100-A8 (Calgranulin-A) (Calprotectin L1L subunit) (Cystic fibrosis antigen) (CFAG) (Leukocyte L1 complex light chain) (Migration inhibitory factor-related protein 8) (MRP-8) (p8) (S100 calcium-binding protein A8) (Urinary stone protein band A)
SBNO2	Protein strawberry notch homolog 2
SCGB1A1	Uteroglobin (Clara cell phospholipid-binding protein) (CCPBP) (Clara cells 10 kDa secretory protein) (CC10) (Secretoglobin family 1A member 1) (Urinary protein 1) (UP-1) (UP1) (Urine protein 1)
SEMA7A	Semaphorin-7A (CDw108) (JMH blood group antigen) (John-Milton-Hargen human blood group Ag) (Semaphorin-K1) (Sema K1) (Semaphorin-L) (Sema L) (CD antigen CD108)
SERPINF1	Pigment epithelium-derived factor (PEDF) (Cell proliferation-inducing gene 35 protein) (EPC-1) (Serpin F1)
SETD6	N-lysine methyltransferase SETD6 (EC 2.1.1.-) (SET domain-containing protein 6)
SHARPIN	Sharpin (Shank-associated RH domain-interacting protein) (Shank-interacting protein-like 1) (hSIPL1)
SHPK	Sedoheptulokinase (SHK) (EC 2.7.1.14) (Carbohydrate kinase-like protein)
SIGLEC10	Sialic acid-binding Ig-like lectin 10 (Siglec-10) (Siglec-like protein 2)
SIRPA	Tyrosine-protein phosphatase non-receptor type substrate 1 (SHP substrate 1) (SHPS-1) (Brain Ig-like molecule with tyrosine-based activation motifs) (Bit) (CD172 antigen-like family member A) (Inhibitory receptor SHPS-1) (Macrophage fusion receptor) (MyD-1 antigen) (Signal-regulatory protein alpha-1) (Sirp-alpha-1) (Signal-regulatory protein alpha-2) (Sirp-alpha-2) (Signal-regulatory protein alpha-3) (Sirp-alpha-3) (p84) (CD antigen CD172a)
SLAMF8	SLAM family member 8 (B-lymphocyte activator macrophage expressed) (BCM-like membrane protein) (CD antigen CD353)

SMAD3	Mothers against decapentaplegic homolog 3 (MAD homolog 3) (Mad3) (Mothers against DPP homolog 3) (hMAD-3) (JV15-2) (SMAD family member 3) (SMAD 3) (Smad3) (hSMAD3)
SMPDL3B	Acid sphingomyelinase-like phosphodiesterase 3b (ASM-like phosphodiesterase 3b) (EC 3.1.4.-)
SNCA	Alpha-synuclein (Non-A beta component of AD amyloid) (Non-A4 component of amyloid precursor) (NACP)
SNX4	Sorting nexin-4
SOCS3	Suppressor of cytokine signaling 3 (SOCS-3) (Cytokine-inducible SH2 protein 3) (CIS-3) (STAT-induced STAT inhibitor 3) (SSI-3)
SOCS5	Suppressor of cytokine signaling 5 (SOCS-5) (Cytokine-inducible SH2 protein 6) (CIS-6) (Cytokine-inducible SH2-containing protein 5)
SOD1	Superoxide dismutase [Cu-Zn] (EC 1.15.1.1) (Superoxide dismutase 1) (hSod1)
SPATA2	Spermatogenesis-associated protein 2
STAP1	Signal-transducing adaptor protein 1 (STAP-1) (BCR downstream-signaling protein 1) (Docking protein BRDG1) (Stem cell adaptor protein 1)
STAT5B	Signal transducer and activator of transcription 5B
STING1	Stimulator of interferon genes protein (hSTING) (Endoplasmic reticulum interferon stimulator) (ERIS) (Mediator of IRF3 activation) (hMITA) (Transmembrane protein 173)
STK39	STE20/SPS1-related proline-alanine-rich protein kinase (Ste-20-related kinase) (EC 2.7.11.1) (DCHT) (Serine/threonine-protein kinase 39)
SUCNR1	Succinate receptor 1 (G-protein coupled receptor 91) (P2Y purinoceptor 1-like)
SYT11	Synaptotagmin-11 (Synaptotagmin XI) (SytXI)
TAC1	Protachykinin-1 (PPT) [Cleaved into: Substance P; Neurokinin A (NKA) (Neuromedin L) (Substance K); Neuropeptide K (NPK); Neuropeptide gamma; C-terminal-flanking peptide]
TAFA3	Chemokine-like protein TAFA-3
TEK	Angiopoietin-1 receptor (EC 2.7.10.1) (Endothelial tyrosine kinase) (Tunica interna endothelial cell kinase) (Tyrosine kinase with Ig and EGF homology domains-2) (Tyrosine-protein kinase receptor TEK) (Tyrosine-protein kinase receptor TIE-2) (hTIE2) (p140 TEK) (CD antigen CD202b)
TGM2	Protein-glutamine gamma-glutamyltransferase 2 (EC 2.3.2.13) (Erythrocyte transglutaminase) (Heart G alpha(h)) (hhG alpha(h)) (Isopeptidase TGM2) (EC 3.4.-.-) (Protein G alpha(h)) (G(h)) (Protein-glutamine deamidase TGM2) (EC 3.5.1.44) (Protein-glutamine dopaminyltransferase TGM2) (EC 2.3.1.-) (Protein-glutamine histaminyltransferase TGM2) (EC 2.3.1.-) (Protein-glutamine noradrenalinyltransferase TGM2) (EC 2.3.1.-) (Protein-glutamine serotonyltransferase TGM2) (EC 2.3.1.-) (Tissue

	transglutaminase) (tTG) (tTgase) (Transglutaminase C) (TG(C)) (TGC) (TGase C) (Transglutaminase H) (TGase H) (Transglutaminase II) (TGase II) (Transglutaminase-2) (TG2) (TGase-2) (hTG2)
TLR10	Toll-like receptor 10 (EC 3.2.2.6) (CD antigen CD290)
TLR4	Toll-like receptor 4 (EC 3.2.2.6) (hToll) (CD antigen CD284)
TLR7	Toll-like receptor 7
TLR9	Toll-like receptor 9 (CD antigen CD289)
TMSB4X	Thymosin beta-4 (T beta-4) (Fx) [Cleaved into: Hematopoietic system regulatory peptide (Seraspenide)]
TNF	Tumor necrosis factor (Cachectin) (TNF-alpha) (Tumor necrosis factor ligand superfamily member 2) (TNF-a) [Cleaved into: Tumor necrosis factor, membrane form (N-terminal fragment) (NTF); Intracellular domain 1 (ICD1); Intracellular domain 2 (ICD2); C-domain 1; C-domain 2; Tumor necrosis factor, soluble form]
TNFAIP3	Tumor necrosis factor alpha-induced protein 3 (TNF alpha-induced protein 3) (EC 2.3.2.-) (EC 3.4.19.12) (OTU domain-containing protein 7C) (Putative DNA-binding protein A20) (Zinc finger protein A20) [Cleaved into: A20p50; A20p37]
TNFAIP8L2	Tumor necrosis factor alpha-induced protein 8-like protein 2 (TIPE2) (TNF alpha-induced protein 8-like protein 2) (TNFAIP8-like protein 2) (Inflammation factor protein 20)
TNFRSF11A	Tumor necrosis factor receptor superfamily member 11A (Osteoclast differentiation factor receptor) (ODFR) (Receptor activator of NF-KB) (CD antigen CD265)
TNFSF11	Tumor necrosis factor ligand superfamily member 11 (Osteoclast differentiation factor) (ODF) (Osteoprotegerin ligand) (OPGL) (Receptor activator of nuclear factor kappa-B ligand) (RANKL) (TNF-related activation-induced cytokine) (TRANCE) (CD antigen CD254) [Cleaved into: Tumor necrosis factor ligand superfamily member 11, membrane form; Tumor necrosis factor ligand superfamily member 11, soluble form]
TNFSF18	Tumor necrosis factor ligand superfamily member 18 (Activation-inducible TNF-related ligand) (AITRL) (Glucocorticoid-induced TNF-related ligand) (hGITRL)
TNFSF4	Tumor necrosis factor ligand superfamily member 4 (Glycoprotein Gp34) (OX40 ligand) (OX40L) (TAX transcriptionally-activated glycoprotein 1) (CD antigen CD252)
TNIP1	TNFAIP3-interacting protein 1 (A20-binding inhibitor of NF-kappa-B activation 1) (ABIN-1) (HIV-1 Nef-interacting protein) (Nef-associated factor 1) (Naf1) (Nip40-1) (Virion-associated nuclear shuttling protein) (VAN) (hVAN)
TRADD	Tumor necrosis factor receptor type 1-associated DEATH domain protein (TNFR1-associated DEATH domain protein) (TNFRSF1A-

	associated via death domain)
TREM2	Triggering receptor expressed on myeloid cells 2 (TREM-2) (Triggering receptor expressed on monocytes 2)
TREX1	Three-prime repair exonuclease 1 (EC 3.1.11.2) (3'-5' exonuclease TREX1) (Deoxyribonuclease III) (DNase III)
TSLP	Thymic stromal lymphopoietin
TTBK1	Tau-tubulin kinase 1 (EC 2.7.11.1) (Brain-derived tau kinase)
TYRO3	Tyrosine-protein kinase receptor TYRO3 (EC 2.7.10.1) (Tyrosine-protein kinase BYK) (Tyrosine-protein kinase DTK) (Tyrosine-protein kinase RSE) (Tyrosine-protein kinase SKY) (Tyrosine-protein kinase TIF)
UFL1	E3 UFM1-protein ligase 1 (EC 2.3.2.-) (E3 UFM1-protein transferase 1) (Multiple alpha-helix protein located at ER) (Novel LZAP-binding protein) (Regulator of C53/LZAP and DDRGK1)
USP18	Ubl carboxyl-terminal hydrolase 18 (EC 3.4.19.-) (43 kDa ISG15-specific protease) (hUBP43) (ISG15-specific-processing protease) (Ubl thioesterase 18)
VAMP7	Vesicle-associated membrane protein 7 (VAMP-7) (Synaptobrevin-like protein 1) (Tetanus-insensitive VAMP) (Ti-VAMP)
VAMP8	Vesicle-associated membrane protein 8 (VAMP-8) (Endobrevin) (EDB)
VPS35	Vacuolar protein sorting-associated protein 35 (hVPS35) (Maternal-embryonic 3) (Vesicle protein sorting 35)
WFDC1	WAP four-disulfide core domain protein 1 (Prostate stromal protein ps20) (ps20 growth inhibitor)
WNT5A	Protein Wnt-5a
XCL1	Lymphotactin (ATAC) (C motif chemokine 1) (Cytokine SCM-1) (Lymphotaxin) (SCM-1-alpha) (Small-inducible cytokine C1) (XC chemokine ligand 1)
XIAP	E3 ubiquitin-protein ligase XIAP (EC 2.3.2.27) (Baculoviral IAP repeat-containing protein 4) (IAP-like protein) (ILP) (hILP) (Inhibitor of apoptosis protein 3) (IAP-3) (hIAP-3) (hIAP3) (RING-type E3 ubiquitin transferase XIAP) (X-linked inhibitor of apoptosis protein) (X-linked IAP)
ZBP1	Z-DNA-binding protein 1 (DNA-dependent activator of IFN-regulatory factors) (DAI) (Tumor stroma and activated macrophage protein DLM-1)
ZP3	Zona pellucida sperm-binding protein 3 (Sperm receptor) (ZP3A/ZP3B) (Zona pellucida glycoprotein 3) (Zp-3) (Zona pellucida protein C) [Cleaved into: Processed zona pellucida sperm-binding protein 3]
ZYX	Zyxin (Zyxin-2)
AIRE	Autoimmune regulator (Autoimmune polyendocrinopathy candidiasis ectodermal dystrophy protein) (APECED protein)

CARD8	Caspase recruitment domain-containing protein 8 (EC 3.4.-.-) (CARD-inhibitor of NF-kappa-B-activating ligand) (CARDINAL) (Tumor up-regulated CARD-containing antagonist of CASP9) (TUCAN) [Cleaved into: Caspase recruitment domain-containing protein 8, C-terminus (CARD8-CT); Caspase recruitment domain-containing protein 8, N-terminus (CARD8-NT)]
CASP10	Caspase-10 (CASP-10) (EC 3.4.22.63) (Apoptotic protease Mch-4) (FAS-associated death domain protein interleukin-1B-converting enzyme 2) (FLICE2) (ICE-like apoptotic protease 4) [Cleaved into: Caspase-10 subunit p23/17; Caspase-10 subunit p12]
CDH1	Cadherin-1 (CAM 120/80) (Epithelial cadherin) (E-cadherin) (Uvomorulin) (CD antigen CD324) [Cleaved into: E-Cad/CTF1; E-Cad/CTF2; E-Cad/CTF3]
CTLA4	Cytotoxic T-lymphocyte protein 4 (Cytotoxic T-lymphocyte-associated antigen 4) (CTLA-4) (CD antigen CD152)
FAS	Tumor necrosis factor receptor superfamily member 6 (Apo-1 antigen) (Apoptosis-mediating surface antigen FAS) (FASLG receptor) (CD antigen CD95)
FASLG	Tumor necrosis factor ligand superfamily member 6 (Apoptosis antigen ligand) (APTL) (CD95 ligand) (CD95-L) (Fas antigen ligand) (Fas ligand) (FasL) (CD antigen CD178) [Cleaved into: Tumor necrosis factor ligand superfamily member 6, membrane form; Tumor necrosis factor ligand superfamily member 6, soluble form (Receptor-binding FasL ectodomain) (Soluble Fas ligand) (sFasL); ADAM10-processed FasL form (APL); FasL intracellular domain (FasL ICD) (SPPL2A-processed FasL form) (SPA)]
IL1RN	Interleukin-1 receptor antagonist protein (IL-1RN) (IL-1ra) (IRAP) (ICIL-1RA) (IL1 inhibitor) (Anakinra)
KRAS	GTPase KRas (EC 3.6.5.2) (K-Ras 2) (Ki-Ras) (c-K-ras) (c-Ki-ras) [Cleaved into: GTPase KRas, N-terminally processed]
LRBA	Lipopolysaccharide-responsive and beige-like anchor protein (Beige-like protein) (CDC4-like protein)
SKIV2L	Helicase SKI2W (Ski2) (EC 3.6.4.-) (Helicase-like protein) (HLP)
TTC37	Tetratricopeptide repeat protein 37 (TPR repeat protein 37) (SKI3 homolog) (Ski3) (Tricho-hepatic-enteric syndrome protein) (Thespin)
