# Supplementary material for: Inflammatory Response-Related Long Non-Coding RNA Signature Predicts the Prognosis of Hepatocellular Carcinoma
Source: J Oncol. 2022 Mar 17;2022:9917244. doi: 10.1155/2022/9917244 (PMC8947866; doi:10.1155/2022/9917244)
Supplement: Supplementary Materials — Supplementary tables: Supplementary Table 1. Identified inflammatory response-related genes from the Molecular Signatures Database. Supplementary Table 2. The inflammatory response-related DEGs between HCC and noncancerous liver tissues. Supplementary Table 3. The results of univariate Cox regression, LASSO regression, and multivariate Cox regression analysis. Supplementary Table 4. The net benefit of risk score model in DCA analysis. Supplementary Table 5. The results of gene set enrichment analysis. Supplementary Table 6. The immune responses in low- and high-risk groups. [file 9917244.f1.zip › 9917244.f1/Table S2 (1).pdf]

Gene	conMean	treatMean	LogFC	P-value	FDR
COL1A1	8.8304491	50.3713981	2.512046056	6.53E-07	1.38E-06
COL1A2	5.88129243	26.0841594	2.148968809	2.69E-06	5.23E-06
IL2RG	3.44784832	8.05418417	1.224042155	0.01704743	0.02195426
LAMA5	1.53256749	5.20920481	1.765112549	7.85E-14	3.96E-13
LAMB1	4.00580318	10.8322513	1.435169675	1.48E-06	3.00E-06
LAMC1	4.31562374	18.7369357	2.118244035	1.87E-23	4.06E-22
THBS1	24.215344	11.9694003	-1.01657063	1.49E-09	4.60E-09
THBS3	1.21847326	3.46710356	1.508656343	5.02E-22	9.08E-21
ABCF1	10.3871564	20.8338599	1.004129396	1.20E-21	2.00E-20
ADORA2A	0.19041643	0.46872639	1.299588062	1.40E-11	5.20E-11
AGER	0.52663424	1.25773052	1.255949601	1.99E-15	1.26E-14
ALOX15	0.02190522	0.09618897	2.134596898	8.16E-06	1.46E-05
BDKRB2	0.24523742	0.95932145	1.967835206	8.58E-08	2.07E-07
CCL13	0.09686332	0.58239796	2.58798297	2.62E-06	5.17E-06
CCL20	8.06339551	31.0344083	1.944409248	9.83E-08	2.36E-07
CCL23	2.00939026	0.39236626	-2.3564849	4.04E-24	1.03E-22
CCL8	0.46638631	0.93770093	1.007602432	0.00037029	0.00056587
CXCL12	30.4835369	7.35533449	-2.05116745	1.08E-24	3.14E-23
CXCL14	15.1179578	2.26106575	-2.74118842	1.09E-26	7.91E-25
CXCR2	0.28385403	0.10358459	-1.45433974	4.27E-15	2.54E-14
FOS	113.200461	21.1632043	-2.41924984	6.68E-21	9.66E-20
FPR1	2.15173252	0.95298616	-1.17497158	2.27E-12	9.20E-12
FPR2	0.25961417	0.06410529	-2.01785386	6.95E-16	4.78E-15
IL1A	0.01072757	0.02485794	1.212383329	0.00735294	0.00994136
IL1B	0.87439016	0.41835073	-1.06356421	2.07E-10	6.81E-10
IL1RAP	6.72884044	2.74841651	-1.29175726	3.91E-16	2.74E-15
LYZ	28.3524811	189.743984	2.74250725	2.09E-07	4.83E-07
NFATC4	0.21778672	0.87842224	2.01199858	1.21E-12	5.19E-12
ORM1	6110.26438	2473.74284	-1.30453927	3.46E-14	1.88E-13
PLA2G7	1.73344392	4.02391491	1.214958635	0.00058441	0.00088374
PTGS2	0.68569549	0.20982015	-1.70841478	5.31E-17	4.52E-16
S100A12	1.31808196	0.25747248	-2.35594995	4.41E-17	3.83E-16
SAA1	3680.81904	1220.67577	-1.59234678	2.16E-06	4.31E-06
ADGRE1	0.54153292	0.22278723	-1.28138254	1.39E-15	9.13E-15
ADORA2B	0.18377306	0.48997165	1.414772971	0.04070331	0.04976122
ATP2A2	7.98290076	16.4297919	1.041329222	2.33E-16	1.65E-15
BDKRB1	0.03117418	0.34524509	3.469197321	5.68E-15	3.28E-14
BEST1	0.09195726	0.26101329	1.505087861	5.03E-07	1.08E-06
CALCRL	1.33513035	2.87867519	1.108424414	1.12E-05	1.98E-05
CD70	0.06003692	0.32140683	2.420478791	1.61E-05	2.81E-05
CHST2	0.4546287	1.075581	1.242355516	3.79E-07	8.18E-07
CSF3	0.06729959	0.01292198	-2.38077047	2.67E-07	5.95E-07
GPC3	2.92859571	271.542028	6.534822656	1.38E-21	2.22E-20
HRH1	0.14227397	0.34923623	1.295531481	2.92E-07	6.44E-07
IL6	0.86703163	0.3207223	-1.43475996	3.10E-05	5.26E-05

ITGA5	6.19318012	15.1131772	1.287054671	6.76E-14	3.45E-13
KCNJ2	0.19848594	0.43546862	1.13353196	2.25E-07	5.11E-07
LTA	0.10963536	0.26539008	1.275401304	1.78E-05	3.08E-05
LY6E	132.081847	49.9571976	-1.40266775	3.07E-18	3.41E-17
MARCO	36.1502595	1.84271253	-4.29410307	9.04E-28	1.31E-25
MEP1A	0.02274982	3.4973256	7.264253142	1.20E-09	3.78E-09
MMP14	5.29639824	23.1389453	2.1272396	7.85E-16	5.32E-15
NPFFR2	0.00512006	0.64927274	6.986521036	6.45E-08	1.60E-07
P2RX4	1.52132139	3.65077583	1.262878124	5.20E-14	2.75E-13
PROK2	0.13385585	0.03092918	-2.11363988	1.78E-12	7.41E-12
RIPK2	2.29385872	5.50604934	1.263241001	5.33E-12	2.08E-11
ROS1	0.00714379	0.07469284	3.386207659	0.00850003	0.01135081
SERPINE1	129.223087	45.0428304	-1.52049446	2.29E-11	8.14E-11
SGMS2	3.76599656	1.80259898	-1.06295321	1.68E-05	2.93E-05
SLC7A1	0.44787338	1.43318221	1.678059208	2.47E-05	4.23E-05
SLC7A2	46.3748972	21.3990689	-1.11579606	2.68E-17	2.48E-16
SPHK1	0.74170957	5.34771995	2.849997622	8.75E-05	0.00014383
TACR1	0.01548459	0.21639879	3.804787096	0.00021344	0.00033932
TNFAIP6	0.10548552	0.63422111	2.587940972	0.00749592	0.01010319
TNFRSF9	0.08315689	0.49048331	2.560296257	0.00021055	0.00033595
TNFSF15	0.05689787	0.34987734	2.620402681	6.61E-09	1.90E-08
TNFSF9	0.13301104	0.54973867	2.047199976	0.00033157	0.00051393
ABCC1	0.70715413	2.38037383	1.751091565	3.82E-06	7.24E-06
ABHD12	8.23208472	21.7011593	1.398442381	1.92E-25	9.25E-24
ADA	0.94339272	2.67245149	1.502233382	1.19E-17	1.14E-16
ADAMTS12	0.19144931	0.50548373	1.40070221	0.00090957	0.00135189
ASH1L	1.37855372	3.18842985	1.209690655	1.22E-12	5.19E-12
BAP1	7.2785839	15.1877553	1.061178967	1.68E-23	3.83E-22
BCL6B	0.9817647	2.56437265	1.385156734	5.77E-17	4.72E-16
BIRC3	3.46597188	9.33517115	1.429416522	0.00109749	0.00161461
C1QTNF12	0.1181424	0.59461404	2.331426681	1.53E-13	7.39E-13
C1QTNF3	0.3034175	7.44946294	4.617760199	3.84E-17	3.40E-16
CASP12	0.02164271	0.0908038	2.068871313	8.37E-14	4.18E-13
CCN3	0.30405312	1.12783873	1.8911655	2.18E-09	6.63E-09
CD200	0.20482772	1.16873268	2.512462082	6.38E-22	1.11E-20
CEBPA	28.6667306	70.4949378	1.298142284	2.30E-10	7.45E-10
CNR1	0.0183292	0.20117437	3.456230782	0.00119627	0.00174221
CTSC	3.05104049	7.47064296	1.291931089	2.92E-07	6.44E-07
CXCL17	0.06501956	1.98735421	4.933831438	1.06E-06	2.17E-06
DAGLA	0.22281287	0.94124545	2.078738415	8.66E-09	2.46E-08
DHX9	8.66952038	17.4969578	1.013080019	1.08E-17	1.06E-16
DNASE1	0.14879566	0.50542113	1.764153551	1.19E-22	2.25E-21
DNASE1L3	25.5616189	4.74900514	-2.42828199	4.85E-25	1.75E-23
DROSHA	1.68232684	4.13096714	1.296021567	1.89E-25	9.25E-24
DUOXA2	0.10609215	3.10312215	4.870330609	0.0007902	0.00118258
ESR1	2.14304757	0.55784331	-1.94173201	1.04E-22	2.06E-21

FABP4	0.99965822	9.58985503	3.262002174	4.89E-09	1.42E-08
FANCA	0.28464638	0.91971087	1.692009625	3.77E-18	4.09E-17
FANCD2	0.13155497	1.01642716	2.949769141	2.65E-25	1.15E-23
FCGR2B	1.75034176	0.81100618	-1.10985183	1.30E-18	1.52E-17
FFAR4	0.06373553	0.18584482	1.543928628	2.40E-05	4.13E-05
GBA	5.56522966	22.7008795	2.028235066	3.64E-28	7.90E-26
GIT1	1.82731661	5.78851545	1.663466769	2.48E-27	2.70E-25
GPR4	1.01247931	2.04170549	1.011882354	2.77E-12	1.11E-11
GPRC5B	1.407817	2.84166861	1.013278503	0.03408733	0.04226829
GPS2	0.32932085	0.77133147	1.227857132	7.10E-17	5.71E-16
GRN	43.1944594	99.8903719	1.20949936	9.80E-21	1.37E-19
HAMP	148.253103	15.9673704	-3.21486365	6.51E-25	2.17E-23
HGF	2.77514779	1.06262296	-1.3849348	3.52E-20	4.63E-19
IDO1	0.44430679	1.21390122	1.450022921	0.00036087	0.00055342
IL1RL1	0.96181571	0.14092557	-2.77082703	2.86E-14	1.57E-13
IRF3	6.1856349	15.8871483	1.360866598	9.34E-24	2.25E-22
ITGA2	0.20289888	1.33914506	2.722479454	2.68E-14	1.49E-13
KLKB1	57.6342058	27.4189604	-1.07175143	5.73E-18	6.07E-17
KRT1	0.287331	0.12562208	-1.19362354	0.01016093	0.01344464
LILRA5	1.93229268	0.6593845	-1.55112176	1.99E-16	1.44E-15
LPL	0.1415804	1.10297274	2.961703712	1.86E-21	2.88E-20
LRRC19	0.41982915	0.15368795	-1.44979831	2.07E-10	6.81E-10
MACIR	0.20851064	1.13878865	2.449307117	1.42E-07	3.34E-07
MAPK13	0.57805464	2.88465234	2.319119671	0.0083499	0.01118475
MAPK7	0.85358534	1.71958171	1.010450374	2.03E-15	1.26E-14
MDK	3.97261568	80.7214113	4.344790262	1.00E-24	3.11E-23
MIR126	0.24558019	1.07377045	2.128419497	3.56E-11	1.24E-10
MIR590	0.14998897	0.33948484	1.178490734	0.00393298	0.00545339
MMP3	0.04298973	0.21215586	2.303060741	0.01191328	0.01557339
MMP9	1.38857064	9.15353559	2.720728527	2.14E-08	5.67E-08
NEAT1	5.48179461	12.6457013	1.205926873	9.87E-09	2.76E-08
NLRP1	0.40595995	1.07178314	1.400603732	1.31E-09	4.10E-09
NUPR1	15.357615	49.080928	1.676208338	5.55E-14	2.90E-13
PBK	0.17084198	3.08537911	4.174713378	1.71E-26	1.06E-24
PPARD	3.89903328	8.03016429	1.042313035	1.51E-11	5.57E-11
PPARG	2.1864399	5.05184521	1.20822674	6.56E-11	2.28E-10
PRKCD	2.37024444	6.311841	1.413025014	8.94E-16	5.97E-15
PSMB4	53.7176744	140.798248	1.390160627	2.52E-28	7.90E-26
PTGER3	0.04380661	0.14936328	1.769605014	1.47E-05	2.58E-05
PTGES	0.29739607	3.20094565	3.428040699	0.01479769	0.01922813
PYCARD	3.24194386	9.79743175	1.59554451	7.65E-06	1.37E-05
RHBDD3	3.67999452	8.85212439	1.266320105	5.02E-23	1.04E-21
RPS19	71.7198928	197.130402	1.458705052	4.25E-19	5.27E-18
SEMA7A	0.66207899	2.38502152	1.84892702	9.40E-12	3.58E-11
SHARPIN	9.96384326	29.6454761	1.573037734	9.69E-27	7.91E-25
SLAMF8	1.06131599	2.52958336	1.253045529	3.44E-07	7.54E-07

SMAD3	2.66588764	5.68879664	1.093507534	8.62E-13	3.78E-12
SOCS3	40.4774342	12.8383622	-1.65665667	4.00E-10	1.29E-09
STK39	0.47072767	3.07631102	2.708236816	6.27E-14	3.24E-13
TAFA3	0.00732289	0.02714049	1.889961651	3.28E-05	5.52E-05
TLR9	0.0032034	0.01029693	1.684540215	2.65E-06	5.19E-06
TNFRSF11A	0.14542815	0.41890651	1.526321785	0.00029671	0.00046657
TNFSF4	0.19081647	1.02407932	2.424069882	1.01E-18	1.21E-17
TREM2	1.2006667	5.36833595	2.160639237	4.91E-13	2.22E-12
TSLP	1.37249065	0.4007576	-1.77599454	1.59E-20	2.16E-19
TYRO3	0.19667651	1.06489269	2.436811477	9.42E-07	1.96E-06
WNT5A	0.44658625	1.18224436	1.40451752	0.02260723	0.02860507
ZP3	0.17414994	0.82489446	2.243879573	1.22E-16	8.96E-16
ZYX	18.8067031	39.7235952	1.078749242	8.78E-13	3.81E-12
AIRE	0.00208565	0.02828784	3.761615355	3.97E-08	1.01E-07
CTLA4	0.17960284	0.58959253	1.714908093	6.29E-06	1.15E-05
IL1RN	42.1808179	14.7111798	-1.51967412	2.19E-11	7.87E-11
SKIV2L	5.19789892	11.5389807	1.150515308	3.21E-25	1.27E-23
