# Supplementary material for: Inflammatory Response-Related Long Non-Coding RNA Signature Predicts the Prognosis of Hepatocellular Carcinoma
Source: J Oncol. 2022 Mar 17;2022:9917244. doi: 10.1155/2022/9917244 (PMC8947866; doi:10.1155/2022/9917244)
Supplement: Supplementary Materials — Supplementary tables: Supplementary Table 1. Identified inflammatory response-related genes from the Molecular Signatures Database. Supplementary Table 2. The inflammatory response-related DEGs between HCC and noncancerous liver tissues. Supplementary Table 3. The results of univariate Cox regression, LASSO regression, and multivariate Cox regression analysis. Supplementary Table 4. The net benefit of risk score model in DCA analysis. Supplementary Table 5. The results of gene set enrichment analysis. Supplementary Table 6. The immune responses in low- and high-risk groups. [file 9917244.f1.zip › 9917244.f1/Table S3.pdf]

Univariate COX analysis				
LncRNA	HR	HR.95L	HR.95H	pvalue
LINC00205	1.234506631	1.101101652	1.384074414	0.000305496
AC026356.1	4.256425869	2.29654217	7.888886783	4.21E-06
RHPN1-AS1	1.680926528	1.255261828	2.250935964	0.000490411
SNHG26	2.484420844	1.624030606	3.800634613	2.72E-05
THUMPD3-AS1	1.381382487	1.181870992	1.614573494	4.92E-05
BACE1-AS	1.213342719	1.099544235	1.338918897	0.000118829
AC068987.3	1.121675274	1.052385713	1.195526892	0.000416449
AP001469.3	1.987115683	1.338184209	2.950736311	0.000663853
AC116025.2	1.888348845	1.371231478	2.600480968	9.87E-05
AL451069.3	1.132012729	1.052575374	1.217445183	0.000836929
C2orf27A	1.623010442	1.313300413	2.005758064	7.37E-06
AC124798.1	1.216415382	1.090012115	1.357477006	0.000465975
AC107959.3	1.348870015	1.138077168	1.59870558	0.000556876
AC068473.5	1.412604072	1.184975873	1.683958558	0.000116626
AC145207.5	1.770775005	1.368265041	2.291693513	1.41E-05
AC107068.1	2.055068325	1.397654586	3.02170927	0.00025018
GSEC	1.714551784	1.327797167	2.213958496	3.57E-05
POLH-AS1	2.534038272	1.687321209	3.805647632	7.42E-06
SNHG10	1.296527829	1.127908092	1.49035584	0.000258992
NCK1-DT	1.531113116	1.226951869	1.910675907	0.00016318
AC012073.1	1.412237124	1.232102684	1.618707369	7.12E-07
ZNF337-AS1	2.763138839	1.56591286	4.875709524	0.00045192
LNCSRLR	2.259863706	1.597454313	3.196951507	4.09E-06
SREBF2-AS1	1.458357305	1.169835935	1.818037867	0.000794755
KDM4A-AS1	2.851826234	1.897616105	4.285857846	4.60E-07
AL355574.1	1.290173713	1.120599813	1.485408251	0.000394545
AL928654.1	1.90931137	1.323536362	2.754340576	0.000541679
HMGN3-AS1	2.147993323	1.435868935	3.213298377	0.000198875
AL513320.1	2.202652357	1.385348142	3.502135859	0.000844866
BBOX1-AS1	1.262219233	1.103218856	1.44413539	0.000699071
MED8-AS1	2.448164016	1.526403927	3.926553739	0.000203584
AC073611.1	1.517525352	1.207304136	1.907459044	0.000350854
MKLN1-AS	3.377528917	2.285090293	4.992232308	1.03E-09
AC018690.1	2.103741938	1.43468071	3.084818881	0.000140019
AP003469.2	1.325450989	1.134717734	1.548244352	0.000379007
LINC01224	1.868911103	1.411004512	2.475419945	1.29E-05
MIR210HG	1.140747196	1.067701893	1.21878979	9.61E-05
AL590705.3	2.005662331	1.342574581	2.996244263	0.000677707
NRAV	1.234701097	1.129596699	1.349585034	3.41E-06
AC016394.2	1.608896054	1.262655249	2.050081773	0.000119959
ZFPM2-AS1	1.096086666	1.05616683	1.137515348	1.25E-06
ZEB1-AS1	1.461886236	1.179401055	1.81203108	0.000527989
FOXD2-AS1	1.154025653	1.062415655	1.253535	0.000687105
TMCC1-AS1	2.640724794	1.846203778	3.777171036	1.05E-07
AC145207.8	1.17867232	1.08354978	1.282145465	0.000128664
AL603839.3	1.3598251	1.140569295	1.62122925	0.000612307

SNHG4	1.482148907	1.283917591	1.71098628	7.81E-08
AC068506.1	1.200833516	1.078434658	1.337124251	0.000848032
AC006504.7	1.392192551	1.157676936	1.674215006	0.000438679
ZBTB11-AS1	2.170738913	1.386223363	3.399241099	0.000706112
AC074117.1	1.47801181	1.206642514	1.810411025	0.000160097
AC026401.3	1.086114688	1.038429719	1.135989364	0.000310758
SNHG3	1.068790137	1.039154618	1.099270828	3.54E-06
AC009005.1	1.164561793	1.072593828	1.264415414	0.000283843
LINC01011	1.996524204	1.325989179	3.006139841	0.000928733
AC125437.1	2.512656059	1.577460487	4.002281212	0.000104863
AL031985.3	1.879214584	1.546887511	2.282937465	2.10E-10
SNHG21	1.725178509	1.263559183	2.355442411	0.000598367
PRRT3-AS1	1.095343996	1.041897754	1.15153187	0.000359635
AC034229.4	1.98718165	1.414862404	2.791007024	7.42E-05
AC023157.2	1.286142305	1.156216332	1.430668279	3.63E-06
LINC01138	1.399793842	1.203228957	1.62847045	1.32E-05

Multivariate Cox analysis					
LncRNA	coef	HR	HR.95L	HR.95H	pvalue（overall survival）
AC145207.5	0.296846923	1.345609302	0.986465021	1.835507954	0.040939138
POLH-AS1	0.668675804	1.95165124	1.117464075	3.408559297	0.01875643
AL928654.1	0.45711117	1.579504468	1.047045083	2.382738247	0.029322787
MKLN1-AS	0.818755303	2.267675511	1.408159271	3.651825706	0.000757309
AL031985.3	0.258485931	1.294967931	0.990534222	1.692967192	0.038703498
PRRT3-AS1	0.073707757	1.076492162	1.016012954	1.140571456	0.012473757
