# Supplementary material for: Inflammatory Response-Related Long Non-Coding RNA Signature Predicts the Prognosis of Hepatocellular Carcinoma
Source: J Oncol. 2022 Mar 17;2022:9917244. doi: 10.1155/2022/9917244 (PMC8947866; doi:10.1155/2022/9917244)
Supplement: Supplementary Materials — Supplementary tables: Supplementary Table 1. Identified inflammatory response-related genes from the Molecular Signatures Database. Supplementary Table 2. The inflammatory response-related DEGs between HCC and noncancerous liver tissues. Supplementary Table 3. The results of univariate Cox regression, LASSO regression, and multivariate Cox regression analysis. Supplementary Table 4. The net benefit of risk score model in DCA analysis. Supplementary Table 5. The results of gene set enrichment analysis. Supplementary Table 6. The immune responses in low- and high-risk groups. [file 9917244.f1.zip › 9917244.f1/Table S4.pdf]

Area under Decision Curve (predict time=1 year)	
RiskScore	1.74E-02
T.Stage	1.45E-02
Stage	1.39E-02
All	4.40E-03
M.Stage	8.30E-04
N.Stage	2.69E-04
Gender	2.17E-04
Age	2.97E-06
Grade	1.28E-06

Area under Decision Curve (predict time=3 year)	
RiskScore	5.14E-02
Stage	3.96E-02
T.Stage	3.84E-02
All	1.22E-02
M.Stage	6.41E-03
N.Stage	2.97E-03
Gender	1.15E-03
Grade	2.41E-05
Age	4.23E-07

Area under Decision Curve (predict time=5 year)	
RiskScore	0.064926913
Stage	0.054058118
T.Stage	0.051966803
All	0.017468094
M.Stage	0.006791451
N.Stage	0.003418889
Gender	0.002411714
Grade	0.000259206
Age	2.24E-05

Net Benifit(predict time=1 year)					
Thresholds	TPR	FPR	Net Benift	time	model
0.1174551	0.191157955	0.808842045	8.35E-02	1	RiskScore
0.1175171	0.191204603	0.804466392	8.41E-02	1	RiskScore
0.1175567	0.191251724	0.800090268	8.47E-02	1	RiskScore
0.1177723	0.191299323	0.795713664	8.51E-02	1	RiskScore
0.1180152	0.191347408	0.791336575	8.55E-02	1	RiskScore
0.1190978	0.191395987	0.786958992	8.50E-02	1	RiskScore
0.1192896	0.191445067	0.782580907	8.54E-02	1	RiskScore
0.1196675	0.191494656	0.778202313	8.57E-02	1	RiskScore
0.1197858	0.191544763	0.773823203	8.62E-02	1	RiskScore
0.1204266	0.191064723	0.769974238	8.56E-02	1	RiskScore
0.1204916	0.191112786	0.76559717	8.62E-02	1	RiskScore
0.1205263	0.191161356	0.761219597	8.68E-02	1	RiskScore
0.1206257	0.191210439	0.756841509	8.74E-02	1	RiskScore

0.1208679	0.191260045	0.752462899	8.78E-02	1	RiskScore
0.1210279	0.191310182	0.748083758	8.83E-02	1	RiskScore
0.121139	0.191360858	0.743704077	8.89E-02	1	RiskScore
0.1213953	0.191412082	0.739323849	8.93E-02	1	RiskScore
0.1215157	0.191463864	0.734943063	8.98E-02	1	RiskScore
0.1224095	0.191516212	0.730561711	8.96E-02	1	RiskScore
0.1224303	0.191569135	0.726179782	9.03E-02	1	RiskScore
0.1228399	0.191622645	0.721797269	9.05E-02	1	RiskScore
0.1230731	0.191676749	0.71741416	9.10E-02	1	RiskScore
0.123507	0.19173146	0.713030445	9.13E-02	1	RiskScore
0.1236021	0.191786785	0.708646115	9.18E-02	1	RiskScore
0.1236515	0.191842738	0.704261159	9.25E-02	1	RiskScore
0.1237408	0.191899327	0.699875565	9.31E-02	1	RiskScore
0.1237924	0.191956564	0.695489323	9.37E-02	1	RiskScore
0.1238494	0.192014461	0.691102422	9.43E-02	1	RiskScore
0.1238637	0.192073029	0.68671485	9.50E-02	1	RiskScore
0.1239591	0.192132279	0.682326595	9.56E-02	1	RiskScore
0.1240149	0.192192225	0.677937645	9.62E-02	1	RiskScore
0.1240284	0.192150682	0.673650183	9.68E-02	1	RiskScore
0.1242893	0.192211261	0.6692606	9.72E-02	1	RiskScore
0.1243491	0.187792127	0.66935073	9.27E-02	1	RiskScore
0.1244431	0.187853081	0.664960772	9.33E-02	1	RiskScore
0.1245854	0.187914767	0.660570082	9.39E-02	1	RiskScore
0.124627	0.187977197	0.656178647	9.46E-02	1	RiskScore
0.1246601	0.188040386	0.651786454	9.52E-02	1	RiskScore
0.1246838	0.183676016	0.65182182	9.08E-02	1	RiskScore
0.1248687	0.183739847	0.647428984	9.14E-02	1	RiskScore
0.1251189	0.18327733	0.643562496	9.12E-02	1	RiskScore
0.1252009	0.18333898	0.639171843	9.19E-02	1	RiskScore
0.1252218	0.182795692	0.635386126	9.18E-02	1	RiskScore
0.1252755	0.178000806	0.635852008	8.69E-02	1	RiskScore
0.125601	0.178056878	0.631466932	8.74E-02	1	RiskScore
0.1259371	0.178113645	0.627081161	8.78E-02	1	RiskScore
0.1260257	0.17817112	0.622694681	8.84E-02	1	RiskScore
0.1261139	0.178229317	0.61830748	8.90E-02	1	RiskScore
0.126446	0.178288249	0.613919543	8.94E-02	1	RiskScore
0.126651	0.178347931	0.609530857	9.00E-02	1	RiskScore
0.1266758	0.178408377	0.605141407	9.06E-02	1	RiskScore
0.1267089	0.178469602	0.600751178	9.13E-02	1	RiskScore
0.1272035	0.178531621	0.596360154	9.16E-02	1	RiskScore
0.1272522	0.17859445	0.59196832	9.23E-02	1	RiskScore
0.1273927	0.178658105	0.587575661	9.29E-02	1	RiskScore
0.1275112	0.174075059	0.587829703	8.82E-02	1	RiskScore
0.1275619	0.174138242	0.583437516	8.88E-02	1	RiskScore
0.1275677	0.174202271	0.579044482	8.95E-02	1	RiskScore
0.1281066	0.169583593	0.579334156	8.45E-02	1	RiskScore
0.1281126	0.169646905	0.574941839	8.52E-02	1	RiskScore
0.1287596	0.169711083	0.570548658	8.54E-02	1	RiskScore

0.1288451	0.169776143	0.566154593	8.60E-02	1	RiskScore
0.1288714	0.169842105	0.561759627	8.67E-02	1	RiskScore
0.1293655	0.169908988	0.557363739	8.71E-02	1	RiskScore
0.1298241	0.169976811	0.552966912	8.75E-02	1	RiskScore
0.129917	0.165310352	0.553304367	8.27E-02	1	RiskScore
0.1299208	0.165377134	0.54890858	8.34E-02	1	RiskScore
0.1303537	0.165444874	0.544511836	8.38E-02	1	RiskScore
0.130783	0.165513593	0.540114113	8.42E-02	1	RiskScore
0.130789	0.165583312	0.535715389	8.50E-02	1	RiskScore
0.1308927	0.165654054	0.531315643	8.56E-02	1	RiskScore
0.1311543	0.165725842	0.52691485	8.62E-02	1	RiskScore
0.131293	0.1657987	0.522512989	8.68E-02	1	RiskScore
0.1314538	0.165872651	0.518110033	8.75E-02	1	RiskScore
0.1322258	0.16594772	0.513705959	8.77E-02	1	RiskScore
0.132235	0.166023934	0.509300741	8.84E-02	1	RiskScore
0.1324747	0.16610132	0.504894351	8.90E-02	1	RiskScore
0.1328668	0.166179904	0.500486762	8.95E-02	1	RiskScore
0.1330395	0.166259716	0.496077947	9.01E-02	1	RiskScore
0.1331343	0.166340783	0.491667875	9.08E-02	1	RiskScore
0.1331434	0.165313043	0.48836661	9.03E-02	1	RiskScore
0.1334561	0.165387904	0.483962745	9.09E-02	1	RiskScore
0.1335953	0.165463971	0.479557674	9.15E-02	1	RiskScore
0.1336072	0.165541276	0.475151365	9.23E-02	1	RiskScore
0.1338333	0.165619848	0.470743788	9.29E-02	1	RiskScore
0.1338592	0.16569972	0.466334912	9.36E-02	1	RiskScore
0.1340548	0.165780924	0.461924704	9.43E-02	1	RiskScore
0.1340962	0.165863494	0.457513129	9.50E-02	1	RiskScore
0.1341858	0.165947466	0.453100153	9.57E-02	1	RiskScore
0.1342218	0.166032877	0.448685738	9.65E-02	1	RiskScore
0.1345603	0.165915182	0.444474429	9.68E-02	1	RiskScore
0.1346902	0.166001197	0.440059409	9.75E-02	1	RiskScore
0.1347318	0.166088713	0.435642889	9.83E-02	1	RiskScore
0.1349608	0.166177769	0.431224828	9.89E-02	1	RiskScore
0.1352009	0.166268407	0.426805186	9.95E-02	1	RiskScore
0.1353455	0.16636067	0.422383919	1.00E-01	1	RiskScore
0.1355592	0.166454602	0.417960982	1.01E-01	1	RiskScore
0.1356091	0.166550251	0.413536329	1.02E-01	1	RiskScore
0.1356344	0.166647663	0.409109912	1.02E-01	1	RiskScore
0.1365578	0.16674689	0.404681682	1.03E-01	1	RiskScore
0.1367252	0.166847982	0.400251585	1.03E-01	1	RiskScore
0.1375794	0.166950994	0.395819569	1.04E-01	1	RiskScore
0.1377879	0.167055982	0.391385577	1.05E-01	1	RiskScore
0.1384448	0.162678432	0.391434122	9.98E-02	1	RiskScore
0.1384479	0.162785239	0.386998311	1.01E-01	1	RiskScore
0.1384601	0.161689994	0.383764551	1.00E-01	1	RiskScore
0.1384806	0.161788821	0.37933672	1.01E-01	1	RiskScore
0.1385358	0.161889617	0.37490692	1.02E-01	1	RiskScore
0.1389959	0.15726515	0.375202383	9.67E-02	1	RiskScore

0.1391629	0.156547993	0.371590535	9.65E-02	1	RiskScore
0.1397022	0.156641068	0.367168456	9.70E-02	1	RiskScore
0.1402737	0.156736041	0.362744478	9.76E-02	1	RiskScore
0.1403094	0.156832973	0.358318542	9.84E-02	1	RiskScore
0.140614	0.156681536	0.354140975	9.87E-02	1	RiskScore
0.1406168	0.156778992	0.349714515	9.96E-02	1	RiskScore
0.1409937	0.156878499	0.345286003	1.00E-01	1	RiskScore
0.1410181	0.156980124	0.340855374	1.01E-01	1	RiskScore
0.1412668	0.152597713	0.34090878	9.65E-02	1	RiskScore
0.1412717	0.152701274	0.336476215	9.73E-02	1	RiskScore
0.1413506	0.152807094	0.332041391	9.81E-02	1	RiskScore
0.1416197	0.152915249	0.327604232	9.89E-02	1	RiskScore
0.1420243	0.153025818	0.323164658	9.95E-02	1	RiskScore
0.1420726	0.153138885	0.318722587	1.00E-01	1	RiskScore
0.1432529	0.153254537	0.314277931	1.01E-01	1	RiskScore
0.1433123	0.153372864	0.309830599	1.02E-01	1	RiskScore
0.1443586	0.153493962	0.305380497	1.02E-01	1	RiskScore
0.1443935	0.153617932	0.300927522	1.03E-01	1	RiskScore
0.1448429	0.153744879	0.296471572	1.04E-01	1	RiskScore
0.1448945	0.149064248	0.296823198	9.88E-02	1	RiskScore
0.145008	0.144613887	0.296944555	9.43E-02	1	RiskScore
0.1451322	0.144740321	0.292489117	9.51E-02	1	RiskScore
0.1456232	0.144869917	0.288030516	9.58E-02	1	RiskScore
0.145922	0.145002798	0.283568631	9.66E-02	1	RiskScore
0.1465388	0.145139092	0.279103332	9.72E-02	1	RiskScore
0.1472713	0.145278935	0.274634485	9.78E-02	1	RiskScore
0.1475453	0.144300657	0.271283759	9.73E-02	1	RiskScore
0.1479219	0.142859621	0.26839579	9.63E-02	1	RiskScore
0.1493054	0.142972861	0.263953546	9.66E-02	1	RiskScore
0.1497837	0.14308916	0.259508242	9.74E-02	1	RiskScore
0.1509127	0.143208649	0.25505975	9.79E-02	1	RiskScore
0.151405	0.143331461	0.250607932	9.86E-02	1	RiskScore
0.1517057	0.143457742	0.246152647	9.94E-02	1	RiskScore
0.1521114	0.143587643	0.241693742	1.00E-01	1	RiskScore
0.1521493	0.143721326	0.237231055	1.01E-01	1	RiskScore
0.1526215	0.143858962	0.232764415	1.02E-01	1	RiskScore
0.1528792	0.139139067	0.233155305	9.71E-02	1	RiskScore
0.1533257	0.13927666	0.228688708	9.79E-02	1	RiskScore
0.1533727	0.139418506	0.224217857	9.88E-02	1	RiskScore
0.1534597	0.139564811	0.219742549	9.97E-02	1	RiskScore
0.1536249	0.139715793	0.215262562	1.01E-01	1	RiskScore
0.1548138	0.139871686	0.210777664	1.01E-01	1	RiskScore
0.1572974	0.138178231	0.208142115	9.93E-02	1	RiskScore
0.1578208	0.138315816	0.203675526	1.00E-01	1	RiskScore
0.157842	0.138458159	0.199204179	1.01E-01	1	RiskScore
0.1584097	0.138605519	0.194727815	1.02E-01	1	RiskScore
0.1600018	0.134205218	0.194799111	9.71E-02	1	RiskScore
0.1615942	0.134357586	0.190317738	9.77E-02	1	RiskScore

0.1639793	0.129618586	0.190727734	9.22E-02	1	RiskScore
0.1640688	0.128696407	0.187320909	9.19E-02	1	RiskScore
0.1641911	0.128830517	0.182857795	9.29E-02	1	RiskScore
0.1679791	0.128969666	0.178389641	9.30E-02	1	RiskScore
0.1689961	0.124275789	0.178754514	8.79E-02	1	RiskScore
0.1706492	0.124415443	0.174285855	8.86E-02	1	RiskScore
0.1741342	0.124560653	0.169811641	8.88E-02	1	RiskScore
0.1750867	0.120193812	0.169849478	8.41E-02	1	RiskScore
0.175153	0.119050902	0.166663384	8.37E-02	1	RiskScore
0.1751531	0.119177755	0.162207526	8.47E-02	1	RiskScore
0.1755223	0.11930985	0.157746427	8.57E-02	1	RiskScore
0.1782359	0.119447531	0.153279741	8.62E-02	1	RiskScore
0.1784742	0.118720004	0.149678265	8.62E-02	1	RiskScore
0.1786401	0.118844716	0.145224548	8.73E-02	1	RiskScore
0.1792124	0.11897476	0.1407655	8.82E-02	1	RiskScore
0.1802676	0.1144876	0.140923655	8.35E-02	1	RiskScore
0.1825603	0.114622076	0.136460175	8.41E-02	1	RiskScore
0.1827529	0.114762674	0.131990573	8.52E-02	1	RiskScore
0.1832845	0.110005441	0.132418802	8.03E-02	1	RiskScore
0.183433	0.110142823	0.127952416	8.14E-02	1	RiskScore
0.1847056	0.110286724	0.12347951	8.23E-02	1	RiskScore
0.1876779	0.110437636	0.118999594	8.29E-02	1	RiskScore
0.1896526	0.1105961	0.114512125	8.38E-02	1	RiskScore
0.1926022	0.110762721	0.1100165	8.45E-02	1	RiskScore
0.1930635	0.106389692	0.110060524	8.01E-02	1	RiskScore
0.1946963	0.106565198	0.105556014	8.10E-02	1	RiskScore
0.1991878	0.10214043	0.105651778	7.59E-02	1	RiskScore
0.2027606	0.097711353	0.105751851	7.08E-02	1	RiskScore
0.204756	0.097896499	0.1012377	7.18E-02	1	RiskScore
0.20936	0.093462667	0.101342528	6.66E-02	1	RiskScore
0.2098129	0.089023776	0.101452414	6.21E-02	1	RiskScore
0.2108325	0.084373017	0.101774169	5.72E-02	1	RiskScore
0.2133745	0.084564269	0.097253913	5.82E-02	1	RiskScore
0.2138887	0.084767516	0.092721661	5.95E-02	1	RiskScore
0.2171761	0.084983958	0.088176215	6.05E-02	1	RiskScore
0.2193671	0.085214958	0.08361621	6.17E-02	1	RiskScore
0.2195255	0.085462085	0.079040079	6.32E-02	1	RiskScore
0.220542	0.085727146	0.074446014	6.47E-02	1	RiskScore
0.2215812	0.084037927	0.071806229	6.36E-02	1	RiskScore
0.2277044	0.079650258	0.071864894	5.85E-02	1	RiskScore
0.2287748	0.079865211	0.067320937	5.99E-02	1	RiskScore
0.2290114	0.080096555	0.062760588	6.15E-02	1	RiskScore
0.2296988	0.08034632	0.058181818	6.30E-02	1	RiskScore
0.233278	0.078668458	0.055530676	6.18E-02	1	RiskScore
0.2359024	0.078849722	0.051020408	6.31E-02	1	RiskScore
0.2428996	0.074394741	0.051146384	5.80E-02	1	RiskScore
0.24356	0.06993007	0.051282051	5.34E-02	1	RiskScore
0.251763	0.065454545	0.051428571	4.82E-02	1	RiskScore

0.2521233	0.065656566	0.046897547	4.98E-02	1	RiskScore
0.2542157	0.065876153	0.042348955	5.14E-02	1	RiskScore
0.2580901	0.066115702	0.037780401	5.30E-02	1	RiskScore
0.261423	0.061636776	0.037930324	4.82E-02	1	RiskScore
0.2668267	0.058956916	0.036281179	4.58E-02	1	RiskScore
0.2812142	0.054545455	0.036363636	4.03E-02	1	RiskScore
0.2902194	0.050125313	0.036454773	3.52E-02	1	RiskScore
0.2941524	0.05026455	0.031986532	3.69E-02	1	RiskScore
0.3029261	0.050420168	0.02750191	3.85E-02	1	RiskScore
0.3060872	0.045995671	0.027597403	3.38E-02	1	RiskScore
0.3092529	0.041558442	0.027705628	2.92E-02	1	RiskScore
0.3549267	0.037105751	0.027829314	2.18E-02	1	RiskScore
0.3853662	0.037296037	0.023310023	2.27E-02	1	RiskScore
0.403764	0.034632035	0.021645022	2.00E-02	1	RiskScore
0.4066268	0.034632035	0.017316017	2.28E-02	1	RiskScore
0.4151107	0.034632035	0.012987013	2.54E-02	1	RiskScore
0.4629593	0.03030303	0.012987013	1.91E-02	1	RiskScore
0.4692358	0.025974026	0.012987013	1.45E-02	1	RiskScore
0.5241641	0.021645022	0.012987013	7.34E-03	1	RiskScore
0.5295047	0.017316017	0.012987013	2.70E-03	1	RiskScore
0.551684	0.012987013	0.012987013	-2.99E-03	1	RiskScore
0.5619494	0.008658009	0.012987013	-8.00E-03	1	RiskScore
0.5918713	0.004329004	0.012987013	-1.45E-02	1	RiskScore
0.7094639	0.004329004	0.008658009	-1.68E-02	1	RiskScore
0.9289219	0.004329004	0.004329004	-5.22E-02	1	RiskScore
0.9757418	0.004329004	0	4.33E-03	1	RiskScore
0.1901865	0.191157955	0.808842045	1.20E-03	1	Age
0.192052	0.059799325	0.243230978	1.98E-03	1	Age
0.1768201	0.191157955	0.808842045	1.74E-02	1	Gender
0.2212035	0.058364473	0.253323838	-1.36E-02	1	Gender
0.1876487	0.191157955	0.808842045	4.32E-03	1	Grade
0.1898527	0.154232817	0.720226058	-1.45E-02	1	Grade
0.1920793	0.083777818	0.353451619	-2.54E-04	1	Grade
0.1943289	0.008658009	0.034632035	3.05E-04	1	Grade
0.1037935	0.191157955	0.808842045	9.75E-02	1	Stage
0.1860792	0.146246182	0.364576329	6.29E-02	1	Stage
0.3208	0.110396123	0.188305176	2.15E-02	1	Stage
0.5165521	0.005772006	0.011544012	-6.56E-03	1	Stage
0.1056181	0.191157955	0.808842045	9.56E-02	1	T.Stage
0.1835074	0.145127656	0.357036847	6.49E-02	1	T.Stage
0.3080405	0.108974947	0.176739339	3.03E-02	1	T.Stage
0.4876794	0.018552876	0.024737168	-4.99E-03	1	T.Stage
0.1881333	0.191157955	0.808842045	3.73E-03	1	N.Stage
0.3506363	0.005772006	0.011544012	-4.61E-04	1	N.Stage
0.1845822	0.191157955	0.808842045	8.06E-03	1	M.Stage
0.5513041	0.004329004	0.008658009	-6.31E-03	1	M.Stage
0	0.191157955	0.808842045	1.91E-01	1	All
0.1037935	0.191157955	0.808842045	9.75E-02	1	All

0.1056181	0.191157955	0.808842045	9.56E-02	1	All
0.1174551	0.191157955	0.808842045	8.35E-02	1	All
0.1175171	0.191157955	0.808842045	8.34E-02	1	All
0.1175567	0.191157955	0.808842045	8.34E-02	1	All
0.1177723	0.191157955	0.808842045	8.32E-02	1	All
0.1180152	0.191157955	0.808842045	8.29E-02	1	All
0.1190978	0.191157955	0.808842045	8.18E-02	1	All
0.1192896	0.191157955	0.808842045	8.16E-02	1	All
0.1196675	0.191157955	0.808842045	8.12E-02	1	All
0.1197858	0.191157955	0.808842045	8.11E-02	1	All
0.1204266	0.191157955	0.808842045	8.04E-02	1	All
0.1204916	0.191157955	0.808842045	8.03E-02	1	All
0.1205263	0.191157955	0.808842045	8.03E-02	1	All
0.1206257	0.191157955	0.808842045	8.02E-02	1	All
0.1208679	0.191157955	0.808842045	8.00E-02	1	All
0.1210279	0.191157955	0.808842045	7.98E-02	1	All
0.121139	0.191157955	0.808842045	7.97E-02	1	All
0.1213953	0.191157955	0.808842045	7.94E-02	1	All
0.1215157	0.191157955	0.808842045	7.93E-02	1	All
0.1224095	0.191157955	0.808842045	7.83E-02	1	All
0.1224303	0.191157955	0.808842045	7.83E-02	1	All
0.1228399	0.191157955	0.808842045	7.79E-02	1	All
0.1230731	0.191157955	0.808842045	7.76E-02	1	All
0.123507	0.191157955	0.808842045	7.72E-02	1	All
0.1236021	0.191157955	0.808842045	7.71E-02	1	All
0.1236515	0.191157955	0.808842045	7.70E-02	1	All
0.1237408	0.191157955	0.808842045	7.69E-02	1	All
0.1237924	0.191157955	0.808842045	7.69E-02	1	All
0.1238494	0.191157955	0.808842045	7.68E-02	1	All
0.1238637	0.191157955	0.808842045	7.68E-02	1	All
0.1239591	0.191157955	0.808842045	7.67E-02	1	All
0.1240149	0.191157955	0.808842045	7.66E-02	1	All
0.1240284	0.191157955	0.808842045	7.66E-02	1	All
0.1242893	0.191157955	0.808842045	7.64E-02	1	All
0.1243491	0.191157955	0.808842045	7.63E-02	1	All
0.1244431	0.191157955	0.808842045	7.62E-02	1	All
0.1245854	0.191157955	0.808842045	7.60E-02	1	All
0.124627	0.191157955	0.808842045	7.60E-02	1	All
0.1246601	0.191157955	0.808842045	7.60E-02	1	All
0.1246838	0.191157955	0.808842045	7.59E-02	1	All
0.1248687	0.191157955	0.808842045	7.57E-02	1	All
0.1251189	0.191157955	0.808842045	7.55E-02	1	All
0.1252009	0.191157955	0.808842045	7.54E-02	1	All
0.1252218	0.191157955	0.808842045	7.54E-02	1	All
0.1252755	0.191157955	0.808842045	7.53E-02	1	All
0.125601	0.191157955	0.808842045	7.50E-02	1	All
0.1259371	0.191157955	0.808842045	7.46E-02	1	All
0.1260257	0.191157955	0.808842045	7.45E-02	1	All

0.1261139	0.191157955	0.808842045	7.44E-02	1	All
0.126446	0.191157955	0.808842045	7.41E-02	1	All
0.126651	0.191157955	0.808842045	7.39E-02	1	All
0.1266758	0.191157955	0.808842045	7.38E-02	1	All
0.1267089	0.191157955	0.808842045	7.38E-02	1	All
0.1272035	0.191157955	0.808842045	7.33E-02	1	All
0.1272522	0.191157955	0.808842045	7.32E-02	1	All
0.1273927	0.191157955	0.808842045	7.31E-02	1	All
0.1275112	0.191157955	0.808842045	7.29E-02	1	All
0.1275619	0.191157955	0.808842045	7.29E-02	1	All
0.1275677	0.191157955	0.808842045	7.29E-02	1	All
0.1281066	0.191157955	0.808842045	7.23E-02	1	All
0.1281126	0.191157955	0.808842045	7.23E-02	1	All
0.1287596	0.191157955	0.808842045	7.16E-02	1	All
0.1288451	0.191157955	0.808842045	7.15E-02	1	All
0.1288714	0.191157955	0.808842045	7.15E-02	1	All
0.1293655	0.191157955	0.808842045	7.10E-02	1	All
0.1298241	0.191157955	0.808842045	7.05E-02	1	All
0.129917	0.191157955	0.808842045	7.04E-02	1	All
0.1299208	0.191157955	0.808842045	7.04E-02	1	All
0.1303537	0.191157955	0.808842045	6.99E-02	1	All
0.130783	0.191157955	0.808842045	6.95E-02	1	All
0.130789	0.191157955	0.808842045	6.95E-02	1	All
0.1308927	0.191157955	0.808842045	6.93E-02	1	All
0.1311543	0.191157955	0.808842045	6.91E-02	1	All
0.131293	0.191157955	0.808842045	6.89E-02	1	All
0.1314538	0.191157955	0.808842045	6.87E-02	1	All
0.1322258	0.191157955	0.808842045	6.79E-02	1	All
0.132235	0.191157955	0.808842045	6.79E-02	1	All
0.1324747	0.191157955	0.808842045	6.76E-02	1	All
0.1328668	0.191157955	0.808842045	6.72E-02	1	All
0.1330395	0.191157955	0.808842045	6.70E-02	1	All
0.1331343	0.191157955	0.808842045	6.69E-02	1	All
0.1331434	0.191157955	0.808842045	6.69E-02	1	All
0.1334561	0.191157955	0.808842045	6.66E-02	1	All
0.1335953	0.191157955	0.808842045	6.64E-02	1	All
0.1336072	0.191157955	0.808842045	6.64E-02	1	All
0.1338333	0.191157955	0.808842045	6.62E-02	1	All
0.1338592	0.191157955	0.808842045	6.62E-02	1	All
0.1340548	0.191157955	0.808842045	6.59E-02	1	All
0.1340962	0.191157955	0.808842045	6.59E-02	1	All
0.1341858	0.191157955	0.808842045	6.58E-02	1	All
0.1342218	0.191157955	0.808842045	6.58E-02	1	All
0.1345603	0.191157955	0.808842045	6.54E-02	1	All
0.1346902	0.191157955	0.808842045	6.53E-02	1	All
0.1347318	0.191157955	0.808842045	6.52E-02	1	All
0.1349608	0.191157955	0.808842045	6.50E-02	1	All
0.1352009	0.191157955	0.808842045	6.47E-02	1	All

0.1353455	0.191157955	0.808842045	6.45E-02	1	All
0.1355592	0.191157955	0.808842045	6.43E-02	1	All
0.1356091	0.191157955	0.808842045	6.43E-02	1	All
0.1356344	0.191157955	0.808842045	6.42E-02	1	All
0.1365578	0.191157955	0.808842045	6.32E-02	1	All
0.1367252	0.191157955	0.808842045	6.31E-02	1	All
0.1375794	0.191157955	0.808842045	6.21E-02	1	All
0.1377879	0.191157955	0.808842045	6.19E-02	1	All
0.1384448	0.191157955	0.808842045	6.12E-02	1	All
0.1384479	0.191157955	0.808842045	6.12E-02	1	All
0.1384601	0.191157955	0.808842045	6.12E-02	1	All
0.1384806	0.191157955	0.808842045	6.11E-02	1	All
0.1385358	0.191157955	0.808842045	6.11E-02	1	All
0.1389959	0.191157955	0.808842045	6.06E-02	1	All
0.1391629	0.191157955	0.808842045	6.04E-02	1	All
0.1397022	0.191157955	0.808842045	5.98E-02	1	All
0.1402737	0.191157955	0.808842045	5.92E-02	1	All
0.1403094	0.191157955	0.808842045	5.91E-02	1	All
0.140614	0.191157955	0.808842045	5.88E-02	1	All
0.1406168	0.191157955	0.808842045	5.88E-02	1	All
0.1409937	0.191157955	0.808842045	5.84E-02	1	All
0.1410181	0.191157955	0.808842045	5.84E-02	1	All
0.1412668	0.191157955	0.808842045	5.81E-02	1	All
0.1412717	0.191157955	0.808842045	5.81E-02	1	All
0.1413506	0.191157955	0.808842045	5.80E-02	1	All
0.1416197	0.191157955	0.808842045	5.77E-02	1	All
0.1420243	0.191157955	0.808842045	5.73E-02	1	All
0.1420726	0.191157955	0.808842045	5.72E-02	1	All
0.1432529	0.191157955	0.808842045	5.59E-02	1	All
0.1433123	0.191157955	0.808842045	5.58E-02	1	All
0.1443586	0.191157955	0.808842045	5.47E-02	1	All
0.1443935	0.191157955	0.808842045	5.47E-02	1	All
0.1448429	0.191157955	0.808842045	5.42E-02	1	All
0.1448945	0.191157955	0.808842045	5.41E-02	1	All
0.145008	0.191157955	0.808842045	5.40E-02	1	All
0.1451322	0.191157955	0.808842045	5.38E-02	1	All
0.1456232	0.191157955	0.808842045	5.33E-02	1	All
0.145922	0.191157955	0.808842045	5.30E-02	1	All
0.1465388	0.191157955	0.808842045	5.23E-02	1	All
0.1472713	0.191157955	0.808842045	5.15E-02	1	All
0.1475453	0.191157955	0.808842045	5.12E-02	1	All
0.1479219	0.191157955	0.808842045	5.07E-02	1	All
0.1493054	0.191157955	0.808842045	4.92E-02	1	All
0.1497837	0.191157955	0.808842045	4.87E-02	1	All
0.1509127	0.191157955	0.808842045	4.74E-02	1	All
0.151405	0.191157955	0.808842045	4.68E-02	1	All
0.1517057	0.191157955	0.808842045	4.65E-02	1	All
0.1521114	0.191157955	0.808842045	4.61E-02	1	All

0.1521493	0.191157955	0.808842045	4.60E-02	1	All
0.1526215	0.191157955	0.808842045	4.55E-02	1	All
0.1528792	0.191157955	0.808842045	4.52E-02	1	All
0.1533257	0.191157955	0.808842045	4.47E-02	1	All
0.1533727	0.191157955	0.808842045	4.46E-02	1	All
0.1534597	0.191157955	0.808842045	4.45E-02	1	All
0.1536249	0.191157955	0.808842045	4.43E-02	1	All
0.1548138	0.191157955	0.808842045	4.30E-02	1	All
0.1572974	0.191157955	0.808842045	4.02E-02	1	All
0.1578208	0.191157955	0.808842045	3.96E-02	1	All
0.157842	0.191157955	0.808842045	3.96E-02	1	All
0.1584097	0.191157955	0.808842045	3.89E-02	1	All
0.1600018	0.191157955	0.808842045	3.71E-02	1	All
0.1615942	0.191157955	0.808842045	3.53E-02	1	All
0.1639793	0.191157955	0.808842045	3.25E-02	1	All
0.1640688	0.191157955	0.808842045	3.24E-02	1	All
0.1641911	0.191157955	0.808842045	3.23E-02	1	All
0.1679791	0.191157955	0.808842045	2.79E-02	1	All
0.1689961	0.191157955	0.808842045	2.67E-02	1	All
0.1706492	0.191157955	0.808842045	2.47E-02	1	All
0.1741342	0.191157955	0.808842045	2.06E-02	1	All
0.1750867	0.191157955	0.808842045	1.95E-02	1	All
0.175153	0.191157955	0.808842045	1.94E-02	1	All
0.1751531	0.191157955	0.808842045	1.94E-02	1	All
0.1755223	0.191157955	0.808842045	1.90E-02	1	All
0.1768201	0.191157955	0.808842045	1.74E-02	1	All
0.1782359	0.191157955	0.808842045	1.57E-02	1	All
0.1784742	0.191157955	0.808842045	1.54E-02	1	All
0.1786401	0.191157955	0.808842045	1.52E-02	1	All
0.1792124	0.191157955	0.808842045	1.46E-02	1	All
0.1802676	0.191157955	0.808842045	1.33E-02	1	All
0.1825603	0.191157955	0.808842045	1.05E-02	1	All
0.1827529	0.191157955	0.808842045	1.03E-02	1	All
0.1832845	0.191157955	0.808842045	9.64E-03	1	All
0.183433	0.191157955	0.808842045	9.46E-03	1	All
0.1835074	0.191157955	0.808842045	9.37E-03	1	All
0.1845822	0.191157955	0.808842045	8.06E-03	1	All
0.1847056	0.191157955	0.808842045	7.91E-03	1	All
0.1860792	0.191157955	0.808842045	6.24E-03	1	All
0.1876487	0.191157955	0.808842045	4.32E-03	1	All
0.1876779	0.191157955	0.808842045	4.28E-03	1	All
0.1881333	0.191157955	0.808842045	3.73E-03	1	All
0.1896526	0.191157955	0.808842045	1.86E-03	1	All
0.1898527	0.191157955	0.808842045	1.61E-03	1	All
0.1901865	0.191157955	0.808842045	1.20E-03	1	All
0.192052	0.191157955	0.808842045	-1.11E-03	1	All
0.1920793	0.191157955	0.808842045	-1.14E-03	1	All
0.1926022	0.191157955	0.808842045	-1.79E-03	1	All

0.1930635	0.191157955	0.808842045	-2.36E-03	1	All
0.1943289	0.191157955	0.808842045	-3.94E-03	1	All
0.1946963	0.191157955	0.808842045	-4.39E-03	1	All
0.1991878	0.191157955	0.808842045	-1.00E-02	1	All
0.2027606	0.191157955	0.808842045	-1.46E-02	1	All
0.204756	0.191157955	0.808842045	-1.71E-02	1	All
0.20936	0.191157955	0.808842045	-2.30E-02	1	All
0.2098129	0.191157955	0.808842045	-2.36E-02	1	All
0.2108325	0.191157955	0.808842045	-2.49E-02	1	All
0.2133745	0.191157955	0.808842045	-2.82E-02	1	All
0.2138887	0.191157955	0.808842045	-2.89E-02	1	All
0.2171761	0.191157955	0.808842045	-3.32E-02	1	All
0.2193671	0.191157955	0.808842045	-3.61E-02	1	All
0.2195255	0.191157955	0.808842045	-3.63E-02	1	All
0.220542	0.191157955	0.808842045	-3.77E-02	1	All
0.2212035	0.191157955	0.808842045	-3.86E-02	1	All
0.2215812	0.191157955	0.808842045	-3.91E-02	1	All
0.2277044	0.191157955	0.808842045	-4.73E-02	1	All
0.2287748	0.191157955	0.808842045	-4.88E-02	1	All
0.2290114	0.191157955	0.808842045	-4.91E-02	1	All
0.2296988	0.191157955	0.808842045	-5.00E-02	1	All
0.233278	0.191157955	0.808842045	-5.49E-02	1	All
0.2359024	0.191157955	0.808842045	-5.86E-02	1	All
0.2428996	0.191157955	0.808842045	-6.83E-02	1	All
0.24356	0.191157955	0.808842045	-6.93E-02	1	All
0.251763	0.191157955	0.808842045	-8.10E-02	1	All
0.2521233	0.191157955	0.808842045	-8.15E-02	1	All
0.2542157	0.191157955	0.808842045	-8.46E-02	1	All
0.2580901	0.191157955	0.808842045	-9.02E-02	1	All
0.261423	0.191157955	0.808842045	-9.51E-02	1	All
0.2668267	0.191157955	0.808842045	-1.03E-01	1	All
0.2812142	0.191157955	0.808842045	-1.25E-01	1	All
0.2902194	0.191157955	0.808842045	-1.40E-01	1	All
0.2941524	0.191157955	0.808842045	-1.46E-01	1	All
0.3029261	0.191157955	0.808842045	-1.60E-01	1	All
0.3060872	0.191157955	0.808842045	-1.66E-01	1	All
0.3080405	0.191157955	0.808842045	-1.69E-01	1	All
0.3092529	0.191157955	0.808842045	-1.71E-01	1	All
0.3208	0.191157955	0.808842045	-1.91E-01	1	All
0.3506363	0.191157955	0.808842045	-2.46E-01	1	All
0.3549267	0.191157955	0.808842045	-2.54E-01	1	All
0.3853662	0.191157955	0.808842045	-3.16E-01	1	All
0.403764	0.191157955	0.808842045	-3.57E-01	1	All
0.4066268	0.191157955	0.808842045	-3.63E-01	1	All
0.4151107	0.191157955	0.808842045	-3.83E-01	1	All
0.4629593	0.191157955	0.808842045	-5.06E-01	1	All
0.4692358	0.191157955	0.808842045	-5.24E-01	1	All
0.4876794	0.191157955	0.808842045	-5.79E-01	1	All

0.5165521	0.191157955	0.808842045	-6.73E-01	1	All
0.5241641	0.191157955	0.808842045	-7.00E-01	1	All
0.5295047	0.191157955	0.808842045	-7.19E-01	1	All
0.5513041	0.191157955	0.808842045	-8.03E-01	1	All
0.551684	0.191157955	0.808842045	-8.04E-01	1	All
0.5619494	0.191157955	0.808842045	-8.46E-01	1	All
0.5918713	0.191157955	0.808842045	-9.82E-01	1	All
0.7094639	0.191157955	0.808842045	-1.78E+00	1	All
0.9289219	0.191157955	0.808842045	-1.04E+01	1	All
0.9757418	0.191157955	0.808842045	-3.23E+01	1	All
0	0	0	0.00E+00	1	None
0.1037935	0	0	0.00E+00	1	None
0.1056181	0	0	0.00E+00	1	None
0.1174551	0	0	0.00E+00	1	None
0.1175171	0	0	0.00E+00	1	None
0.1175567	0	0	0.00E+00	1	None
0.1177723	0	0	0.00E+00	1	None
0.1180152	0	0	0.00E+00	1	None
0.1190978	0	0	0.00E+00	1	None
0.1192896	0	0	0.00E+00	1	None
0.1196675	0	0	0.00E+00	1	None
0.1197858	0	0	0.00E+00	1	None
0.1204266	0	0	0.00E+00	1	None
0.1204916	0	0	0.00E+00	1	None
0.1205263	0	0	0.00E+00	1	None
0.1206257	0	0	0.00E+00	1	None
0.1208679	0	0	0.00E+00	1	None
0.1210279	0	0	0.00E+00	1	None
0.121139	0	0	0.00E+00	1	None
0.1213953	0	0	0.00E+00	1	None
0.1215157	0	0	0.00E+00	1	None
0.1224095	0	0	0.00E+00	1	None
0.1224303	0	0	0.00E+00	1	None
0.1228399	0	0	0.00E+00	1	None
0.1230731	0	0	0.00E+00	1	None
0.123507	0	0	0.00E+00	1	None
0.1236021	0	0	0.00E+00	1	None
0.1236515	0	0	0.00E+00	1	None
0.1237408	0	0	0.00E+00	1	None
0.1237924	0	0	0.00E+00	1	None
0.1238494	0	0	0.00E+00	1	None
0.1238637	0	0	0.00E+00	1	None
0.1239591	0	0	0.00E+00	1	None
0.1240149	0	0	0.00E+00	1	None
0.1240284	0	0	0.00E+00	1	None
0.1242893	0	0	0.00E+00	1	None
0.1243491	0	0	0.00E+00	1	None
0.1244431	0	0	0.00E+00	1	None

0.1245854	0	0	0.00E+00	1 None
0.124627	0	0	0.00E+00	1 None
0.1246601	0	0	0.00E+00	1 None
0.1246838	0	0	0.00E+00	1 None
0.1248687	0	0	0.00E+00	1 None
0.1251189	0	0	0.00E+00	1 None
0.1252009	0	0	0.00E+00	1 None
0.1252218	0	0	0.00E+00	1 None
0.1252755	0	0	0.00E+00	1 None
0.125601	0	0	0.00E+00	1 None
0.1259371	0	0	0.00E+00	1 None
0.1260257	0	0	0.00E+00	1 None
0.1261139	0	0	0.00E+00	1 None
0.126446	0	0	0.00E+00	1 None
0.126651	0	0	0.00E+00	1 None
0.1266758	0	0	0.00E+00	1 None
0.1267089	0	0	0.00E+00	1 None
0.1272035	0	0	0.00E+00	1 None
0.1272522	0	0	0.00E+00	1 None
0.1273927	0	0	0.00E+00	1 None
0.1275112	0	0	0.00E+00	1 None
0.1275619	0	0	0.00E+00	1 None
0.1275677	0	0	0.00E+00	1 None
0.1281066	0	0	0.00E+00	1 None
0.1281126	0	0	0.00E+00	1 None
0.1287596	0	0	0.00E+00	1 None
0.1288451	0	0	0.00E+00	1 None
0.1288714	0	0	0.00E+00	1 None
0.1293655	0	0	0.00E+00	1 None
0.1298241	0	0	0.00E+00	1 None
0.129917	0	0	0.00E+00	1 None
0.1299208	0	0	0.00E+00	1 None
0.1303537	0	0	0.00E+00	1 None
0.130783	0	0	0.00E+00	1 None
0.130789	0	0	0.00E+00	1 None
0.1308927	0	0	0.00E+00	1 None
0.1311543	0	0	0.00E+00	1 None
0.131293	0	0	0.00E+00	1 None
0.1314538	0	0	0.00E+00	1 None
0.1322258	0	0	0.00E+00	1 None
0.132235	0	0	0.00E+00	1 None
0.1324747	0	0	0.00E+00	1 None
0.1328668	0	0	0.00E+00	1 None
0.1330395	0	0	0.00E+00	1 None
0.1331343	0	0	0.00E+00	1 None
0.1331434	0	0	0.00E+00	1 None
0.1334561	0	0	0.00E+00	1 None
0.1335953	0	0	0.00E+00	1 None

0.1336072	0	0	0.00E+00	1	None
0.1338333	0	0	0.00E+00	1	None
0.1338592	0	0	0.00E+00	1	None
0.1340548	0	0	0.00E+00	1	None
0.1340962	0	0	0.00E+00	1	None
0.1341858	0	0	0.00E+00	1	None
0.1342218	0	0	0.00E+00	1	None
0.1345603	0	0	0.00E+00	1	None
0.1346902	0	0	0.00E+00	1	None
0.1347318	0	0	0.00E+00	1	None
0.1349608	0	0	0.00E+00	1	None
0.1352009	0	0	0.00E+00	1	None
0.1353455	0	0	0.00E+00	1	None
0.1355592	0	0	0.00E+00	1	None
0.1356091	0	0	0.00E+00	1	None
0.1356344	0	0	0.00E+00	1	None
0.1365578	0	0	0.00E+00	1	None
0.1367252	0	0	0.00E+00	1	None
0.1375794	0	0	0.00E+00	1	None
0.1377879	0	0	0.00E+00	1	None
0.1384448	0	0	0.00E+00	1	None
0.1384479	0	0	0.00E+00	1	None
0.1384601	0	0	0.00E+00	1	None
0.1384806	0	0	0.00E+00	1	None
0.1385358	0	0	0.00E+00	1	None
0.1389959	0	0	0.00E+00	1	None
0.1391629	0	0	0.00E+00	1	None
0.1397022	0	0	0.00E+00	1	None
0.1402737	0	0	0.00E+00	1	None
0.1403094	0	0	0.00E+00	1	None
0.140614	0	0	0.00E+00	1	None
0.1406168	0	0	0.00E+00	1	None
0.1409937	0	0	0.00E+00	1	None
0.1410181	0	0	0.00E+00	1	None
0.1412668	0	0	0.00E+00	1	None
0.1412717	0	0	0.00E+00	1	None
0.1413506	0	0	0.00E+00	1	None
0.1416197	0	0	0.00E+00	1	None
0.1420243	0	0	0.00E+00	1	None
0.1420726	0	0	0.00E+00	1	None
0.1432529	0	0	0.00E+00	1	None
0.1433123	0	0	0.00E+00	1	None
0.1443586	0	0	0.00E+00	1	None
0.1443935	0	0	0.00E+00	1	None
0.1448429	0	0	0.00E+00	1	None
0.1448945	0	0	0.00E+00	1	None
0.145008	0	0	0.00E+00	1	None
0.1451322	0	0	0.00E+00	1	None

0.1456232	0	0	0.00E+00	1	None
0.145922	0	0	0.00E+00	1	None
0.1465388	0	0	0.00E+00	1	None
0.1472713	0	0	0.00E+00	1	None
0.1475453	0	0	0.00E+00	1	None
0.1479219	0	0	0.00E+00	1	None
0.1493054	0	0	0.00E+00	1	None
0.1497837	0	0	0.00E+00	1	None
0.1509127	0	0	0.00E+00	1	None
0.151405	0	0	0.00E+00	1	None
0.1517057	0	0	0.00E+00	1	None
0.1521114	0	0	0.00E+00	1	None
0.1521493	0	0	0.00E+00	1	None
0.1526215	0	0	0.00E+00	1	None
0.1528792	0	0	0.00E+00	1	None
0.1533257	0	0	0.00E+00	1	None
0.1533727	0	0	0.00E+00	1	None
0.1534597	0	0	0.00E+00	1	None
0.1536249	0	0	0.00E+00	1	None
0.1548138	0	0	0.00E+00	1	None
0.1572974	0	0	0.00E+00	1	None
0.1578208	0	0	0.00E+00	1	None
0.157842	0	0	0.00E+00	1	None
0.1584097	0	0	0.00E+00	1	None
0.1600018	0	0	0.00E+00	1	None
0.1615942	0	0	0.00E+00	1	None
0.1639793	0	0	0.00E+00	1	None
0.1640688	0	0	0.00E+00	1	None
0.1641911	0	0	0.00E+00	1	None
0.1679791	0	0	0.00E+00	1	None
0.1689961	0	0	0.00E+00	1	None
0.1706492	0	0	0.00E+00	1	None
0.1741342	0	0	0.00E+00	1	None
0.1750867	0	0	0.00E+00	1	None
0.175153	0	0	0.00E+00	1	None
0.1751531	0	0	0.00E+00	1	None
0.1755223	0	0	0.00E+00	1	None
0.1768201	0	0	0.00E+00	1	None
0.1782359	0	0	0.00E+00	1	None
0.1784742	0	0	0.00E+00	1	None
0.1786401	0	0	0.00E+00	1	None
0.1792124	0	0	0.00E+00	1	None
0.1802676	0	0	0.00E+00	1	None
0.1825603	0	0	0.00E+00	1	None
0.1827529	0	0	0.00E+00	1	None
0.1832845	0	0	0.00E+00	1	None
0.183433	0	0	0.00E+00	1	None
0.1835074	0	0	0.00E+00	1	None

0.1845822	0	0	0.00E+00	1	None
0.1847056	0	0	0.00E+00	1	None
0.1860792	0	0	0.00E+00	1	None
0.1876487	0	0	0.00E+00	1	None
0.1876779	0	0	0.00E+00	1	None
0.1881333	0	0	0.00E+00	1	None
0.1896526	0	0	0.00E+00	1	None
0.1898527	0	0	0.00E+00	1	None
0.1901865	0	0	0.00E+00	1	None
0.192052	0	0	0.00E+00	1	None
0.1920793	0	0	0.00E+00	1	None
0.1926022	0	0	0.00E+00	1	None
0.1930635	0	0	0.00E+00	1	None
0.1943289	0	0	0.00E+00	1	None
0.1946963	0	0	0.00E+00	1	None
0.1991878	0	0	0.00E+00	1	None
0.2027606	0	0	0.00E+00	1	None
0.204756	0	0	0.00E+00	1	None
0.20936	0	0	0.00E+00	1	None
0.2098129	0	0	0.00E+00	1	None
0.2108325	0	0	0.00E+00	1	None
0.2133745	0	0	0.00E+00	1	None
0.2138887	0	0	0.00E+00	1	None
0.2171761	0	0	0.00E+00	1	None
0.2193671	0	0	0.00E+00	1	None
0.2195255	0	0	0.00E+00	1	None
0.220542	0	0	0.00E+00	1	None
0.2212035	0	0	0.00E+00	1	None
0.2215812	0	0	0.00E+00	1	None
0.2277044	0	0	0.00E+00	1	None
0.2287748	0	0	0.00E+00	1	None
0.2290114	0	0	0.00E+00	1	None
0.2296988	0	0	0.00E+00	1	None
0.233278	0	0	0.00E+00	1	None
0.2359024	0	0	0.00E+00	1	None
0.2428996	0	0	0.00E+00	1	None
0.24356	0	0	0.00E+00	1	None
0.251763	0	0	0.00E+00	1	None
0.2521233	0	0	0.00E+00	1	None
0.2542157	0	0	0.00E+00	1	None
0.2580901	0	0	0.00E+00	1	None
0.261423	0	0	0.00E+00	1	None
0.2668267	0	0	0.00E+00	1	None
0.2812142	0	0	0.00E+00	1	None
0.2902194	0	0	0.00E+00	1	None
0.2941524	0	0	0.00E+00	1	None
0.3029261	0	0	0.00E+00	1	None
0.3060872	0	0	0.00E+00	1	None

0.3080405	0	0	0.00E+00	1	None
0.3092529	0	0	0.00E+00	1	None
0.3208	0	0	0.00E+00	1	None
0.3506363	0	0	0.00E+00	1	None
0.3549267	0	0	0.00E+00	1	None
0.3853662	0	0	0.00E+00	1	None
0.403764	0	0	0.00E+00	1	None
0.4066268	0	0	0.00E+00	1	None
0.4151107	0	0	0.00E+00	1	None
0.4629593	0	0	0.00E+00	1	None
0.4692358	0	0	0.00E+00	1	None
0.4876794	0	0	0.00E+00	1	None
0.5165521	0	0	0.00E+00	1	None
0.5241641	0	0	0.00E+00	1	None
0.5295047	0	0	0.00E+00	1	None
0.5513041	0	0	0.00E+00	1	None
0.551684	0	0	0.00E+00	1	None
0.5619494	0	0	0.00E+00	1	None
0.5918713	0	0	0.00E+00	1	None
0.7094639	0	0	0.00E+00	1	None
0.9289219	0	0	0.00E+00	1	None
0.9757418	0	0	0.00E+00	1	None

Net Benifit(predict time=3 year)

Thresholds	TPR	FPR	Net Benift	time	model
0.2324474	0.331350615	0.66864939	1.29E-01	3	RiskScore
0.2325616	0.331827441	0.66384355	1.31E-01	3	RiskScore
0.2326344	0.332313749	0.65902824	1.33E-01	3	RiskScore
0.2330315	0.331889838	0.65512315	1.33E-01	3	RiskScore
0.2334785	0.332381512	0.65030247	1.34E-01	3	RiskScore
0.2354691	0.332086623	0.64626836	1.33E-01	3	RiskScore
0.2358216	0.33221423	0.64181174	1.34E-01	3	RiskScore
0.2365157	0.332034748	0.63766222	1.34E-01	3	RiskScore
0.2367329	0.331294006	0.63407396	1.35E-01	3	RiskScore
0.2379089	0.330118859	0.6309201	1.33E-01	3	RiskScore
0.2380282	0.329684261	0.6270257	1.34E-01	3	RiskScore
0.2380918	0.330163809	0.62221714	1.36E-01	3	RiskScore
0.2382741	0.330653257	0.61739869	1.38E-01	3	RiskScore
0.2387183	0.330210456	0.61351249	1.38E-01	3	RiskScore
0.2390115	0.330705327	0.60868861	1.40E-01	3	RiskScore
0.2392151	0.330258882	0.60480605	1.40E-01	3	RiskScore
0.239685	0.330759317	0.59997661	1.42E-01	3	RiskScore
0.2399055	0.330695573	0.59571135	1.43E-01	3	RiskScore
0.2415421	0.331206143	0.59087178	1.43E-01	3	RiskScore
0.2415801	0.331727789	0.58602113	1.45E-01	3	RiskScore
0.2423293	0.332260914	0.581159	1.46E-01	3	RiskScore
0.2427557	0.332805938	0.57628497	1.48E-01	3	RiskScore

0.2435489	0.333363308	0.5713986	1.49E-01	3	RiskScore
0.2437227	0.333300254	0.56713265	1.51E-01	3	RiskScore
0.2438129	0.33296849	0.56313541	1.51E-01	3	RiskScore
0.2439761	0.33353374	0.55824115	1.53E-01	3	RiskScore
0.2440702	0.333570329	0.55387556	1.55E-01	3	RiskScore
0.2441745	0.333041128	0.55007575	1.55E-01	3	RiskScore
0.2442005	0.333080482	0.5457074	1.57E-01	3	RiskScore
0.2443748	0.324625754	0.54983312	1.47E-01	3	RiskScore
0.2444766	0.325143517	0.54498635	1.49E-01	3	RiskScore
0.2445014	0.324261154	0.54153971	1.49E-01	3	RiskScore
0.2449777	0.324782244	0.53668962	1.51E-01	3	RiskScore
0.2450869	0.320380964	0.53676189	1.46E-01	3	RiskScore
0.2452584	0.320499365	0.53231449	1.48E-01	3	RiskScore
0.2455181	0.320516165	0.52796868	1.49E-01	3	RiskScore
0.2455941	0.321052702	0.52310314	1.51E-01	3	RiskScore
0.2456544	0.321601915	0.51822492	1.53E-01	3	RiskScore
0.2456976	0.317244792	0.51825304	1.48E-01	3	RiskScore
0.2460349	0.317807158	0.51336167	1.50E-01	3	RiskScore
0.2464913	0.316543982	0.51029584	1.50E-01	3	RiskScore
0.2466409	0.316179311	0.50633151	1.50E-01	3	RiskScore
0.2466789	0.31484923	0.50333259	1.50E-01	3	RiskScore
0.2467769	0.310151169	0.50370164	1.45E-01	3	RiskScore
0.2473703	0.309629614	0.4998942	1.45E-01	3	RiskScore
0.2479825	0.304863723	0.50033108	1.40E-01	3	RiskScore
0.248144	0.305403201	0.4954626	1.42E-01	3	RiskScore
0.2483046	0.304829304	0.49170749	1.42E-01	3	RiskScore
0.2489092	0.304452006	0.48775579	1.43E-01	3	RiskScore
0.2492824	0.304992524	0.48288626	1.45E-01	3	RiskScore
0.2493277	0.305546744	0.47800304	1.47E-01	3	RiskScore
0.2493878	0.306115254	0.47310553	1.49E-01	3	RiskScore
0.2502877	0.305476105	0.46941567	1.49E-01	3	RiskScore
0.2503763	0.305382778	0.46517999	1.50E-01	3	RiskScore
0.2506317	0.305956734	0.46027703	1.52E-01	3	RiskScore
0.2508472	0.301428725	0.46047604	1.47E-01	3	RiskScore
0.2509394	0.295086378	0.46248938	1.40E-01	3	RiskScore
0.25095	0.295655501	0.45759125	1.42E-01	3	RiskScore
0.2519291	0.291097582	0.45782017	1.37E-01	3	RiskScore
0.25194	0.291681787	0.45290696	1.39E-01	3	RiskScore
0.2531149	0.291658847	0.44860089	1.40E-01	3	RiskScore
0.2532701	0.284236988	0.45169375	1.31E-01	3	RiskScore
0.2533178	0.284795582	0.44680615	1.33E-01	3	RiskScore
0.2542143	0.284434876	0.44283785	1.33E-01	3	RiskScore
0.2550458	0.284496293	0.43844743	1.34E-01	3	RiskScore
0.2552142	0.279899721	0.438715	1.30E-01	3	RiskScore
0.2552211	0.280469285	0.43381643	1.32E-01	3	RiskScore
0.2560055	0.279652034	0.43030468	1.32E-01	3	RiskScore
0.2567829	0.279610361	0.42601734	1.32E-01	3	RiskScore
0.2567938	0.279070914	0.42222779	1.33E-01	3	RiskScore

0.2569815	0.279637914	0.41733178	1.35E-01	3	RiskScore
0.2574549	0.280221579	0.41241911	1.37E-01	3	RiskScore
0.2577058	0.279988552	0.40832314	1.38E-01	3	RiskScore
0.2579967	0.280195208	0.40378748	1.40E-01	3	RiskScore
0.2593925	0.279813593	0.39984009	1.40E-01	3	RiskScore
0.2594091	0.280409364	0.39491531	1.42E-01	3	RiskScore
0.2598421	0.279497104	0.39149857	1.42E-01	3	RiskScore
0.2605503	0.280098617	0.38656805	1.44E-01	3	RiskScore
0.260862	0.280718842	0.38161882	1.46E-01	3	RiskScore
0.2610331	0.281358794	0.37664986	1.48E-01	3	RiskScore
0.2610496	0.279558774	0.37412088	1.47E-01	3	RiskScore
0.2616139	0.279618452	0.3697322	1.49E-01	3	RiskScore
0.2618651	0.279198081	0.36582356	1.49E-01	3	RiskScore
0.2618864	0.279835979	0.36085666	1.52E-01	3	RiskScore
0.2622943	0.280495154	0.35586848	1.54E-01	3	RiskScore
0.2623409	0.281176849	0.35085778	1.56E-01	3	RiskScore
0.2626937	0.280663063	0.34704256	1.57E-01	3	RiskScore
0.2627682	0.280492567	0.34288406	1.58E-01	3	RiskScore
0.2629297	0.281184398	0.33786322	1.61E-01	3	RiskScore
0.2629947	0.281900863	0.33281775	1.63E-01	3	RiskScore
0.2636046	0.280695646	0.32969396	1.63E-01	3	RiskScore
0.2638387	0.28142063	0.32463998	1.65E-01	3	RiskScore
0.2639136	0.281860531	0.31987107	1.67E-01	3	RiskScore
0.264326	0.276586185	0.32081641	1.61E-01	3	RiskScore
0.2647584	0.27563746	0.31743613	1.61E-01	3	RiskScore
0.2650185	0.275070305	0.31367428	1.62E-01	3	RiskScore
0.265403	0.275801748	0.30861384	1.64E-01	3	RiskScore
0.2654929	0.276561792	0.30352479	1.67E-01	3	RiskScore
0.2655384	0.275481661	0.30027591	1.67E-01	3	RiskScore
0.2671988	0.275049654	0.29637892	1.67E-01	3	RiskScore
0.2674995	0.275810814	0.29128875	1.69E-01	3	RiskScore
0.2690333	0.274804484	0.28796608	1.69E-01	3	RiskScore
0.2694073	0.275578075	0.28286348	1.71E-01	3	RiskScore
0.2705853	0.271213985	0.28289857	1.66E-01	3	RiskScore
0.270591	0.271665866	0.27811768	1.68E-01	3	RiskScore
0.2706129	0.269660815	0.27579373	1.67E-01	3	RiskScore
0.2706497	0.268944628	0.27218091	1.68E-01	3	RiskScore
0.2707486	0.269726996	0.26706954	1.71E-01	3	RiskScore
0.271573	0.265187515	0.26728002	1.66E-01	3	RiskScore
0.2718721	0.263431455	0.26470707	1.65E-01	3	RiskScore
0.2728376	0.258187341	0.26562218	1.59E-01	3	RiskScore
0.2738599	0.258973094	0.26050743	1.61E-01	3	RiskScore
0.2739238	0.258958337	0.25619318	1.62E-01	3	RiskScore
0.2744684	0.257616239	0.25320627	1.62E-01	3	RiskScore
0.2744734	0.258412836	0.24808067	1.65E-01	3	RiskScore
0.275147	0.259248072	0.24291643	1.67E-01	3	RiskScore
0.2751905	0.260125356	0.23771014	1.70E-01	3	RiskScore
0.2756347	0.255759106	0.23774739	1.65E-01	3	RiskScore

0.2756434	0.256682485	0.232495	1.68E-01	3 RiskScore
0.2757844	0.256332598	0.22851589	1.69E-01	3 RiskScore
0.2762649	0.256311916	0.22420756	1.71E-01	3 RiskScore
0.276987	0.255021292	0.22116918	1.70E-01	3 RiskScore
0.2770732	0.255949576	0.2159119	1.73E-01	3 RiskScore
0.2791775	0.256929882	0.21060259	1.75E-01	3 RiskScore
0.2792832	0.257453293	0.20575017	1.78E-01	3 RiskScore
0.2811456	0.257433297	0.20144116	1.79E-01	3 RiskScore
0.2812078	0.252262826	0.20228263	1.73E-01	3 RiskScore
0.2820068	0.253315645	0.19690081	1.76E-01	3 RiskScore
0.2820985	0.248753108	0.19713434	1.71E-01	3 RiskScore
0.2823003	0.244343505	0.19721494	1.67E-01	3 RiskScore
0.282521	0.2454629	0.19176654	1.70E-01	3 RiskScore
0.2833933	0.246654982	0.18624545	1.73E-01	3 RiskScore
0.2839238	0.245896606	0.18267482	1.73E-01	3 RiskScore
0.2850183	0.247139058	0.17710337	1.77E-01	3 RiskScore
0.2863171	0.248470756	0.17144266	1.80E-01	3 RiskScore
0.2868025	0.246233467	0.16935095	1.78E-01	3 RiskScore
0.2874694	0.2437073	0.16754811	1.76E-01	3 RiskScore
0.2899167	0.242273387	0.16465302	1.75E-01	3 RiskScore
0.2907617	0.243573113	0.15902429	1.78E-01	3 RiskScore
0.2927544	0.244981268	0.15328713	1.82E-01	3 RiskScore
0.2936223	0.244189711	0.14974968	1.82E-01	3 RiskScore
0.2941522	0.245680849	0.14392954	1.86E-01	3 RiskScore
0.2948668	0.239441978	0.14583941	1.78E-01	3 RiskScore
0.2949336	0.241047546	0.13990483	1.83E-01	3 RiskScore
0.2957647	0.242826148	0.13379723	1.87E-01	3 RiskScore
0.296218	0.238272452	0.13402192	1.82E-01	3 RiskScore
0.2970032	0.237999787	0.12996558	1.83E-01	3 RiskScore
0.2970859	0.239955385	0.12368098	1.88E-01	3 RiskScore
0.2972388	0.238232633	0.12107473	1.87E-01	3 RiskScore
0.2975292	0.237392852	0.1175855	1.88E-01	3 RiskScore
0.2996167	0.232269956	0.11837939	1.82E-01	3 RiskScore
0.3039672	0.22942116	0.11689919	1.78E-01	3 RiskScore
0.3048822	0.231424505	0.11056684	1.83E-01	3 RiskScore
0.3049193	0.224368969	0.11329337	1.75E-01	3 RiskScore
0.3059109	0.222747895	0.11058544	1.74E-01	3 RiskScore
0.3086881	0.218378402	0.11062593	1.69E-01	3 RiskScore
0.3114601	0.220525401	0.10414992	1.73E-01	3 RiskScore
0.3156007	0.215972029	0.10437429	1.68E-01	3 RiskScore
0.3157558	0.213507384	0.10250993	1.66E-01	3 RiskScore
0.3159679	0.215962102	0.09572621	1.72E-01	3 RiskScore
0.322515	0.207337519	0.10002179	1.60E-01	3 RiskScore
0.3242672	0.202803933	0.10022637	1.55E-01	3 RiskScore
0.3271102	0.202939354	0.09576194	1.56E-01	3 RiskScore
0.3330829	0.198147371	0.09622492	1.50E-01	3 RiskScore
0.3347105	0.193796926	0.09624636	1.45E-01	3 RiskScore
0.3348237	0.191273343	0.09444094	1.44E-01	3 RiskScore

0.3348239	0.190605088	0.09078019	1.45E-01	3	RiskScore
0.3354541	0.188864625	0.08819165	1.44E-01	3	RiskScore
0.3400765	0.191284271	0.081443	1.49E-01	3	RiskScore
0.3404817	0.18886886	0.07952941	1.48E-01	3	RiskScore
0.3407637	0.187549294	0.07651997	1.48E-01	3	RiskScore
0.3417358	0.187486748	0.07225351	1.50E-01	3	RiskScore
0.3435263	0.183076565	0.07233469	1.45E-01	3	RiskScore
0.3474078	0.182604706	0.06847754	1.46E-01	3	RiskScore
0.3477334	0.181237939	0.06551531	1.46E-01	3	RiskScore
0.3486314	0.176696377	0.06572787	1.42E-01	3	RiskScore
0.348882	0.179134765	0.05896047	1.48E-01	3	RiskScore
0.3510288	0.179013415	0.05475282	1.49E-01	3	RiskScore
0.356028	0.178974503	0.05046273	1.51E-01	3	RiskScore
0.3593381	0.167516681	0.05759154	1.35E-01	3	RiskScore
0.3642655	0.161405237	0.05937398	1.27E-01	3	RiskScore
0.3650343	0.157052473	0.05939774	1.23E-01	3	RiskScore
0.3677515	0.150599764	0.06152145	1.15E-01	3	RiskScore
0.3751946	0.146214945	0.06157726	1.09E-01	3	RiskScore
0.381082	0.141827615	0.06163559	1.04E-01	3	RiskScore
0.3843573	0.142937285	0.05619691	1.08E-01	3	RiskScore
0.3918795	0.138550091	0.0562551	1.02E-01	3	RiskScore
0.3926167	0.134160089	0.0563161	9.78E-02	3	RiskScore
0.3942749	0.129652479	0.05649471	9.29E-02	3	RiskScore
0.3983986	0.129887086	0.0519311	9.55E-02	3	RiskScore
0.3992309	0.123466473	0.0540227	8.76E-02	3	RiskScore
0.4045379	0.124601593	0.04855858	9.16E-02	3	RiskScore
0.4080611	0.123306788	0.04552438	9.19E-02	3	RiskScore
0.4083153	0.117796663	0.0467055	8.56E-02	3	RiskScore
0.4099458	0.118941521	0.04123164	9.03E-02	3	RiskScore
0.4116102	0.116074552	0.0397696	8.83E-02	3	RiskScore
0.4213668	0.111713056	0.0398021	8.27E-02	3	RiskScore
0.4230637	0.106045575	0.04114057	7.59E-02	3	RiskScore
0.4234383	0.104820423	0.03803672	7.69E-02	3	RiskScore
0.4245261	0.105800866	0.03272727	8.17E-02	3	RiskScore
0.430173	0.102963129	0.03123601	7.94E-02	3	RiskScore
0.4342949	0.101525459	0.02834467	7.98E-02	3	RiskScore
0.4452072	0.097126467	0.02841466	7.43E-02	3	RiskScore
0.4462314	0.092722093	0.02849003	6.98E-02	3	RiskScore
0.4588693	0.088311688	0.02857143	6.41E-02	3	RiskScore
0.4594209	0.08649992	0.02605419	6.44E-02	3	RiskScore
0.4626181	0.087050631	0.02117448	6.88E-02	3	RiskScore
0.4685119	0.087704503	0.0161916	7.34E-02	3	RiskScore
0.4735545	0.083311247	0.01625585	6.87E-02	3	RiskScore
0.4816763	0.079689018	0.01554908	6.52E-02	3	RiskScore
0.5029764	0.075324675	0.01558442	5.96E-02	3	RiskScore
0.5160684	0.070956612	0.01562347	5.43E-02	3	RiskScore
0.5217284	0.082251082	0	8.23E-02	3	RiskScore
0.5342283	0.077922078	0	7.79E-02	3	RiskScore

0.5386892	0.073593074	0	7.36E-02	3 RiskScore
0.5431337	0.069264069	0	6.93E-02	3 RiskScore
0.6047391	0.064935065	0	6.49E-02	3 RiskScore
0.6431908	0.060606061	0	6.06E-02	3 RiskScore
0.6654267	0.056277056	0	5.63E-02	3 RiskScore
0.6688188	0.051948052	0	5.19E-02	3 RiskScore
0.6787647	0.047619048	0	4.76E-02	3 RiskScore
0.7318719	0.043290043	0	4.33E-02	3 RiskScore
0.7384636	0.038961039	0	3.90E-02	3 RiskScore
0.7924715	0.034632035	0	3.46E-02	3 RiskScore
0.7973723	0.03030303	0	3.03E-02	3 RiskScore
0.817065	0.025974026	0	2.60E-02	3 RiskScore
0.8258206	0.021645022	0	2.16E-02	3 RiskScore
0.8500529	0.017316017	0	1.73E-02	3 RiskScore
0.9269821	0.012987013	0	1.30E-02	3 RiskScore
0.9962951	0.008658009	0	8.66E-03	3 RiskScore
0.9996196	0.004329004	0	4.33E-03	3 RiskScore
0.3294786	0.331350615	0.66864939	2.79E-03	3 Age
0.3324022	0.084620243	0.21841006	-2.41E-02	3 Age
0.3072028	0.331350615	0.66864939	3.49E-02	3 Gender
0.3759724	0.116323075	0.19536524	-1.38E-03	3 Gender
0.3256031	0.331350615	0.66864939	8.52E-03	3 Grade
0.3290671	0.281406449	0.59305243	-9.46E-03	3 Grade
0.3325583	0.148235976	0.28899346	4.24E-03	3 Grade
0.3360766	0.017316017	0.02597403	4.17E-03	3 Grade
0.1951761	0.331350615	0.66864939	1.69E-01	3 Stage
0.3349926	0.243641307	0.2671812	1.09E-01	3 Stage
0.5353581	0.175578684	0.12312261	3.37E-02	3 Stage
0.763099	0.017316017	0	1.73E-02	3 Stage
0.2008128	0.331350615	0.66864939	1.63E-01	3 T.Stage
0.3344472	0.232529109	0.26963539	9.70E-02	3 T.Stage
0.5226355	0.166407596	0.11930669	3.58E-02	3 T.Stage
0.738963	0.043290043	0	4.33E-02	3 T.Stage
0.3257815	0.331350615	0.66864939	8.26E-03	3 N.Stage
0.5580813	0.017316017	0	1.73E-02	3 N.Stage
0.3212641	0.331350615	0.66864939	1.49E-02	3 M.Stage
0.7817184	0.012987013	0	1.30E-02	3 M.Stage
0	0.331350615	0.66864939	3.31E-01	3 All
0.1951761	0.331350615	0.66864939	1.69E-01	3 All
0.2008128	0.331350615	0.66864939	1.63E-01	3 All
0.2324474	0.331350615	0.66864939	1.29E-01	3 All
0.2325616	0.331350615	0.66864939	1.29E-01	3 All
0.2326344	0.331350615	0.66864939	1.29E-01	3 All
0.2330315	0.331350615	0.66864939	1.28E-01	3 All
0.2334785	0.331350615	0.66864939	1.28E-01	3 All
0.2354691	0.331350615	0.66864939	1.25E-01	3 All
0.2358216	0.331350615	0.66864939	1.25E-01	3 All
0.2365157	0.331350615	0.66864939	1.24E-01	3 All

0.2367329	0.331350615	0.66864939	1.24E-01	3 All
0.2379089	0.331350615	0.66864939	1.23E-01	3 All
0.2380282	0.331350615	0.66864939	1.22E-01	3 All
0.2380918	0.331350615	0.66864939	1.22E-01	3 All
0.2382741	0.331350615	0.66864939	1.22E-01	3 All
0.2387183	0.331350615	0.66864939	1.22E-01	3 All
0.2390115	0.331350615	0.66864939	1.21E-01	3 All
0.2392151	0.331350615	0.66864939	1.21E-01	3 All
0.239685	0.331350615	0.66864939	1.21E-01	3 All
0.2399055	0.331350615	0.66864939	1.20E-01	3 All
0.2415421	0.331350615	0.66864939	1.18E-01	3 All
0.2415801	0.331350615	0.66864939	1.18E-01	3 All
0.2423293	0.331350615	0.66864939	1.17E-01	3 All
0.2427557	0.331350615	0.66864939	1.17E-01	3 All
0.2435489	0.331350615	0.66864939	1.16E-01	3 All
0.2437227	0.331350615	0.66864939	1.16E-01	3 All
0.2438129	0.331350615	0.66864939	1.16E-01	3 All
0.2439761	0.331350615	0.66864939	1.16E-01	3 All
0.2440702	0.331350615	0.66864939	1.15E-01	3 All
0.2441745	0.331350615	0.66864939	1.15E-01	3 All
0.2442005	0.331350615	0.66864939	1.15E-01	3 All
0.2443748	0.331350615	0.66864939	1.15E-01	3 All
0.2444766	0.331350615	0.66864939	1.15E-01	3 All
0.2445014	0.331350615	0.66864939	1.15E-01	3 All
0.2449777	0.331350615	0.66864939	1.14E-01	3 All
0.2450869	0.331350615	0.66864939	1.14E-01	3 All
0.2452584	0.331350615	0.66864939	1.14E-01	3 All
0.2455181	0.331350615	0.66864939	1.14E-01	3 All
0.2455941	0.331350615	0.66864939	1.14E-01	3 All
0.2456544	0.331350615	0.66864939	1.14E-01	3 All
0.2456976	0.331350615	0.66864939	1.14E-01	3 All
0.2460349	0.331350615	0.66864939	1.13E-01	3 All
0.2464913	0.331350615	0.66864939	1.13E-01	3 All
0.2466409	0.331350615	0.66864939	1.12E-01	3 All
0.2466789	0.331350615	0.66864939	1.12E-01	3 All
0.2467769	0.331350615	0.66864939	1.12E-01	3 All
0.2473703	0.331350615	0.66864939	1.12E-01	3 All
0.2479825	0.331350615	0.66864939	1.11E-01	3 All
0.248144	0.331350615	0.66864939	1.11E-01	3 All
0.2483046	0.331350615	0.66864939	1.10E-01	3 All
0.2489092	0.331350615	0.66864939	1.10E-01	3 All
0.2492824	0.331350615	0.66864939	1.09E-01	3 All
0.2493277	0.331350615	0.66864939	1.09E-01	3 All
0.2493878	0.331350615	0.66864939	1.09E-01	3 All
0.2502877	0.331350615	0.66864939	1.08E-01	3 All
0.2503763	0.331350615	0.66864939	1.08E-01	3 All
0.2506317	0.331350615	0.66864939	1.08E-01	3 All
0.2508472	0.331350615	0.66864939	1.07E-01	3 All

0.2509394	0.331350615	0.66864939	1.07E-01	3 All
0.25095	0.331350615	0.66864939	1.07E-01	3 All
0.2519291	0.331350615	0.66864939	1.06E-01	3 All
0.25194	0.331350615	0.66864939	1.06E-01	3 All
0.2531149	0.331350615	0.66864939	1.05E-01	3 All
0.2532701	0.331350615	0.66864939	1.05E-01	3 All
0.2533178	0.331350615	0.66864939	1.05E-01	3 All
0.2542143	0.331350615	0.66864939	1.03E-01	3 All
0.2550458	0.331350615	0.66864939	1.02E-01	3 All
0.2552142	0.331350615	0.66864939	1.02E-01	3 All
0.2552211	0.331350615	0.66864939	1.02E-01	3 All
0.2560055	0.331350615	0.66864939	1.01E-01	3 All
0.2567829	0.331350615	0.66864939	1.00E-01	3 All
0.2567938	0.331350615	0.66864939	1.00E-01	3 All
0.2569815	0.331350615	0.66864939	1.00E-01	3 All
0.2574549	0.331350615	0.66864939	9.95E-02	3 All
0.2577058	0.331350615	0.66864939	9.92E-02	3 All
0.2579967	0.331350615	0.66864939	9.89E-02	3 All
0.2593925	0.331350615	0.66864939	9.72E-02	3 All
0.2594091	0.331350615	0.66864939	9.71E-02	3 All
0.2598421	0.331350615	0.66864939	9.66E-02	3 All
0.2605503	0.331350615	0.66864939	9.57E-02	3 All
0.260862	0.331350615	0.66864939	9.54E-02	3 All
0.2610331	0.331350615	0.66864939	9.52E-02	3 All
0.2610496	0.331350615	0.66864939	9.51E-02	3 All
0.2616139	0.331350615	0.66864939	9.44E-02	3 All
0.2618651	0.331350615	0.66864939	9.41E-02	3 All
0.2618864	0.331350615	0.66864939	9.41E-02	3 All
0.2622943	0.331350615	0.66864939	9.36E-02	3 All
0.2623409	0.331350615	0.66864939	9.36E-02	3 All
0.2626937	0.331350615	0.66864939	9.31E-02	3 All
0.2627682	0.331350615	0.66864939	9.30E-02	3 All
0.2629297	0.331350615	0.66864939	9.28E-02	3 All
0.2629947	0.331350615	0.66864939	9.27E-02	3 All
0.2636046	0.331350615	0.66864939	9.20E-02	3 All
0.2638387	0.331350615	0.66864939	9.17E-02	3 All
0.2639136	0.331350615	0.66864939	9.16E-02	3 All
0.264326	0.331350615	0.66864939	9.11E-02	3 All
0.2647584	0.331350615	0.66864939	9.06E-02	3 All
0.2650185	0.331350615	0.66864939	9.03E-02	3 All
0.265403	0.331350615	0.66864939	8.98E-02	3 All
0.2654929	0.331350615	0.66864939	8.97E-02	3 All
0.2655384	0.331350615	0.66864939	8.96E-02	3 All
0.2671988	0.331350615	0.66864939	8.75E-02	3 All
0.2674995	0.331350615	0.66864939	8.72E-02	3 All
0.2690333	0.331350615	0.66864939	8.53E-02	3 All
0.2694073	0.331350615	0.66864939	8.48E-02	3 All
0.2705853	0.331350615	0.66864939	8.33E-02	3 All

0.270591	0.331350615	0.66864939	8.33E-02	3 All
0.2706129	0.331350615	0.66864939	8.33E-02	3 All
0.2706497	0.331350615	0.66864939	8.32E-02	3 All
0.2707486	0.331350615	0.66864939	8.31E-02	3 All
0.271573	0.331350615	0.66864939	8.21E-02	3 All
0.2718721	0.331350615	0.66864939	8.17E-02	3 All
0.2728376	0.331350615	0.66864939	8.05E-02	3 All
0.2738599	0.331350615	0.66864939	7.92E-02	3 All
0.2739238	0.331350615	0.66864939	7.91E-02	3 All
0.2744684	0.331350615	0.66864939	7.84E-02	3 All
0.2744734	0.331350615	0.66864939	7.84E-02	3 All
0.275147	0.331350615	0.66864939	7.75E-02	3 All
0.2751905	0.331350615	0.66864939	7.75E-02	3 All
0.2756347	0.331350615	0.66864939	7.69E-02	3 All
0.2756434	0.331350615	0.66864939	7.69E-02	3 All
0.2757844	0.331350615	0.66864939	7.67E-02	3 All
0.2762649	0.331350615	0.66864939	7.61E-02	3 All
0.276987	0.331350615	0.66864939	7.52E-02	3 All
0.2770732	0.331350615	0.66864939	7.51E-02	3 All
0.2791775	0.331350615	0.66864939	7.24E-02	3 All
0.2792832	0.331350615	0.66864939	7.22E-02	3 All
0.2811456	0.331350615	0.66864939	6.98E-02	3 All
0.2812078	0.331350615	0.66864939	6.98E-02	3 All
0.2820068	0.331350615	0.66864939	6.87E-02	3 All
0.2820985	0.331350615	0.66864939	6.86E-02	3 All
0.2823003	0.331350615	0.66864939	6.83E-02	3 All
0.282521	0.331350615	0.66864939	6.81E-02	3 All
0.2833933	0.331350615	0.66864939	6.69E-02	3 All
0.2839238	0.331350615	0.66864939	6.62E-02	3 All
0.2850183	0.331350615	0.66864939	6.48E-02	3 All
0.2863171	0.331350615	0.66864939	6.31E-02	3 All
0.2868025	0.331350615	0.66864939	6.25E-02	3 All
0.2874694	0.331350615	0.66864939	6.16E-02	3 All
0.2899167	0.331350615	0.66864939	5.84E-02	3 All
0.2907617	0.331350615	0.66864939	5.72E-02	3 All
0.2927544	0.331350615	0.66864939	5.46E-02	3 All
0.2936223	0.331350615	0.66864939	5.34E-02	3 All
0.2941522	0.331350615	0.66864939	5.27E-02	3 All
0.2948668	0.331350615	0.66864939	5.17E-02	3 All
0.2949336	0.331350615	0.66864939	5.17E-02	3 All
0.2957647	0.331350615	0.66864939	5.05E-02	3 All
0.296218	0.331350615	0.66864939	4.99E-02	3 All
0.2970032	0.331350615	0.66864939	4.89E-02	3 All
0.2970859	0.331350615	0.66864939	4.87E-02	3 All
0.2972388	0.331350615	0.66864939	4.85E-02	3 All
0.2975292	0.331350615	0.66864939	4.81E-02	3 All
0.2996167	0.331350615	0.66864939	4.53E-02	3 All
0.3039672	0.331350615	0.66864939	3.93E-02	3 All

0.3048822	0.331350615	0.66864939	3.81E-02	3	All
0.3049193	0.331350615	0.66864939	3.80E-02	3	All
0.3059109	0.331350615	0.66864939	3.67E-02	3	All
0.3072028	0.331350615	0.66864939	3.49E-02	3	All
0.3086881	0.331350615	0.66864939	3.28E-02	3	All
0.3114601	0.331350615	0.66864939	2.89E-02	3	All
0.3156007	0.331350615	0.66864939	2.30E-02	3	All
0.3157558	0.331350615	0.66864939	2.28E-02	3	All
0.3159679	0.331350615	0.66864939	2.25E-02	3	All
0.3212641	0.331350615	0.66864939	1.49E-02	3	All
0.322515	0.331350615	0.66864939	1.30E-02	3	All
0.3242672	0.331350615	0.66864939	1.05E-02	3	All
0.3256031	0.331350615	0.66864939	8.52E-03	3	All
0.3257815	0.331350615	0.66864939	8.26E-03	3	All
0.3271102	0.331350615	0.66864939	6.30E-03	3	All
0.3290671	0.331350615	0.66864939	3.40E-03	3	All
0.3294786	0.331350615	0.66864939	2.79E-03	3	All
0.3324022	0.331350615	0.66864939	-1.58E-03	3	All
0.3325583	0.331350615	0.66864939	-1.81E-03	3	All
0.3330829	0.331350615	0.66864939	-2.60E-03	3	All
0.3344472	0.331350615	0.66864939	-4.65E-03	3	All
0.3347105	0.331350615	0.66864939	-5.05E-03	3	All
0.3348237	0.331350615	0.66864939	-5.22E-03	3	All
0.3348239	0.331350615	0.66864939	-5.22E-03	3	All
0.3349926	0.331350615	0.66864939	-5.48E-03	3	All
0.3354541	0.331350615	0.66864939	-6.17E-03	3	All
0.3360766	0.331350615	0.66864939	-7.12E-03	3	All
0.3400765	0.331350615	0.66864939	-1.32E-02	3	All
0.3404817	0.331350615	0.66864939	-1.38E-02	3	All
0.3407637	0.331350615	0.66864939	-1.43E-02	3	All
0.3417358	0.331350615	0.66864939	-1.58E-02	3	All
0.3435263	0.331350615	0.66864939	-1.85E-02	3	All
0.3474078	0.331350615	0.66864939	-2.46E-02	3	All
0.3477334	0.331350615	0.66864939	-2.51E-02	3	All
0.3486314	0.331350615	0.66864939	-2.65E-02	3	All
0.348882	0.331350615	0.66864939	-2.69E-02	3	All
0.3510288	0.331350615	0.66864939	-3.03E-02	3	All
0.356028	0.331350615	0.66864939	-3.83E-02	3	All
0.3593381	0.331350615	0.66864939	-4.37E-02	3	All
0.3642655	0.331350615	0.66864939	-5.18E-02	3	All
0.3650343	0.331350615	0.66864939	-5.30E-02	3	All
0.3677515	0.331350615	0.66864939	-5.76E-02	3	All
0.3751946	0.331350615	0.66864939	-7.02E-02	3	All
0.3759724	0.331350615	0.66864939	-7.15E-02	3	All
0.381082	0.331350615	0.66864939	-8.04E-02	3	All
0.3843573	0.331350615	0.66864939	-8.61E-02	3	All
0.3918795	0.331350615	0.66864939	-9.95E-02	3	All
0.3926167	0.331350615	0.66864939	-1.01E-01	3	All

0.3942749	0.331350615	0.66864939	-1.04E-01	3	All
0.3983986	0.331350615	0.66864939	-1.11E-01	3	All
0.3992309	0.331350615	0.66864939	-1.13E-01	3	All
0.4045379	0.331350615	0.66864939	-1.23E-01	3	All
0.4080611	0.331350615	0.66864939	-1.30E-01	3	All
0.4083153	0.331350615	0.66864939	-1.30E-01	3	All
0.4099458	0.331350615	0.66864939	-1.33E-01	3	All
0.4116102	0.331350615	0.66864939	-1.36E-01	3	All
0.4213668	0.331350615	0.66864939	-1.56E-01	3	All
0.4230637	0.331350615	0.66864939	-1.59E-01	3	All
0.4234383	0.331350615	0.66864939	-1.60E-01	3	All
0.4245261	0.331350615	0.66864939	-1.62E-01	3	All
0.430173	0.331350615	0.66864939	-1.73E-01	3	All
0.4342949	0.331350615	0.66864939	-1.82E-01	3	All
0.4452072	0.331350615	0.66864939	-2.05E-01	3	All
0.4462314	0.331350615	0.66864939	-2.07E-01	3	All
0.4588693	0.331350615	0.66864939	-2.36E-01	3	All
0.4594209	0.331350615	0.66864939	-2.37E-01	3	All
0.4626181	0.331350615	0.66864939	-2.44E-01	3	All
0.4685119	0.331350615	0.66864939	-2.58E-01	3	All
0.4735545	0.331350615	0.66864939	-2.70E-01	3	All
0.4816763	0.331350615	0.66864939	-2.90E-01	3	All
0.5029764	0.331350615	0.66864939	-3.45E-01	3	All
0.5160684	0.331350615	0.66864939	-3.82E-01	3	All
0.5217284	0.331350615	0.66864939	-3.98E-01	3	All
0.5226355	0.331350615	0.66864939	-4.01E-01	3	All
0.5342283	0.331350615	0.66864939	-4.36E-01	3	All
0.5353581	0.331350615	0.66864939	-4.39E-01	3	All
0.5386892	0.331350615	0.66864939	-4.49E-01	3	All
0.5431337	0.331350615	0.66864939	-4.64E-01	3	All
0.5580813	0.331350615	0.66864939	-5.13E-01	3	All
0.6047391	0.331350615	0.66864939	-6.92E-01	3	All
0.6431908	0.331350615	0.66864939	-8.74E-01	3	All
0.6654267	0.331350615	0.66864939	-9.99E-01	3	All
0.6688188	0.331350615	0.66864939	-1.02E+00	3	All
0.6787647	0.331350615	0.66864939	-1.08E+00	3	All
0.7318719	0.331350615	0.66864939	-1.49E+00	3	All
0.7384636	0.331350615	0.66864939	-1.56E+00	3	All
0.738963	0.331350615	0.66864939	-1.56E+00	3	All
0.763099	0.331350615	0.66864939	-1.82E+00	3	All
0.7817184	0.331350615	0.66864939	-2.06E+00	3	All
0.7924715	0.331350615	0.66864939	-2.22E+00	3	All
0.7973723	0.331350615	0.66864939	-2.30E+00	3	All
0.817065	0.331350615	0.66864939	-2.66E+00	3	All
0.8258206	0.331350615	0.66864939	-2.84E+00	3	All
0.8500529	0.331350615	0.66864939	-3.46E+00	3	All
0.9269821	0.331350615	0.66864939	-8.16E+00	3	All
0.9962951	0.331350615	0.66864939	-1.79E+02	3	All

0.9996196	0.331350615	0.66864939	-1.76E+03	3	All
0	0	0	0.00E+00	3	None
0.1951761	0	0	0.00E+00	3	None
0.2008128	0	0	0.00E+00	3	None
0.2324474	0	0	0.00E+00	3	None
0.2325616	0	0	0.00E+00	3	None
0.2326344	0	0	0.00E+00	3	None
0.2330315	0	0	0.00E+00	3	None
0.2334785	0	0	0.00E+00	3	None
0.2354691	0	0	0.00E+00	3	None
0.2358216	0	0	0.00E+00	3	None
0.2365157	0	0	0.00E+00	3	None
0.2367329	0	0	0.00E+00	3	None
0.2379089	0	0	0.00E+00	3	None
0.2380282	0	0	0.00E+00	3	None
0.2380918	0	0	0.00E+00	3	None
0.2382741	0	0	0.00E+00	3	None
0.2387183	0	0	0.00E+00	3	None
0.2390115	0	0	0.00E+00	3	None
0.2392151	0	0	0.00E+00	3	None
0.239685	0	0	0.00E+00	3	None
0.2399055	0	0	0.00E+00	3	None
0.2415421	0	0	0.00E+00	3	None
0.2415801	0	0	0.00E+00	3	None
0.2423293	0	0	0.00E+00	3	None
0.2427557	0	0	0.00E+00	3	None
0.2435489	0	0	0.00E+00	3	None
0.2437227	0	0	0.00E+00	3	None
0.2438129	0	0	0.00E+00	3	None
0.2439761	0	0	0.00E+00	3	None
0.2440702	0	0	0.00E+00	3	None
0.2441745	0	0	0.00E+00	3	None
0.2442005	0	0	0.00E+00	3	None
0.2443748	0	0	0.00E+00	3	None
0.2444766	0	0	0.00E+00	3	None
0.2445014	0	0	0.00E+00	3	None
0.2449777	0	0	0.00E+00	3	None
0.2450869	0	0	0.00E+00	3	None
0.2452584	0	0	0.00E+00	3	None
0.2455181	0	0	0.00E+00	3	None
0.2455941	0	0	0.00E+00	3	None
0.2456544	0	0	0.00E+00	3	None
0.2456976	0	0	0.00E+00	3	None
0.2460349	0	0	0.00E+00	3	None
0.2464913	0	0	0.00E+00	3	None
0.2466409	0	0	0.00E+00	3	None
0.2466789	0	0	0.00E+00	3	None
0.2467769	0	0	0.00E+00	3	None

0.2473703	0	0	0.00E+00	3	None
0.2479825	0	0	0.00E+00	3	None
0.248144	0	0	0.00E+00	3	None
0.2483046	0	0	0.00E+00	3	None
0.2489092	0	0	0.00E+00	3	None
0.2492824	0	0	0.00E+00	3	None
0.2493277	0	0	0.00E+00	3	None
0.2493878	0	0	0.00E+00	3	None
0.2502877	0	0	0.00E+00	3	None
0.2503763	0	0	0.00E+00	3	None
0.2506317	0	0	0.00E+00	3	None
0.2508472	0	0	0.00E+00	3	None
0.2509394	0	0	0.00E+00	3	None
0.25095	0	0	0.00E+00	3	None
0.2519291	0	0	0.00E+00	3	None
0.25194	0	0	0.00E+00	3	None
0.2531149	0	0	0.00E+00	3	None
0.2532701	0	0	0.00E+00	3	None
0.2533178	0	0	0.00E+00	3	None
0.2542143	0	0	0.00E+00	3	None
0.2550458	0	0	0.00E+00	3	None
0.2552142	0	0	0.00E+00	3	None
0.2552211	0	0	0.00E+00	3	None
0.2560055	0	0	0.00E+00	3	None
0.2567829	0	0	0.00E+00	3	None
0.2567938	0	0	0.00E+00	3	None
0.2569815	0	0	0.00E+00	3	None
0.2574549	0	0	0.00E+00	3	None
0.2577058	0	0	0.00E+00	3	None
0.2579967	0	0	0.00E+00	3	None
0.2593925	0	0	0.00E+00	3	None
0.2594091	0	0	0.00E+00	3	None
0.2598421	0	0	0.00E+00	3	None
0.2605503	0	0	0.00E+00	3	None
0.260862	0	0	0.00E+00	3	None
0.2610331	0	0	0.00E+00	3	None
0.2610496	0	0	0.00E+00	3	None
0.2616139	0	0	0.00E+00	3	None
0.2618651	0	0	0.00E+00	3	None
0.2618864	0	0	0.00E+00	3	None
0.2622943	0	0	0.00E+00	3	None
0.2623409	0	0	0.00E+00	3	None
0.2626937	0	0	0.00E+00	3	None
0.2627682	0	0	0.00E+00	3	None
0.2629297	0	0	0.00E+00	3	None
0.2629947	0	0	0.00E+00	3	None
0.2636046	0	0	0.00E+00	3	None
0.2638387	0	0	0.00E+00	3	None

0.2639136	0	0	0.00E+00	3	None
0.264326	0	0	0.00E+00	3	None
0.2647584	0	0	0.00E+00	3	None
0.2650185	0	0	0.00E+00	3	None
0.265403	0	0	0.00E+00	3	None
0.2654929	0	0	0.00E+00	3	None
0.2655384	0	0	0.00E+00	3	None
0.2671988	0	0	0.00E+00	3	None
0.2674995	0	0	0.00E+00	3	None
0.2690333	0	0	0.00E+00	3	None
0.2694073	0	0	0.00E+00	3	None
0.2705853	0	0	0.00E+00	3	None
0.270591	0	0	0.00E+00	3	None
0.2706129	0	0	0.00E+00	3	None
0.2706497	0	0	0.00E+00	3	None
0.2707486	0	0	0.00E+00	3	None
0.271573	0	0	0.00E+00	3	None
0.2718721	0	0	0.00E+00	3	None
0.2728376	0	0	0.00E+00	3	None
0.2738599	0	0	0.00E+00	3	None
0.2739238	0	0	0.00E+00	3	None
0.2744684	0	0	0.00E+00	3	None
0.2744734	0	0	0.00E+00	3	None
0.275147	0	0	0.00E+00	3	None
0.2751905	0	0	0.00E+00	3	None
0.2756347	0	0	0.00E+00	3	None
0.2756434	0	0	0.00E+00	3	None
0.2757844	0	0	0.00E+00	3	None
0.2762649	0	0	0.00E+00	3	None
0.276987	0	0	0.00E+00	3	None
0.2770732	0	0	0.00E+00	3	None
0.2791775	0	0	0.00E+00	3	None
0.2792832	0	0	0.00E+00	3	None
0.2811456	0	0	0.00E+00	3	None
0.2812078	0	0	0.00E+00	3	None
0.2820068	0	0	0.00E+00	3	None
0.2820985	0	0	0.00E+00	3	None
0.2823003	0	0	0.00E+00	3	None
0.282521	0	0	0.00E+00	3	None
0.2833933	0	0	0.00E+00	3	None
0.2839238	0	0	0.00E+00	3	None
0.2850183	0	0	0.00E+00	3	None
0.2863171	0	0	0.00E+00	3	None
0.2868025	0	0	0.00E+00	3	None
0.2874694	0	0	0.00E+00	3	None
0.2899167	0	0	0.00E+00	3	None
0.2907617	0	0	0.00E+00	3	None
0.2927544	0	0	0.00E+00	3	None

0.2936223	0	0	0.00E+00	3	None
0.2941522	0	0	0.00E+00	3	None
0.2948668	0	0	0.00E+00	3	None
0.2949336	0	0	0.00E+00	3	None
0.2957647	0	0	0.00E+00	3	None
0.296218	0	0	0.00E+00	3	None
0.2970032	0	0	0.00E+00	3	None
0.2970859	0	0	0.00E+00	3	None
0.2972388	0	0	0.00E+00	3	None
0.2975292	0	0	0.00E+00	3	None
0.2996167	0	0	0.00E+00	3	None
0.3039672	0	0	0.00E+00	3	None
0.3048822	0	0	0.00E+00	3	None
0.3049193	0	0	0.00E+00	3	None
0.3059109	0	0	0.00E+00	3	None
0.3072028	0	0	0.00E+00	3	None
0.3086881	0	0	0.00E+00	3	None
0.3114601	0	0	0.00E+00	3	None
0.3156007	0	0	0.00E+00	3	None
0.3157558	0	0	0.00E+00	3	None
0.3159679	0	0	0.00E+00	3	None
0.3212641	0	0	0.00E+00	3	None
0.322515	0	0	0.00E+00	3	None
0.3242672	0	0	0.00E+00	3	None
0.3256031	0	0	0.00E+00	3	None
0.3257815	0	0	0.00E+00	3	None
0.3271102	0	0	0.00E+00	3	None
0.3290671	0	0	0.00E+00	3	None
0.3294786	0	0	0.00E+00	3	None
0.3324022	0	0	0.00E+00	3	None
0.3325583	0	0	0.00E+00	3	None
0.3330829	0	0	0.00E+00	3	None
0.3344472	0	0	0.00E+00	3	None
0.3347105	0	0	0.00E+00	3	None
0.3348237	0	0	0.00E+00	3	None
0.3348239	0	0	0.00E+00	3	None
0.3349926	0	0	0.00E+00	3	None
0.3354541	0	0	0.00E+00	3	None
0.3360766	0	0	0.00E+00	3	None
0.3400765	0	0	0.00E+00	3	None
0.3404817	0	0	0.00E+00	3	None
0.3407637	0	0	0.00E+00	3	None
0.3417358	0	0	0.00E+00	3	None
0.3435263	0	0	0.00E+00	3	None
0.3474078	0	0	0.00E+00	3	None
0.3477334	0	0	0.00E+00	3	None
0.3486314	0	0	0.00E+00	3	None
0.348882	0	0	0.00E+00	3	None

0.3510288	0	0	0.00E+00	3	None
0.356028	0	0	0.00E+00	3	None
0.3593381	0	0	0.00E+00	3	None
0.3642655	0	0	0.00E+00	3	None
0.3650343	0	0	0.00E+00	3	None
0.3677515	0	0	0.00E+00	3	None
0.3751946	0	0	0.00E+00	3	None
0.3759724	0	0	0.00E+00	3	None
0.381082	0	0	0.00E+00	3	None
0.3843573	0	0	0.00E+00	3	None
0.3918795	0	0	0.00E+00	3	None
0.3926167	0	0	0.00E+00	3	None
0.3942749	0	0	0.00E+00	3	None
0.3983986	0	0	0.00E+00	3	None
0.3992309	0	0	0.00E+00	3	None
0.4045379	0	0	0.00E+00	3	None
0.4080611	0	0	0.00E+00	3	None
0.4083153	0	0	0.00E+00	3	None
0.4099458	0	0	0.00E+00	3	None
0.4116102	0	0	0.00E+00	3	None
0.4213668	0	0	0.00E+00	3	None
0.4230637	0	0	0.00E+00	3	None
0.4234383	0	0	0.00E+00	3	None
0.4245261	0	0	0.00E+00	3	None
0.430173	0	0	0.00E+00	3	None
0.4342949	0	0	0.00E+00	3	None
0.4452072	0	0	0.00E+00	3	None
0.4462314	0	0	0.00E+00	3	None
0.4588693	0	0	0.00E+00	3	None
0.4594209	0	0	0.00E+00	3	None
0.4626181	0	0	0.00E+00	3	None
0.4685119	0	0	0.00E+00	3	None
0.4735545	0	0	0.00E+00	3	None
0.4816763	0	0	0.00E+00	3	None
0.5029764	0	0	0.00E+00	3	None
0.5160684	0	0	0.00E+00	3	None
0.5217284	0	0	0.00E+00	3	None
0.5226355	0	0	0.00E+00	3	None
0.5342283	0	0	0.00E+00	3	None
0.5353581	0	0	0.00E+00	3	None
0.5386892	0	0	0.00E+00	3	None
0.5431337	0	0	0.00E+00	3	None
0.5580813	0	0	0.00E+00	3	None
0.6047391	0	0	0.00E+00	3	None
0.6431908	0	0	0.00E+00	3	None
0.6654267	0	0	0.00E+00	3	None
0.6688188	0	0	0.00E+00	3	None
0.6787647	0	0	0.00E+00	3	None

0.7318719	0	0	0.00E+00	3	None
0.7384636	0	0	0.00E+00	3	None
0.738963	0	0	0.00E+00	3	None
0.763099	0	0	0.00E+00	3	None
0.7817184	0	0	0.00E+00	3	None
0.7924715	0	0	0.00E+00	3	None
0.7973723	0	0	0.00E+00	3	None
0.817065	0	0	0.00E+00	3	None
0.8258206	0	0	0.00E+00	3	None
0.8500529	0	0	0.00E+00	3	None
0.9269821	0	0	0.00E+00	3	None
0.9962951	0	0	0.00E+00	3	None
0.9996196	0	0	0.00E+00	3	None

Net Benifit(predict time=5 year)

Thresholds	TPR	FPR	Net Benift	time	model
0.3105849	0.410984435	0.58901556	1.46E-01	5	RiskScore
0.3107291	0.412527907	0.58314309	1.50E-01	5	RiskScore
0.3108211	0.414126539	0.57721545	1.54E-01	5	RiskScore
0.3113224	0.413217844	0.57379514	1.54E-01	5	RiskScore
0.3118866	0.414860691	0.56782329	1.57E-01	5	RiskScore
0.3143975	0.414054147	0.56430083	1.55E-01	5	RiskScore
0.3148419	0.413616513	0.56040946	1.56E-01	5	RiskScore
0.3157166	0.412910739	0.55678623	1.56E-01	5	RiskScore
0.3159902	0.41171489	0.55365308	1.56E-01	5	RiskScore
0.3174713	0.410139733	0.55089923	1.54E-01	5	RiskScore
0.3176215	0.4092112	0.54749876	1.54E-01	5	RiskScore
0.3177016	0.409459629	0.54292132	1.57E-01	5	RiskScore
0.3179311	0.411084977	0.53696697	1.61E-01	5	RiskScore
0.3184902	0.4101359	0.53358704	1.61E-01	5	RiskScore
0.3188592	0.411808166	0.52758577	1.65E-01	5	RiskScore
0.3191154	0.410844402	0.52422053	1.65E-01	5	RiskScore
0.3197065	0.410701352	0.52003458	1.66E-01	5	RiskScore
0.3199839	0.410069297	0.51633763	1.67E-01	5	RiskScore
0.3220413	0.410588815	0.51148911	1.68E-01	5	RiskScore
0.3220891	0.412306084	0.50544283	1.72E-01	5	RiskScore
0.3230303	0.400032731	0.51338718	1.55E-01	5	RiskScore
0.3235659	0.401659834	0.50743107	1.59E-01	5	RiskScore
0.3245618	0.402397852	0.50236405	1.61E-01	5	RiskScore
0.32478	0.4018194	0.4986135	1.62E-01	5	RiskScore
0.3248932	0.401004702	0.49509919	1.63E-01	5	RiskScore
0.3250979	0.402692881	0.48908201	1.67E-01	5	RiskScore
0.3252161	0.402188627	0.48525726	1.68E-01	5	RiskScore
0.3253469	0.401188677	0.48192821	1.69E-01	5	RiskScore
0.3253796	0.400686846	0.47810103	1.70E-01	5	RiskScore
0.3255983	0.392743243	0.48171563	1.60E-01	5	RiskScore
0.325726	0.394423803	0.47570607	1.65E-01	5	RiskScore

0.3257571	0.393103293	0.47269757	1.65E-01	5	RiskScore
0.3263546	0.394840089	0.46663177	1.69E-01	5	RiskScore
0.3264916	0.390448243	0.46669461	1.64E-01	5	RiskScore
0.3267067	0.389986094	0.46282776	1.65E-01	5	RiskScore
0.3270323	0.389435605	0.45904924	1.66E-01	5	RiskScore
0.3271276	0.389542903	0.45461294	1.69E-01	5	RiskScore
0.3272032	0.389453408	0.45037343	1.70E-01	5	RiskScore
0.3272575	0.385099966	0.45039787	1.66E-01	5	RiskScore
0.3276804	0.385232747	0.44593608	1.68E-01	5	RiskScore
0.3282524	0.383566901	0.44327293	1.67E-01	5	RiskScore
0.3284398	0.382681549	0.43982927	1.68E-01	5	RiskScore
0.3284875	0.380957586	0.43722423	1.67E-01	5	RiskScore
0.3286103	0.376307997	0.43754482	1.62E-01	5	RiskScore
0.3293537	0.375286367	0.43423744	1.62E-01	5	RiskScore
0.3301206	0.370577857	0.43461695	1.56E-01	5	RiskScore
0.3303228	0.370477902	0.4303879	1.58E-01	5	RiskScore
0.3305238	0.369410805	0.42712599	1.59E-01	5	RiskScore
0.3312808	0.368514485	0.42369331	1.59E-01	5	RiskScore
0.3317479	0.370173402	0.41770539	1.63E-01	5	RiskScore
0.3318045	0.371910858	0.41163893	1.68E-01	5	RiskScore
0.3318797	0.371799417	0.40742136	1.69E-01	5	RiskScore
0.3330055	0.370647982	0.40424379	1.69E-01	5	RiskScore
0.3331163	0.36996659	0.40059618	1.70E-01	5	RiskScore
0.3334358	0.370686747	0.39554702	1.73E-01	5	RiskScore
0.3337052	0.366186725	0.39571804	1.68E-01	5	RiskScore
0.3338205	0.36012752	0.39744824	1.61E-01	5	RiskScore
0.3338337	0.36192	0.39132675	1.66E-01	5	RiskScore
0.3350576	0.35739523	0.39152252	1.60E-01	5	RiskScore
0.3350712	0.343225341	0.4013634	1.41E-01	5	RiskScore
0.3365389	0.342712345	0.3975474	1.41E-01	5	RiskScore
0.3367327	0.335642471	0.40028826	1.32E-01	5	RiskScore
0.3367922	0.337162522	0.39443921	1.37E-01	5	RiskScore
0.3379115	0.336336721	0.39093601	1.37E-01	5	RiskScore
0.3389489	0.335883568	0.38706016	1.37E-01	5	RiskScore
0.3391591	0.331318355	0.38729636	1.33E-01	5	RiskScore
0.3391676	0.332879956	0.38140576	1.37E-01	5	RiskScore
0.3401459	0.33163844	0.37831827	1.37E-01	5	RiskScore
0.341115	0.331078802	0.3745489	1.37E-01	5	RiskScore
0.3411285	0.330081527	0.37121717	1.38E-01	5	RiskScore
0.3413625	0.331661616	0.36530808	1.42E-01	5	RiskScore
0.3419523	0.331632877	0.36100782	1.44E-01	5	RiskScore
0.3422649	0.330889255	0.35742243	1.45E-01	5	RiskScore
0.3426273	0.330530505	0.35345218	1.46E-01	5	RiskScore
0.344365	0.329656816	0.34999686	1.46E-01	5	RiskScore
0.3443857	0.331259325	0.34406535	1.51E-01	5	RiskScore
0.3449245	0.32990712	0.34108855	1.50E-01	5	RiskScore
0.3458055	0.318915875	0.34775079	1.35E-01	5	RiskScore
0.3461931	0.32040449	0.34193317	1.39E-01	5	RiskScore

0.3464059	0.321977897	0.33603076	1.44E-01	5	RiskScore
0.3464264	0.319905144	0.33377451	1.43E-01	5	RiskScore
0.347128	0.319491532	0.32985912	1.44E-01	5	RiskScore
0.3474402	0.318649642	0.326372	1.45E-01	5	RiskScore
0.3474667	0.320251604	0.32044104	1.50E-01	5	RiskScore
0.3479736	0.321953832	0.3144098	1.54E-01	5	RiskScore
0.3480315	0.323768244	0.30826639	1.59E-01	5	RiskScore
0.3484697	0.32279132	0.30491431	1.60E-01	5	RiskScore
0.3485624	0.322116013	0.30126061	1.61E-01	5	RiskScore
0.348763	0.324002169	0.29504545	1.66E-01	5	RiskScore
0.3488437	0.326026986	0.28869163	1.71E-01	5	RiskScore
0.3496011	0.324407606	0.285982	1.71E-01	5	RiskScore
0.3498917	0.326555525	0.27950508	1.76E-01	5	RiskScore
0.3499847	0.326332402	0.2753992	1.78E-01	5	RiskScore
0.3504967	0.321189488	0.27621311	1.72E-01	5	RiskScore
0.3510332	0.3197708	0.27330279	1.72E-01	5	RiskScore
0.3513561	0.318680633	0.27006396	1.72E-01	5	RiskScore
0.351833	0.319073	0.26534258	1.75E-01	5	RiskScore
0.3519446	0.309082305	0.27100427	1.62E-01	5	RiskScore
0.352001	0.307654081	0.26810349	1.62E-01	5	RiskScore
0.3540594	0.306804538	0.26462403	1.62E-01	5	RiskScore
0.354432	0.290375251	0.27672432	1.38E-01	5	RiskScore
0.3563316	0.289202788	0.27356777	1.38E-01	5	RiskScore
0.3567945	0.289721249	0.26872031	1.41E-01	5	RiskScore
0.3582521	0.285358914	0.26875364	1.35E-01	5	RiskScore
0.3582591	0.285571751	0.2642118	1.38E-01	5	RiskScore
0.3582862	0.283450501	0.26200404	1.37E-01	5	RiskScore
0.3583317	0.282553674	0.25857187	1.38E-01	5	RiskScore
0.358454	0.283783288	0.25301325	1.42E-01	5	RiskScore
0.3594734	0.279254884	0.25321265	1.37E-01	5	RiskScore
0.3598431	0.277363406	0.25077512	1.36E-01	5	RiskScore
0.3610361	0.272167455	0.25164207	1.30E-01	5	RiskScore
0.3622987	0.273445729	0.24603479	1.34E-01	5	RiskScore
0.3623776	0.273191291	0.24196022	1.36E-01	5	RiskScore
0.3630498	0.271683254	0.23913926	1.35E-01	5	RiskScore
0.3630559	0.273005817	0.23348769	1.40E-01	5	RiskScore
0.3638871	0.274430349	0.22773415	1.44E-01	5	RiskScore
0.3639408	0.275972699	0.2218628	1.49E-01	5	RiskScore
0.3644888	0.271608932	0.22189756	1.44E-01	5	RiskScore
0.3644995	0.273289271	0.21588822	1.49E-01	5	RiskScore
0.3646733	0.272655161	0.21219332	1.51E-01	5	RiskScore
0.365266	0.272326742	0.20819274	1.53E-01	5	RiskScore
0.3661561	0.270819091	0.20537139	1.52E-01	5	RiskScore
0.3662622	0.272558183	0.19930329	1.57E-01	5	RiskScore
0.368854	0.274480097	0.19305237	1.62E-01	5	RiskScore
0.3689842	0.274599141	0.18860432	1.64E-01	5	RiskScore
0.3712754	0.27422006	0.1846544	1.65E-01	5	RiskScore
0.3713518	0.269119712	0.18542574	1.60E-01	5	RiskScore

0.372334	0.271215718	0.17900073	1.65E-01	5	RiskScore
0.3724467	0.266674412	0.17921303	1.60E-01	5	RiskScore
0.3726946	0.262272136	0.17928631	1.56E-01	5	RiskScore
0.3729658	0.264639554	0.17258988	1.62E-01	5	RiskScore
0.3740373	0.267348921	0.16555151	1.68E-01	5	RiskScore
0.3746886	0.266193808	0.16237762	1.69E-01	5	RiskScore
0.3760319	0.269276979	0.15496545	1.76E-01	5	RiskScore
0.3776247	0.269901089	0.15001233	1.79E-01	5	RiskScore
0.3782198	0.267402336	0.14818208	1.77E-01	5	RiskScore
0.379037	0.264650814	0.1466046	1.75E-01	5	RiskScore
0.3820333	0.262855015	0.14407139	1.74E-01	5	RiskScore
0.3830669	0.266290869	0.13630653	1.82E-01	5	RiskScore
0.3855023	0.270529123	0.12773928	1.90E-01	5	RiskScore
0.3865621	0.269147991	0.1247914	1.91E-01	5	RiskScore
0.387209	0.245680849	0.14392954	1.55E-01	5	RiskScore
0.388081	0.239441978	0.14583941	1.47E-01	5	RiskScore
0.3881624	0.241047546	0.13990483	1.52E-01	5	RiskScore
0.3891761	0.242826148	0.13379723	1.58E-01	5	RiskScore
0.3897288	0.238272452	0.13402192	1.53E-01	5	RiskScore
0.3906858	0.237999787	0.12996558	1.55E-01	5	RiskScore
0.3907865	0.239955385	0.12368098	1.61E-01	5	RiskScore
0.3909728	0.238232633	0.12107473	1.61E-01	5	RiskScore
0.3913266	0.237392852	0.1175855	1.62E-01	5	RiskScore
0.3938678	0.232269956	0.11837939	1.55E-01	5	RiskScore
0.3991543	0.22942116	0.11689919	1.52E-01	5	RiskScore
0.4002644	0.231424505	0.11056684	1.58E-01	5	RiskScore
0.4003094	0.224368969	0.11329337	1.49E-01	5	RiskScore
0.4015117	0.222747895	0.11058544	1.49E-01	5	RiskScore
0.4048755	0.218378402	0.11062593	1.43E-01	5	RiskScore
0.4082275	0.220525401	0.10414992	1.49E-01	5	RiskScore
0.4132244	0.215972029	0.10437429	1.42E-01	5	RiskScore
0.4134114	0.213507384	0.10250993	1.41E-01	5	RiskScore
0.4136669	0.215962102	0.09572621	1.48E-01	5	RiskScore
0.4215412	0.207337519	0.10002179	1.34E-01	5	RiskScore
0.4236432	0.202803933	0.10022637	1.29E-01	5	RiskScore
0.4270494	0.202939354	0.09576194	1.32E-01	5	RiskScore
0.4341861	0.198147371	0.09622492	1.24E-01	5	RiskScore
0.4361264	0.193796926	0.09624636	1.19E-01	5	RiskScore
0.4362612	0.191273343	0.09444094	1.18E-01	5	RiskScore
0.4362614	0.190605088	0.09078019	1.20E-01	5	RiskScore
0.4370122	0.188864625	0.08819165	1.20E-01	5	RiskScore
0.4425097	0.191284271	0.081443	1.27E-01	5	RiskScore
0.4429907	0.18886886	0.07952941	1.26E-01	5	RiskScore
0.4433255	0.187549294	0.07651997	1.27E-01	5	RiskScore
0.4444792	0.187486748	0.07225351	1.30E-01	5	RiskScore
0.4466024	0.183076565	0.07233469	1.25E-01	5	RiskScore
0.4511967	0.182604706	0.06847754	1.26E-01	5	RiskScore
0.4515817	0.181237939	0.06551531	1.27E-01	5	RiskScore

0.4526428	0.176696377	0.06572787	1.22E-01	5	RiskScore
0.4529389	0.238095238	0	2.38E-01	5	RiskScore
0.4554728	0.233766234	0	2.34E-01	5	RiskScore
0.4613606	0.229437229	0	2.29E-01	5	RiskScore
0.4652488	0.225108225	0	2.25E-01	5	RiskScore
0.4710217	0.220779221	0	2.21E-01	5	RiskScore
0.4719209	0.216450216	0	2.16E-01	5	RiskScore
0.475095	0.212121212	0	2.12E-01	5	RiskScore
0.4837615	0.207792208	0	2.08E-01	5	RiskScore
0.490587	0.203463203	0	2.03E-01	5	RiskScore
0.4943727	0.199134199	0	1.99E-01	5	RiskScore
0.5030363	0.194805195	0	1.95E-01	5	RiskScore
0.5038831	0.19047619	0	1.90E-01	5	RiskScore
0.5057861	0.186147186	0	1.86E-01	5	RiskScore
0.5105096	0.181818182	0	1.82E-01	5	RiskScore
0.5114614	0.177489177	0	1.77E-01	5	RiskScore
0.5175175	0.173160173	0	1.73E-01	5	RiskScore
0.521526	0.168831169	0	1.69E-01	5	RiskScore
0.5218148	0.164502165	0	1.65E-01	5	RiskScore
0.5236663	0.16017316	0	1.60E-01	5	RiskScore
0.5255541	0.155844156	0	1.56E-01	5	RiskScore
0.5365768	0.151515152	0	1.52E-01	5	RiskScore
0.5384862	0.147186147	0	1.47E-01	5	RiskScore
0.5389075	0.142857143	0	1.43E-01	5	RiskScore
0.54013	0.138528139	0	1.39E-01	5	RiskScore
0.5464612	0.134199134	0	1.34E-01	5	RiskScore
0.5510665	0.12987013	0	1.30E-01	5	RiskScore
0.5631931	0.125541126	0	1.26E-01	5	RiskScore
0.5643262	0.121212121	0	1.21E-01	5	RiskScore
0.5782391	0.116883117	0	1.17E-01	5	RiskScore
0.5788434	0.112554113	0	1.13E-01	5	RiskScore
0.5823409	0.108225108	0	1.08E-01	5	RiskScore
0.5887664	0.103896104	0	1.04E-01	5	RiskScore
0.5942409	0.0995671	0	9.96E-02	5	RiskScore
0.6030137	0.095238095	0	9.52E-02	5	RiskScore
0.6257554	0.090909091	0	9.09E-02	5	RiskScore
0.6395396	0.086580087	0	8.66E-02	5	RiskScore
0.6454523	0.082251082	0	8.23E-02	5	RiskScore
0.6584098	0.077922078	0	7.79E-02	5	RiskScore
0.6630001	0.073593074	0	7.36E-02	5	RiskScore
0.6675557	0.069264069	0	6.93E-02	5	RiskScore
0.7288033	0.064935065	0	6.49E-02	5	RiskScore
0.765146	0.060606061	0	6.06E-02	5	RiskScore
0.785458	0.056277056	0	5.63E-02	5	RiskScore
0.7885097	0.051948052	0	5.19E-02	5	RiskScore
0.7973839	0.047619048	0	4.76E-02	5	RiskScore
0.8428399	0.043290043	0	4.33E-02	5	RiskScore
0.8482443	0.038961039	0	3.90E-02	5	RiskScore

0.8903717	0.034632035	0	3.46E-02	5	RiskScore
0.8939937	0.03030303	0	3.03E-02	5	RiskScore
0.9081859	0.025974026	0	2.60E-02	5	RiskScore
0.9143031	0.021645022	0	2.16E-02	5	RiskScore
0.9305771	0.017316017	0	1.73E-02	5	RiskScore
0.9747555	0.012987013	0	1.30E-02	5	RiskScore
0.999618	0.008658009	0	8.66E-03	5	RiskScore
0.9999844	0.004329004	0	4.33E-03	5	RiskScore
0.4082561	0.410984435	0.58901556	4.61E-03	5	Age
0.4116407	0.12981754	0.17321276	8.63E-03	5	Age
0.3832798	0.410984435	0.58901556	4.49E-02	5	Gender
0.4626018	0.152724461	0.15896385	1.59E-02	5	Gender
0.4039391	0.410984435	0.58901556	1.18E-02	5	Grade
0.4079572	0.363787531	0.51067134	1.19E-02	5	Grade
0.4120002	0.194557894	0.24267154	2.45E-02	5	Grade
0.4160679	0.043290043	0	4.33E-02	5	Grade
0.255516	0.410984435	0.58901556	2.09E-01	5	Stage
0.4255714	0.284195597	0.22662691	1.16E-01	5	Stage
0.647108	0.212355569	0.08634573	5.40E-02	5	Stage
0.8587173	0.017316017	0	1.73E-02	5	Stage
0.2626528	0.410984435	0.58901556	2.01E-01	5	T.Stage
0.4250164	0.27345591	0.22870859	1.04E-01	5	T.Stage
0.6340118	0.202044659	0.08366963	5.71E-02	5	T.Stage
0.8388856	0.043290043	0	4.33E-02	5	T.Stage
0.405269	0.410984435	0.58901556	9.61E-03	5	N.Stage
0.6592146	0.017316017	0	1.73E-02	5	N.Stage
0.4012842	0.410984435	0.58901556	1.62E-02	5	M.Stage
0.8666324	0.012987013	0	1.30E-02	5	M.Stage
0	0.410984435	0.58901556	4.11E-01	5	All
0.255516	0.410984435	0.58901556	2.09E-01	5	All
0.2626528	0.410984435	0.58901556	2.01E-01	5	All
0.3105849	0.410984435	0.58901556	1.46E-01	5	All
0.3107291	0.410984435	0.58901556	1.45E-01	5	All
0.3108211	0.410984435	0.58901556	1.45E-01	5	All
0.3113224	0.410984435	0.58901556	1.45E-01	5	All
0.3118866	0.410984435	0.58901556	1.44E-01	5	All
0.3143975	0.410984435	0.58901556	1.41E-01	5	All
0.3148419	0.410984435	0.58901556	1.40E-01	5	All
0.3157166	0.410984435	0.58901556	1.39E-01	5	All
0.3159902	0.410984435	0.58901556	1.39E-01	5	All
0.3174713	0.410984435	0.58901556	1.37E-01	5	All
0.3176215	0.410984435	0.58901556	1.37E-01	5	All
0.3177016	0.410984435	0.58901556	1.37E-01	5	All
0.3179311	0.410984435	0.58901556	1.36E-01	5	All
0.3184902	0.410984435	0.58901556	1.36E-01	5	All
0.3188592	0.410984435	0.58901556	1.35E-01	5	All
0.3191154	0.410984435	0.58901556	1.35E-01	5	All
0.3197065	0.410984435	0.58901556	1.34E-01	5	All

0.3199839	0.410984435	0.58901556	1.34E-01	5	All
0.3220413	0.410984435	0.58901556	1.31E-01	5	All
0.3220891	0.410984435	0.58901556	1.31E-01	5	All
0.3230303	0.410984435	0.58901556	1.30E-01	5	All
0.3235659	0.410984435	0.58901556	1.29E-01	5	All
0.3245618	0.410984435	0.58901556	1.28E-01	5	All
0.32478	0.410984435	0.58901556	1.28E-01	5	All
0.3248932	0.410984435	0.58901556	1.28E-01	5	All
0.3250979	0.410984435	0.58901556	1.27E-01	5	All
0.3252161	0.410984435	0.58901556	1.27E-01	5	All
0.3253469	0.410984435	0.58901556	1.27E-01	5	All
0.3253796	0.410984435	0.58901556	1.27E-01	5	All
0.3255983	0.410984435	0.58901556	1.27E-01	5	All
0.325726	0.410984435	0.58901556	1.26E-01	5	All
0.3257571	0.410984435	0.58901556	1.26E-01	5	All
0.3263546	0.410984435	0.58901556	1.26E-01	5	All
0.3264916	0.410984435	0.58901556	1.25E-01	5	All
0.3267067	0.410984435	0.58901556	1.25E-01	5	All
0.3270323	0.410984435	0.58901556	1.25E-01	5	All
0.3271276	0.410984435	0.58901556	1.25E-01	5	All
0.3272032	0.410984435	0.58901556	1.25E-01	5	All
0.3272575	0.410984435	0.58901556	1.24E-01	5	All
0.3276804	0.410984435	0.58901556	1.24E-01	5	All
0.3282524	0.410984435	0.58901556	1.23E-01	5	All
0.3284398	0.410984435	0.58901556	1.23E-01	5	All
0.3284875	0.410984435	0.58901556	1.23E-01	5	All
0.3286103	0.410984435	0.58901556	1.23E-01	5	All
0.3293537	0.410984435	0.58901556	1.22E-01	5	All
0.3301206	0.410984435	0.58901556	1.21E-01	5	All
0.3303228	0.410984435	0.58901556	1.20E-01	5	All
0.3305238	0.410984435	0.58901556	1.20E-01	5	All
0.3312808	0.410984435	0.58901556	1.19E-01	5	All
0.3317479	0.410984435	0.58901556	1.19E-01	5	All
0.3318045	0.410984435	0.58901556	1.18E-01	5	All
0.3318797	0.410984435	0.58901556	1.18E-01	5	All
0.3330055	0.410984435	0.58901556	1.17E-01	5	All
0.3331163	0.410984435	0.58901556	1.17E-01	5	All
0.3334358	0.410984435	0.58901556	1.16E-01	5	All
0.3337052	0.410984435	0.58901556	1.16E-01	5	All
0.3338205	0.410984435	0.58901556	1.16E-01	5	All
0.3338337	0.410984435	0.58901556	1.16E-01	5	All
0.3350576	0.410984435	0.58901556	1.14E-01	5	All
0.3350712	0.410984435	0.58901556	1.14E-01	5	All
0.3365389	0.410984435	0.58901556	1.12E-01	5	All
0.3367327	0.410984435	0.58901556	1.12E-01	5	All
0.3367922	0.410984435	0.58901556	1.12E-01	5	All
0.3379115	0.410984435	0.58901556	1.10E-01	5	All
0.3389489	0.410984435	0.58901556	1.09E-01	5	All

0.3391591	0.410984435	0.58901556	1.09E-01	5	All
0.3391676	0.410984435	0.58901556	1.09E-01	5	All
0.3401459	0.410984435	0.58901556	1.07E-01	5	All
0.341115	0.410984435	0.58901556	1.06E-01	5	All
0.3411285	0.410984435	0.58901556	1.06E-01	5	All
0.3413625	0.410984435	0.58901556	1.06E-01	5	All
0.3419523	0.410984435	0.58901556	1.05E-01	5	All
0.3422649	0.410984435	0.58901556	1.04E-01	5	All
0.3426273	0.410984435	0.58901556	1.04E-01	5	All
0.344365	0.410984435	0.58901556	1.02E-01	5	All
0.3443857	0.410984435	0.58901556	1.02E-01	5	All
0.3449245	0.410984435	0.58901556	1.01E-01	5	All
0.3458055	0.410984435	0.58901556	9.96E-02	5	All
0.3461931	0.410984435	0.58901556	9.91E-02	5	All
0.3464059	0.410984435	0.58901556	9.88E-02	5	All
0.3464264	0.410984435	0.58901556	9.88E-02	5	All
0.347128	0.410984435	0.58901556	9.78E-02	5	All
0.3474402	0.410984435	0.58901556	9.74E-02	5	All
0.3474667	0.410984435	0.58901556	9.73E-02	5	All
0.3479736	0.410984435	0.58901556	9.66E-02	5	All
0.3480315	0.410984435	0.58901556	9.66E-02	5	All
0.3484697	0.410984435	0.58901556	9.60E-02	5	All
0.3485624	0.410984435	0.58901556	9.58E-02	5	All
0.348763	0.410984435	0.58901556	9.55E-02	5	All
0.3488437	0.410984435	0.58901556	9.54E-02	5	All
0.3496011	0.410984435	0.58901556	9.44E-02	5	All
0.3498917	0.410984435	0.58901556	9.40E-02	5	All
0.3499847	0.410984435	0.58901556	9.38E-02	5	All
0.3504967	0.410984435	0.58901556	9.31E-02	5	All
0.3510332	0.410984435	0.58901556	9.24E-02	5	All
0.3513561	0.410984435	0.58901556	9.19E-02	5	All
0.351833	0.410984435	0.58901556	9.13E-02	5	All
0.3519446	0.410984435	0.58901556	9.11E-02	5	All
0.352001	0.410984435	0.58901556	9.10E-02	5	All
0.3540594	0.410984435	0.58901556	8.81E-02	5	All
0.354432	0.410984435	0.58901556	8.76E-02	5	All
0.3563316	0.410984435	0.58901556	8.49E-02	5	All
0.3567945	0.410984435	0.58901556	8.42E-02	5	All
0.3582521	0.410984435	0.58901556	8.22E-02	5	All
0.3582591	0.410984435	0.58901556	8.22E-02	5	All
0.3582862	0.410984435	0.58901556	8.21E-02	5	All
0.3583317	0.410984435	0.58901556	8.21E-02	5	All
0.358454	0.410984435	0.58901556	8.19E-02	5	All
0.3594734	0.410984435	0.58901556	8.04E-02	5	All
0.3598431	0.410984435	0.58901556	7.99E-02	5	All
0.3610361	0.410984435	0.58901556	7.82E-02	5	All
0.3622987	0.410984435	0.58901556	7.63E-02	5	All
0.3623776	0.410984435	0.58901556	7.62E-02	5	All

0.3630498	0.410984435	0.58901556	7.53E-02	5 All
0.3630559	0.410984435	0.58901556	7.52E-02	5 All
0.3638871	0.410984435	0.58901556	7.40E-02	5 All
0.3639408	0.410984435	0.58901556	7.40E-02	5 All
0.3644888	0.410984435	0.58901556	7.32E-02	5 All
0.3644995	0.410984435	0.58901556	7.31E-02	5 All
0.3646733	0.410984435	0.58901556	7.29E-02	5 All
0.365266	0.410984435	0.58901556	7.20E-02	5 All
0.3661561	0.410984435	0.58901556	7.07E-02	5 All
0.3662622	0.410984435	0.58901556	7.06E-02	5 All
0.368854	0.410984435	0.58901556	6.68E-02	5 All
0.3689842	0.410984435	0.58901556	6.66E-02	5 All
0.3712754	0.410984435	0.58901556	6.32E-02	5 All
0.3713518	0.410984435	0.58901556	6.30E-02	5 All
0.372334	0.410984435	0.58901556	6.16E-02	5 All
0.3724467	0.410984435	0.58901556	6.14E-02	5 All
0.3726946	0.410984435	0.58901556	6.10E-02	5 All
0.3729658	0.410984435	0.58901556	6.06E-02	5 All
0.3740373	0.410984435	0.58901556	5.90E-02	5 All
0.3746886	0.410984435	0.58901556	5.80E-02	5 All
0.3760319	0.410984435	0.58901556	5.60E-02	5 All
0.3776247	0.410984435	0.58901556	5.36E-02	5 All
0.3782198	0.410984435	0.58901556	5.27E-02	5 All
0.379037	0.410984435	0.58901556	5.14E-02	5 All
0.3820333	0.410984435	0.58901556	4.68E-02	5 All
0.3830669	0.410984435	0.58901556	4.53E-02	5 All
0.3832798	0.410984435	0.58901556	4.49E-02	5 All
0.3855023	0.410984435	0.58901556	4.15E-02	5 All
0.3865621	0.410984435	0.58901556	3.98E-02	5 All
0.387209	0.410984435	0.58901556	3.88E-02	5 All
0.388081	0.410984435	0.58901556	3.74E-02	5 All
0.3881624	0.410984435	0.58901556	3.73E-02	5 All
0.3891761	0.410984435	0.58901556	3.57E-02	5 All
0.3897288	0.410984435	0.58901556	3.48E-02	5 All
0.3906858	0.410984435	0.58901556	3.33E-02	5 All
0.3907865	0.410984435	0.58901556	3.32E-02	5 All
0.3909728	0.410984435	0.58901556	3.29E-02	5 All
0.3913266	0.410984435	0.58901556	3.23E-02	5 All
0.3938678	0.410984435	0.58901556	2.82E-02	5 All
0.3991543	0.410984435	0.58901556	1.97E-02	5 All
0.4002644	0.410984435	0.58901556	1.79E-02	5 All
0.4003094	0.410984435	0.58901556	1.78E-02	5 All
0.4012842	0.410984435	0.58901556	1.62E-02	5 All
0.4015117	0.410984435	0.58901556	1.58E-02	5 All
0.4039391	0.410984435	0.58901556	1.18E-02	5 All
0.4048755	0.410984435	0.58901556	1.03E-02	5 All
0.405269	0.410984435	0.58901556	9.61E-03	5 All
0.4079572	0.410984435	0.58901556	5.11E-03	5 All

0.4082275	0.410984435	0.58901556	4.66E-03	5	All
0.4082561	0.410984435	0.58901556	4.61E-03	5	All
0.4116407	0.410984435	0.58901556	-1.12E-03	5	All
0.4120002	0.410984435	0.58901556	-1.73E-03	5	All
0.4132244	0.410984435	0.58901556	-3.82E-03	5	All
0.4134114	0.410984435	0.58901556	-4.14E-03	5	All
0.4136669	0.410984435	0.58901556	-4.57E-03	5	All
0.4160679	0.410984435	0.58901556	-8.71E-03	5	All
0.4215412	0.410984435	0.58901556	-1.82E-02	5	All
0.4236432	0.410984435	0.58901556	-2.20E-02	5	All
0.4250164	0.410984435	0.58901556	-2.44E-02	5	All
0.4255714	0.410984435	0.58901556	-2.54E-02	5	All
0.4270494	0.410984435	0.58901556	-2.80E-02	5	All
0.4341861	0.410984435	0.58901556	-4.10E-02	5	All
0.4361264	0.410984435	0.58901556	-4.46E-02	5	All
0.4362612	0.410984435	0.58901556	-4.48E-02	5	All
0.4362614	0.410984435	0.58901556	-4.48E-02	5	All
0.4370122	0.410984435	0.58901556	-4.62E-02	5	All
0.4425097	0.410984435	0.58901556	-5.65E-02	5	All
0.4429907	0.410984435	0.58901556	-5.75E-02	5	All
0.4433255	0.410984435	0.58901556	-5.81E-02	5	All
0.4444792	0.410984435	0.58901556	-6.03E-02	5	All
0.4466024	0.410984435	0.58901556	-6.44E-02	5	All
0.4511967	0.410984435	0.58901556	-7.33E-02	5	All
0.4515817	0.410984435	0.58901556	-7.40E-02	5	All
0.4526428	0.410984435	0.58901556	-7.61E-02	5	All
0.4529389	0.410984435	0.58901556	-7.67E-02	5	All
0.4554728	0.410984435	0.58901556	-8.17E-02	5	All
0.4613606	0.410984435	0.58901556	-9.35E-02	5	All
0.4626018	0.410984435	0.58901556	-9.61E-02	5	All
0.4652488	0.410984435	0.58901556	-1.01E-01	5	All
0.4710217	0.410984435	0.58901556	-1.13E-01	5	All
0.4719209	0.410984435	0.58901556	-1.15E-01	5	All
0.475095	0.410984435	0.58901556	-1.22E-01	5	All
0.4837615	0.410984435	0.58901556	-1.41E-01	5	All
0.490587	0.410984435	0.58901556	-1.56E-01	5	All
0.4943727	0.410984435	0.58901556	-1.65E-01	5	All
0.5030363	0.410984435	0.58901556	-1.85E-01	5	All
0.5038831	0.410984435	0.58901556	-1.87E-01	5	All
0.5057861	0.410984435	0.58901556	-1.92E-01	5	All
0.5105096	0.410984435	0.58901556	-2.03E-01	5	All
0.5114614	0.410984435	0.58901556	-2.06E-01	5	All
0.5175175	0.410984435	0.58901556	-2.21E-01	5	All
0.521526	0.410984435	0.58901556	-2.31E-01	5	All
0.5218148	0.410984435	0.58901556	-2.32E-01	5	All
0.5236663	0.410984435	0.58901556	-2.37E-01	5	All
0.5255541	0.410984435	0.58901556	-2.41E-01	5	All
0.5365768	0.410984435	0.58901556	-2.71E-01	5	All

0.5384862	0.410984435	0.58901556	-2.76E-01	5	All
0.5389075	0.410984435	0.58901556	-2.77E-01	5	All
0.54013	0.410984435	0.58901556	-2.81E-01	5	All
0.5464612	0.410984435	0.58901556	-2.99E-01	5	All
0.5510665	0.410984435	0.58901556	-3.12E-01	5	All
0.5631931	0.410984435	0.58901556	-3.48E-01	5	All
0.5643262	0.410984435	0.58901556	-3.52E-01	5	All
0.5782391	0.410984435	0.58901556	-3.97E-01	5	All
0.5788434	0.410984435	0.58901556	-3.99E-01	5	All
0.5823409	0.410984435	0.58901556	-4.10E-01	5	All
0.5887664	0.410984435	0.58901556	-4.32E-01	5	All
0.5942409	0.410984435	0.58901556	-4.52E-01	5	All
0.6030137	0.410984435	0.58901556	-4.84E-01	5	All
0.6257554	0.410984435	0.58901556	-5.74E-01	5	All
0.6340118	0.410984435	0.58901556	-6.09E-01	5	All
0.6395396	0.410984435	0.58901556	-6.34E-01	5	All
0.6454523	0.410984435	0.58901556	-6.61E-01	5	All
0.647108	0.410984435	0.58901556	-6.69E-01	5	All
0.6584098	0.410984435	0.58901556	-7.24E-01	5	All
0.6592146	0.410984435	0.58901556	-7.28E-01	5	All
0.6630001	0.410984435	0.58901556	-7.48E-01	5	All
0.6675557	0.410984435	0.58901556	-7.72E-01	5	All
0.7288033	0.410984435	0.58901556	-1.17E+00	5	All
0.765146	0.410984435	0.58901556	-1.51E+00	5	All
0.785458	0.410984435	0.58901556	-1.75E+00	5	All
0.7885097	0.410984435	0.58901556	-1.79E+00	5	All
0.7973839	0.410984435	0.58901556	-1.91E+00	5	All
0.8388856	0.410984435	0.58901556	-2.66E+00	5	All
0.8428399	0.410984435	0.58901556	-2.75E+00	5	All
0.8482443	0.410984435	0.58901556	-2.88E+00	5	All
0.8587173	0.410984435	0.58901556	-3.17E+00	5	All
0.8666324	0.410984435	0.58901556	-3.42E+00	5	All
0.8903717	0.410984435	0.58901556	-4.37E+00	5	All
0.8939937	0.410984435	0.58901556	-4.56E+00	5	All
0.9081859	0.410984435	0.58901556	-5.42E+00	5	All
0.9143031	0.410984435	0.58901556	-5.87E+00	5	All
0.9305771	0.410984435	0.58901556	-7.48E+00	5	All
0.9747555	0.410984435	0.58901556	-2.23E+01	5	All
0.999618	0.410984435	0.58901556	-1.54E+03	5	All
0.9999844	0.410984435	0.58901556	-3.78E+04	5	All
0	0	0	0.00E+00	5	None
0.255516	0	0	0.00E+00	5	None
0.2626528	0	0	0.00E+00	5	None
0.3105849	0	0	0.00E+00	5	None
0.3107291	0	0	0.00E+00	5	None
0.3108211	0	0	0.00E+00	5	None
0.3113224	0	0	0.00E+00	5	None
0.3118866	0	0	0.00E+00	5	None

0.3143975	0	0	0.00E+00	5 None
0.3148419	0	0	0.00E+00	5 None
0.3157166	0	0	0.00E+00	5 None
0.3159902	0	0	0.00E+00	5 None
0.3174713	0	0	0.00E+00	5 None
0.3176215	0	0	0.00E+00	5 None
0.3177016	0	0	0.00E+00	5 None
0.3179311	0	0	0.00E+00	5 None
0.3184902	0	0	0.00E+00	5 None
0.3188592	0	0	0.00E+00	5 None
0.3191154	0	0	0.00E+00	5 None
0.3197065	0	0	0.00E+00	5 None
0.3199839	0	0	0.00E+00	5 None
0.3220413	0	0	0.00E+00	5 None
0.3220891	0	0	0.00E+00	5 None
0.3230303	0	0	0.00E+00	5 None
0.3235659	0	0	0.00E+00	5 None
0.3245618	0	0	0.00E+00	5 None
0.32478	0	0	0.00E+00	5 None
0.3248932	0	0	0.00E+00	5 None
0.3250979	0	0	0.00E+00	5 None
0.3252161	0	0	0.00E+00	5 None
0.3253469	0	0	0.00E+00	5 None
0.3253796	0	0	0.00E+00	5 None
0.3255983	0	0	0.00E+00	5 None
0.325726	0	0	0.00E+00	5 None
0.3257571	0	0	0.00E+00	5 None
0.3263546	0	0	0.00E+00	5 None
0.3264916	0	0	0.00E+00	5 None
0.3267067	0	0	0.00E+00	5 None
0.3270323	0	0	0.00E+00	5 None
0.3271276	0	0	0.00E+00	5 None
0.3272032	0	0	0.00E+00	5 None
0.3272575	0	0	0.00E+00	5 None
0.3276804	0	0	0.00E+00	5 None
0.3282524	0	0	0.00E+00	5 None
0.3284398	0	0	0.00E+00	5 None
0.3284875	0	0	0.00E+00	5 None
0.3286103	0	0	0.00E+00	5 None
0.3293537	0	0	0.00E+00	5 None
0.3301206	0	0	0.00E+00	5 None
0.3303228	0	0	0.00E+00	5 None
0.3305238	0	0	0.00E+00	5 None
0.3312808	0	0	0.00E+00	5 None
0.3317479	0	0	0.00E+00	5 None
0.3318045	0	0	0.00E+00	5 None
0.3318797	0	0	0.00E+00	5 None
0.3330055	0	0	0.00E+00	5 None

0.3331163	0	0	0.00E+00	5 None
0.3334358	0	0	0.00E+00	5 None
0.3337052	0	0	0.00E+00	5 None
0.3338205	0	0	0.00E+00	5 None
0.3338337	0	0	0.00E+00	5 None
0.3350576	0	0	0.00E+00	5 None
0.3350712	0	0	0.00E+00	5 None
0.3365389	0	0	0.00E+00	5 None
0.3367327	0	0	0.00E+00	5 None
0.3367922	0	0	0.00E+00	5 None
0.3379115	0	0	0.00E+00	5 None
0.3389489	0	0	0.00E+00	5 None
0.3391591	0	0	0.00E+00	5 None
0.3391676	0	0	0.00E+00	5 None
0.3401459	0	0	0.00E+00	5 None
0.341115	0	0	0.00E+00	5 None
0.3411285	0	0	0.00E+00	5 None
0.3413625	0	0	0.00E+00	5 None
0.3419523	0	0	0.00E+00	5 None
0.3422649	0	0	0.00E+00	5 None
0.3426273	0	0	0.00E+00	5 None
0.344365	0	0	0.00E+00	5 None
0.3443857	0	0	0.00E+00	5 None
0.3449245	0	0	0.00E+00	5 None
0.3458055	0	0	0.00E+00	5 None
0.3461931	0	0	0.00E+00	5 None
0.3464059	0	0	0.00E+00	5 None
0.3464264	0	0	0.00E+00	5 None
0.347128	0	0	0.00E+00	5 None
0.3474402	0	0	0.00E+00	5 None
0.3474667	0	0	0.00E+00	5 None
0.3479736	0	0	0.00E+00	5 None
0.3480315	0	0	0.00E+00	5 None
0.3484697	0	0	0.00E+00	5 None
0.3485624	0	0	0.00E+00	5 None
0.348763	0	0	0.00E+00	5 None
0.3488437	0	0	0.00E+00	5 None
0.3496011	0	0	0.00E+00	5 None
0.3498917	0	0	0.00E+00	5 None
0.3499847	0	0	0.00E+00	5 None
0.3504967	0	0	0.00E+00	5 None
0.3510332	0	0	0.00E+00	5 None
0.3513561	0	0	0.00E+00	5 None
0.351833	0	0	0.00E+00	5 None
0.3519446	0	0	0.00E+00	5 None
0.352001	0	0	0.00E+00	5 None
0.3540594	0	0	0.00E+00	5 None
0.354432	0	0	0.00E+00	5 None

0.3563316	0	0	0.00E+00	5	None
0.3567945	0	0	0.00E+00	5	None
0.3582521	0	0	0.00E+00	5	None
0.3582591	0	0	0.00E+00	5	None
0.3582862	0	0	0.00E+00	5	None
0.3583317	0	0	0.00E+00	5	None
0.358454	0	0	0.00E+00	5	None
0.3594734	0	0	0.00E+00	5	None
0.3598431	0	0	0.00E+00	5	None
0.3610361	0	0	0.00E+00	5	None
0.3622987	0	0	0.00E+00	5	None
0.3623776	0	0	0.00E+00	5	None
0.3630498	0	0	0.00E+00	5	None
0.3630559	0	0	0.00E+00	5	None
0.3638871	0	0	0.00E+00	5	None
0.3639408	0	0	0.00E+00	5	None
0.3644888	0	0	0.00E+00	5	None
0.3644995	0	0	0.00E+00	5	None
0.3646733	0	0	0.00E+00	5	None
0.365266	0	0	0.00E+00	5	None
0.3661561	0	0	0.00E+00	5	None
0.3662622	0	0	0.00E+00	5	None
0.368854	0	0	0.00E+00	5	None
0.3689842	0	0	0.00E+00	5	None
0.3712754	0	0	0.00E+00	5	None
0.3713518	0	0	0.00E+00	5	None
0.372334	0	0	0.00E+00	5	None
0.3724467	0	0	0.00E+00	5	None
0.3726946	0	0	0.00E+00	5	None
0.3729658	0	0	0.00E+00	5	None
0.3740373	0	0	0.00E+00	5	None
0.3746886	0	0	0.00E+00	5	None
0.3760319	0	0	0.00E+00	5	None
0.3776247	0	0	0.00E+00	5	None
0.3782198	0	0	0.00E+00	5	None
0.379037	0	0	0.00E+00	5	None
0.3820333	0	0	0.00E+00	5	None
0.3830669	0	0	0.00E+00	5	None
0.3832798	0	0	0.00E+00	5	None
0.3855023	0	0	0.00E+00	5	None
0.3865621	0	0	0.00E+00	5	None
0.387209	0	0	0.00E+00	5	None
0.388081	0	0	0.00E+00	5	None
0.3881624	0	0	0.00E+00	5	None
0.3891761	0	0	0.00E+00	5	None
0.3897288	0	0	0.00E+00	5	None
0.3906858	0	0	0.00E+00	5	None
0.3907865	0	0	0.00E+00	5	None

0.3909728	0	0	0.00E+00	5 None
0.3913266	0	0	0.00E+00	5 None
0.3938678	0	0	0.00E+00	5 None
0.3991543	0	0	0.00E+00	5 None
0.4002644	0	0	0.00E+00	5 None
0.4003094	0	0	0.00E+00	5 None
0.4012842	0	0	0.00E+00	5 None
0.4015117	0	0	0.00E+00	5 None
0.4039391	0	0	0.00E+00	5 None
0.4048755	0	0	0.00E+00	5 None
0.405269	0	0	0.00E+00	5 None
0.4079572	0	0	0.00E+00	5 None
0.4082275	0	0	0.00E+00	5 None
0.4082561	0	0	0.00E+00	5 None
0.4116407	0	0	0.00E+00	5 None
0.4120002	0	0	0.00E+00	5 None
0.4132244	0	0	0.00E+00	5 None
0.4134114	0	0	0.00E+00	5 None
0.4136669	0	0	0.00E+00	5 None
0.4160679	0	0	0.00E+00	5 None
0.4215412	0	0	0.00E+00	5 None
0.4236432	0	0	0.00E+00	5 None
0.4250164	0	0	0.00E+00	5 None
0.4255714	0	0	0.00E+00	5 None
0.4270494	0	0	0.00E+00	5 None
0.4341861	0	0	0.00E+00	5 None
0.4361264	0	0	0.00E+00	5 None
0.4362612	0	0	0.00E+00	5 None
0.4362614	0	0	0.00E+00	5 None
0.4370122	0	0	0.00E+00	5 None
0.4425097	0	0	0.00E+00	5 None
0.4429907	0	0	0.00E+00	5 None
0.4433255	0	0	0.00E+00	5 None
0.4444792	0	0	0.00E+00	5 None
0.4466024	0	0	0.00E+00	5 None
0.4511967	0	0	0.00E+00	5 None
0.4515817	0	0	0.00E+00	5 None
0.4526428	0	0	0.00E+00	5 None
0.4529389	0	0	0.00E+00	5 None
0.4554728	0	0	0.00E+00	5 None
0.4613606	0	0	0.00E+00	5 None
0.4626018	0	0	0.00E+00	5 None
0.4652488	0	0	0.00E+00	5 None
0.4710217	0	0	0.00E+00	5 None
0.4719209	0	0	0.00E+00	5 None
0.475095	0	0	0.00E+00	5 None
0.4837615	0	0	0.00E+00	5 None
0.490587	0	0	0.00E+00	5 None

0.4943727	0	0	0.00E+00	5 None
0.5030363	0	0	0.00E+00	5 None
0.5038831	0	0	0.00E+00	5 None
0.5057861	0	0	0.00E+00	5 None
0.5105096	0	0	0.00E+00	5 None
0.5114614	0	0	0.00E+00	5 None
0.5175175	0	0	0.00E+00	5 None
0.521526	0	0	0.00E+00	5 None
0.5218148	0	0	0.00E+00	5 None
0.5236663	0	0	0.00E+00	5 None
0.5255541	0	0	0.00E+00	5 None
0.5365768	0	0	0.00E+00	5 None
0.5384862	0	0	0.00E+00	5 None
0.5389075	0	0	0.00E+00	5 None
0.54013	0	0	0.00E+00	5 None
0.5464612	0	0	0.00E+00	5 None
0.5510665	0	0	0.00E+00	5 None
0.5631931	0	0	0.00E+00	5 None
0.5643262	0	0	0.00E+00	5 None
0.5782391	0	0	0.00E+00	5 None
0.5788434	0	0	0.00E+00	5 None
0.5823409	0	0	0.00E+00	5 None
0.5887664	0	0	0.00E+00	5 None
0.5942409	0	0	0.00E+00	5 None
0.6030137	0	0	0.00E+00	5 None
0.6257554	0	0	0.00E+00	5 None
0.6340118	0	0	0.00E+00	5 None
0.6395396	0	0	0.00E+00	5 None
0.6454523	0	0	0.00E+00	5 None
0.647108	0	0	0.00E+00	5 None
0.6584098	0	0	0.00E+00	5 None
0.6592146	0	0	0.00E+00	5 None
0.6630001	0	0	0.00E+00	5 None
0.6675557	0	0	0.00E+00	5 None
0.7288033	0	0	0.00E+00	5 None
0.765146	0	0	0.00E+00	5 None
0.785458	0	0	0.00E+00	5 None
0.7885097	0	0	0.00E+00	5 None
0.7973839	0	0	0.00E+00	5 None
0.8388856	0	0	0.00E+00	5 None
0.8428399	0	0	0.00E+00	5 None
0.8482443	0	0	0.00E+00	5 None
0.8587173	0	0	0.00E+00	5 None
0.8666324	0	0	0.00E+00	5 None
0.8903717	0	0	0.00E+00	5 None
0.8939937	0	0	0.00E+00	5 None
0.9081859	0	0	0.00E+00	5 None
0.9143031	0	0	0.00E+00	5 None

0.9305771	0	0	0.00E+00	5	None
0.9747555	0	0	0.00E+00	5	None
0.999618	0	0	0.00E+00	5	None
0.9999844	0	0	0.00E+00	5	None
