# Supplementary material for: Inflammatory Response-Related Long Non-Coding RNA Signature Predicts the Prognosis of Hepatocellular Carcinoma
Source: J Oncol. 2022 Mar 17;2022:9917244. doi: 10.1155/2022/9917244 (PMC8947866; doi:10.1155/2022/9917244)
Supplement: Supplementary Materials — Supplementary tables: Supplementary Table 1. Identified inflammatory response-related genes from the Molecular Signatures Database. Supplementary Table 2. The inflammatory response-related DEGs between HCC and noncancerous liver tissues. Supplementary Table 3. The results of univariate Cox regression, LASSO regression, and multivariate Cox regression analysis. Supplementary Table 4. The net benefit of risk score model in DCA analysis. Supplementary Table 5. The results of gene set enrichment analysis. Supplementary Table 6. The immune responses in low- and high-risk groups. [file 9917244.f1.zip › 9917244.f1/Table S5.pdf]

NAME	SIZE	ES	NES
KEGG_OOCYTE_MEIOSIS	112	0.6624955	2.115606
KEGG_UBIQUITIN_MEDIATED_PROTEOLYSIS	134	0.69962215	2.041962
KEGG_PROGESTERONE_MEDIATED_OOCYTE_MATURATION	85	0.6508083	2.0419383
KEGG_CELL_CYCLE	124	0.73831236	2.0406644
KEGG_BASAL_TRANSCRIPTION_FACTORS	35	0.7400221	2.0100958
KEGG_N_GLYCAN_BIOSYNTHESIS	46	0.67688495	1.9854827
KEGG_SELENOAMINO_ACID_METABOLISM	26	0.6581696	1.9847543
KEGG_PURINE_METABOLISM	158	0.5640995	1.9797554
KEGG_RIG_I_LIKE_RECEPTOR_SIGNALING_PATHWAY	71	0.6216107	1.9740378
KEGG_NEUROTROPHIN_SIGNALING_PATHWAY	126	0.6404363	1.9705857
KEGG_ENDOCYTOSIS	181	0.595811	1.9581361
KEGG_NUCLEOTIDE_EXCISION_REPAIR	44	0.71928316	1.9569633
KEGG_ADHERENS_JUNCTION	73	0.65944946	1.9545999
KEGG_SPLICEOSOME	127	0.7542767	1.949555
KEGG_SNARE_INTERACTIONS_IN_VESICULAR_TRANSPORT	38	0.6675923	1.9466406
KEGG_HOMOLOGOUS_RECOMBINATION	28	0.78333443	1.9378208
KEGG_BASE_EXCISION_REPAIR	35	0.72638965	1.9377903
KEGG_PYRIMIDINE_METABOLISM	97	0.6131995	1.9291654
KEGG_INSULIN_SIGNALING_PATHWAY	137	0.5531149	1.9288081
KEGG_RNA_DEGRADATION	59	0.6978642	1.9251618
KEGG_INOSITOL_PHOSPHATE_METABOLISM	54	0.6567466	1.9040831
KEGG_VASOPRESSIN_REGULATED_WATER_REABSORPTION	44	0.6555975	1.8917382
KEGG_MTOR_SIGNALING_PATHWAY	52	0.6269871	1.8850543
KEGG_LYSOSOME	121	0.5850764	1.8806491
KEGG_SMALL_CELL_LUNG_CANCER	84	0.60162914	1.8710742
KEGG_NON_SMALL_CELL_LUNG_CANCER	54	0.6158745	1.865066
KEGG_NOTCH_SIGNALING_PATHWAY	47	0.6495572	1.8634309
KEGG_GLYCOSYLPHOSPHATIDYLINOSITOL_GPI_ANCHOR_BIOSYNTHESIS	25	0.6918692	1.8579806
KEGG_P53_SIGNALING_PATHWAY	68	0.5599155	1.8403447
KEGG_SPHINGOLIPID_METABOLISM	39	0.6124906	1.8391871
KEGG_EPITHELIAL_CELL_SIGNALING_IN_HELICOBACTER_PYLORI_INFECTION	68	0.5869512	1.8378565
KEGG_WNT_SIGNALING_PATHWAY	150	0.5635554	1.8266078
KEGG_PATHWAYS_IN_CANCER	325	0.5316778	1.8218701
KEGG_RENAL_CELL_CARCINOMA	70	0.60396427	1.8198363
KEGG_ERBB_SIGNALING_PATHWAY	87	0.57701474	1.8094954
KEGG_PROSTATE_CANCER	89	0.5664932	1.8082001
KEGG_AMINOACYL_TRNA_BIOSYNTHESIS	41	0.68039846	1.8027006
KEGG_MISMATCH_REPAIR	23	0.7602231	1.7962892
KEGG_DNA_REPLICATION	36	0.78262717	1.7902265
KEGG_ENDOMETRIAL_CANCER	52	0.59688437	1.7874465
KEGG_GLIOMA	65	0.5620725	1.787172
KEGG_CHRONIC_MYELOID_LEUKEMIA	73	0.6124952	1.7824203
KEGG_PHOSPHATIDYLINOSITOL_SIGNALING_SYSTEM	76	0.5895815	1.7648176
KEGG_COLORECTAL_CANCER	62	0.5952998	1.7647237
KEGG_LONG_TERM_POTENTIATION	70	0.54283875	1.7572465
KEGG_THYROID_CANCER	29	0.61852103	1.7572324
KEGG_REGULATION_OF_AUTOPHAGY	35	0.5649515	1.7542275

KEGG_PANCREATIC_CANCER	70	0.6024046	1.7541561
KEGG_GLYCOSPHINGOLIPID_BIOSYNTHESIS_GANGLIO_SERIES	15	0.6642768	1.7508739
KEGG_VIBRIO_CHOLERAE_INFECTION	54	0.55359614	1.7374707
KEGG_PATHOGENIC_ESCHERICHIA_COLI_INFECTION	56	0.59870756	1.7299137
KEGG_REGULATION_OF_ACTIN_CYTOSKELETON	213	0.52010864	1.726523
KEGG_LYSINE_DEGRADATION	44	0.57107425	1.7207237
KEGG_MELANOGENESIS	101	0.5138626	1.7173152
KEGG_APOPTOSIS	87	0.5487454	1.7170392
KEGG_FC_GAMMA_R_MEDIATED_PHAGOCYTOSIS	96	0.5815464	1.7156612
KEGG_TIGHT_JUNCTION	132	0.5037549	1.7076752
KEGG_AXON_GUIDANCE	129	0.5309281	1.7010524
KEGG_RIBOFLAVIN_METABOLISM	16	0.6333337	1.6975933
KEGG_RNA_POLYMERASE	28	0.6466209	1.6756047
KEGG_CYTOSOLIC_DNA_SENSING_PATHWAY	55	0.5473379	1.6647785
KEGG_TGF_BETA_SIGNALING_PATHWAY	85	0.5335589	1.6538563
KEGG_MAPK_SIGNALING_PATHWAY	267	0.4735812	1.6511292
KEGG_ACUTE_MYELOID_LEUKEMIA	57	0.5501514	1.6483382
KEGG_GLYCEROPHOSPHOLIPID_METABOLISM	77	0.4616319	1.6465411
KEGG_BLADDER_CANCER	42	0.5487105	1.6453047
KEGG_CYSTEINE_AND_METHIONINE_METABOLISM	34	0.5153346	1.6422017
KEGG_GAP_JUNCTION	90	0.5276503	1.6363022
KEGG_VEGF_SIGNALING_PATHWAY	76	0.49966478	1.6316439
KEGG_T_CELL_RECEPTOR_SIGNALING_PATHWAY	108	0.5596939	1.6272769
KEGG_FC_EPSILON_RI_SIGNALING_PATHWAY	79	0.49717262	1.6183743
KEGG_DORSO_VENTRAL_AXIS_FORMATION	24	0.6022423	1.615599
KEGG_AMINO_SUGAR_AND_NUCLEOTIDE_SUGAR_METABOLISM	43	0.50552946	1.599891
KEGG_TOLL_LIKE_RECEPTOR_SIGNALING_PATHWAY	102	0.50276446	1.5903633
KEGG_FRUCTOSE_AND_MANNOSE_METABOLISM	33	0.48261058	1.5849419
KEGG_NOD_LIKE_RECEPTOR_SIGNALING_PATHWAY	62	0.5435348	1.5789636
KEGG_BASAL_CELL_CARCINOMA	55	0.50294167	1.5783118
KEGG_GNRH_SIGNALING_PATHWAY	101	0.47856563	1.5526774
KEGG_OTHER_GLYCAN_DEGRADATION	16	0.60078484	1.5451264
KEGG_MELANOMA	71	0.46739423	1.5350335
KEGG_LONG_TERM_DEPRESSION	70	0.46564743	1.5269681
KEGG_FOCAL_ADHESION	199	0.47046733	1.5152496
KEGG_GLUTATHIONE_METABOLISM	49	0.45621035	1.5109098
KEGG_NATURAL_KILLER_CELL_MEDIATED_CYTOTOXICITY	132	0.4886119	1.4932142
KEGG_ADIPOCYTOKINE_SIGNALING_PATHWAY	67	0.4347667	1.4916856
KEGG_LEUKOCYTE_TRANSENDOTHELIAL_MIGRATION	116	0.4528137	1.4804002
KEGG_HEDGEHOG_SIGNALING_PATHWAY	56	0.4746879	1.4795777
KEGG_GLYCOSAMINOGLYCAN_BIOSYNTHESIS_KERATAN_SULFATE	15	0.5910311	1.479355
KEGG_JAK_STAT_SIGNALING_PATHWAY	155	0.44068196	1.4621392
KEGG_B_CELL_RECEPTOR_SIGNALING_PATHWAY	75	0.49840856	1.4357129
KEGG_TYPE_II_DIABETES_MELLITUS	47	0.44901586	1.4322686
KEGG_PROTEASOME	46	0.5753682	1.4247403
KEGG_GLYCEROLIPID_METABOLISM	49	0.39335725	1.4093114
KEGG_ARRHYTHMOGENIC_RIGHT_VENTRICULAR_CARDIOMYOPATHY_ARVC	74	0.4512155	1.405866
KEGG_GLYCOSAMINOGLYCAN_DEGRADATION	21	0.5010851	1.3968394

KEGG_HUNTINGTONS_DISEASE	180	0.42171416	1.39409
KEGG_GLYCOSPHINGOLIPID_BIOSYNTHESIS_LACTO_AND_NEOLACTO_SERIES	26	0.4758294	1.3883597
KEGG_CHEMOKINE_SIGNALING_PATHWAY	188	0.4260471	1.3477087
KEGG_PANTOTHENATE_AND_COA_BIOSYNTHESIS	16	0.47668642	1.3470564
KEGG_ABC_TRANSPORTERS	44	0.41616246	1.3461795
KEGG_AMYOTROPHIC_LATERAL_SCLEROSIS_ALS	53	0.3877835	1.3351145
KEGG_GLYCOSAMINOGLYCAN_BIOSYNTHESIS_HEPARAN_SULFATE	26	0.47651455	1.3190988
KEGG_GLYCOSAMINOGLYCAN_BIOSYNTHESIS_CHONDROITIN_SULFATE	22	0.5014298	1.3093481
KEGG_ALDOSTERONE_REGULATED_SODIUM_REABSORPTION	42	0.40281674	1.2764667
KEGG_CALCIUM_SIGNALING_PATHWAY	177	0.35466552	1.2738997
KEGG_ONE_CARBON_POOL_BY_FOLATE	17	0.4410563	1.2677425
KEGG_ETHER_LIPID_METABOLISM	33	0.39628458	1.2674743
KEGG_LEISHMANIA_INFECTION	70	0.4645345	1.2605143
KEGG_GALACTOSE_METABOLISM	26	0.41065925	1.2495698
KEGG_PROTEIN_EXPORT	24	0.4621027	1.2438889
KEGG_HYPERTROPHIC_CARDIOMYOPATHY_HCM	83	0.392766	1.233202
KEGG_PENTOSE_PHOSPHATE_PATHWAY	27	0.40463695	1.2176644
KEGG_VASCULAR_SMOOTH_MUSCLE_CONTRACTION	114	0.3662516	1.2142007
KEGG_NICOTINATE_AND_NICOTINAMIDE_METABOLISM	24	0.39178497	1.2050511
KEGG_TASTE_TRANSDUCTION	51	0.37340045	1.1964461
KEGG_O_GLYCAN_BIOSYNTHESIS	30	0.4330592	1.1835132
KEGG_ALZHEIMERS_DISEASE	165	0.34913084	1.1717271
KEGG_HISTIDINE_METABOLISM	29	0.3535755	1.0770209
KEGG_SYSTEMIC_LUPUS_ERYTHEMATOSUS	135	0.3272815	1.0742121
KEGG_DILATED_CARDIOMYOPATHY	90	0.33504212	1.0390266
KEGG_VIRAL_MYOCARDITIS	68	0.35947734	1.0300405
KEGG_MATURITY_ONSET_DIABETES_OF_THE_YOUNG	25	0.35491747	1.0194427
KEGG_ANTIGEN_PROCESSING_AND_PRESENTATION	81	0.36594248	0.998762
KEGG_PORPHYRIN_AND_CHLOROPHYLL_METABOLISM	41	0.32974333	0.9980218
KEGG_CYTOKINE_CYTOKINE_RECEPTOR_INTERACTION	264	0.290536	0.9578531
KEGG_OLFACTORY_TRANSDUCTION	385	0.20661671	0.9437751
KEGG_PRIMARY_IMMUNODEFICIENCY	35	0.41287127	0.93257546
KEGG_DRUG_METABOLISM_OTHER_ENZYMES	51	0.27626657	0.9021483
KEGG_ECM_RECEPTOR_INTERACTION	84	0.28752813	0.8934859
KEGG_HEMATOPOIETIC_CELL_LINEAGE	85	0.29664364	0.86540455
KEGG_GLYCOLYSIS_GLUCONEOGENESIS	62	0.25173742	0.8553796
KEGG_RENIN_ANGIOTENSIN_SYSTEM	17	0.333327	0.852037
KEGG_NEUROACTIVE_LIGAND_RECEPTOR_INTERACTION	271	0.22143953	0.83266664
KEGG_STEROID_BIOSYNTHESIS	17	0.36245003	0.83083427
KEGG_PENTOSE_AND_GLUCURONATE_INTERCONVERSIONS	28	0.30055237	0.8273299
KEGG_CARDIAC_MUSCLE_CONTRACTION	78	0.25710502	0.826952
KEGG_CELL_ADHESION_MOLECULES_CAMS	131	0.26320654	0.8251094
KEGG_GLYOXYLATE_AND_DICARBOXYLATE_METABOLISM	16	0.28885505	0.8052129
KEGG_STARCH_AND_SUCROSE_METABOLISM	52	0.23953079	0.7698259
KEGG_RIBOSOME	88	0.3833859	0.7688863
KEGG_INTESTINAL_IMMUNE_NETWORK_FOR_IGA_PRODUCTION	46	0.3100843	0.7627643
KEGG_TYPE_I_DIABETES_MELLITUS	41	0.31423166	0.7553553
KEGG_BIOSYNTHESIS_OF_UNSATURATED_FATTY_ACIDS	22	0.2733508	0.7416762

KEGG_AUTOIMMUNE_THYROID_DISEASE	50	0.25467703	0.6503473
KEGG_ALLOGRAFT_REJECTION	35	0.29809695	0.64515394
KEGG_GRAFT_VERSUS_HOST_DISEASE	37	0.28724983	0.6286367
KEGG_ASTHMA	28	0.2490651	0.61784536
KEGG_COMPLEMENT_AND_COAGULATION_CASCADES	69	-0.8063401	-2.2686536
KEGG_PRIMARY_BILE_ACID_BIOSYNTHESIS	16	-0.813327	-1.788986
KEGG_FATTY_ACID_METABOLISM	42	-0.72239643	-1.7383212
KEGG_TRYPTOPHAN_METABOLISM	40	-0.5586971	-1.5848372
KEGG_VALINE_LEUCINE_AND_ISOLEUCINE_DEGRADATION	44	-0.6569646	-1.5733049
KEGG_GLYCINE_SERINE_AND_THREONINE_METABOLISM	31	-0.5827948	-1.5131673
KEGG_BUTANOATE_METABOLISM	34	-0.56418645	-1.4837382
KEGG_PPAR_SIGNALING_PATHWAY	69	-0.46188417	-1.4694742
KEGG_RETINOL_METABOLISM	64	-0.4905285	-1.4195887
KEGG_PROPANOATE_METABOLISM	33	-0.52601105	-1.341479
KEGG_BETA_ALANINE_METABOLISM	22	-0.52667224	-1.3281809
KEGG_DRUG_METABOLISM_CYTOCHROME_P450	71	-0.45329368	-1.3187491
KEGG_ARGININE_AND_PROLINE_METABOLISM	54	-0.39677155	-1.3019826
KEGG_ALANINE_ASPARTATE_AND_GLUTAMATE_METABOLISM	32	-0.40547788	-1.3015177
KEGG_LINOLEIC_ACID_METABOLISM	29	-0.39308026	-1.1928253
KEGG_PHENYLALANINE_METABOLISM	18	-0.43433046	-1.1539719
KEGG_ARACHIDONIC_ACID_METABOLISM	58	-0.30309832	-1.0978489
KEGG_PROXIMAL_TUBULE_BICARBONATE_RECLAMATION	23	-0.35663843	-1.0610273
KEGG_METABOLISM_OF_XENOBIOTICS_BY_CYTOCHROME_P450	69	-0.35098773	-1.0540184
KEGG_TYROSINE_METABOLISM	42	-0.3265491	-1.034027
KEGG_PRION_DISEASES	35	-0.32075977	-1.0107154
KEGG_STEROID_HORMONE_BIOSYNTHESIS	55	-0.3279169	-0.98870355
KEGG_PEROXISOME	78	-0.3219846	-0.9667502
KEGG_CITRATE_CYCLE_TCA_CYCLE	31	-0.3702301	-0.9517462
KEGG_PYRUVATE_METABOLISM	40	-0.30847457	-0.93884003
KEGG_PARKINSONS_DISEASE	128	-0.31040677	-0.93255067
KEGG_OXIDATIVE_PHOSPHORYLATION	131	-0.29279396	-0.8560544
KEGG_NITROGEN_METABOLISM	23	-0.28967395	-0.8296224
KEGG_TERPENOID_BACKBONE_BIOSYNTHESIS	15	-0.30033937	-0.7484924
KEGG_ALPHA_LINOLENIC_ACID_METABOLISM	19	-0.23337853	-0.67396176
KEGG_ASCORBATE_AND_ALDARATE_METABOLISM	25	-0.25758806	-0.6376537

NOM p-val	FDR q-val	FWER p-val	RANK AT MAX	LEADING EDGE
0	0.005083333	0.002	7352	tags=54%, list=13%, signal=61%
0	0.01628314	0.012	10871	tags=69%, list=19%, signal=86%
0	0.010855427	0.012	11753	tags=59%, list=21%, signal=74%
0	0.00814157	0.012	7690	tags=72%, list=14%, signal=83%
0	0.00998052	0.019	13656	tags=86%, list=24%, signal=113%
0	0.010842638	0.024	7480	tags=59%, list=13%, signal=68%
0	0.009293689	0.024	6592	tags=54%, list=12%, signal=61%
0	0.008754781	0.026	9744	tags=44%, list=17%, signal=53%
0	0.008885265	0.029	10273	tags=45%, list=18%, signal=55%
0	0.008107264	0.03	10078	tags=56%, list=18%, signal=68%
0	0.010231994	0.038	15934	tags=61%, list=28%, signal=85%
0	0.009511049	0.039	11946	tags=80%, list=21%, signal=101%
0	0.008952707	0.041	12198	tags=64%, list=22%, signal=82%
0	0.009336685	0.045	7808	tags=75%, list=14%, signal=87%
0	0.008714238	0.045	12968	tags=68%, list=23%, signal=89%
0	0.009051457	0.051	7492	tags=79%, list=13%, signal=91%
0	0.008519018	0.051	10063	tags=71%, list=18%, signal=87%
0	0.008527607	0.052	9873	tags=55%, list=17%, signal=66%
0	0.008078786	0.052	10159	tags=44%, list=18%, signal=53%
0	0.007780332	0.053	9986	tags=69%, list=18%, signal=84%
0.001988072	0.009820959	0.069	11087	tags=61%, list=20%, signal=76%
0.002	0.010557283	0.076	8569	tags=55%, list=15%, signal=64%
0	0.012004442	0.084	16360	tags=69%, list=29%, signal=97%
0.001949318	0.012133443	0.088	17529	tags=68%, list=31%, signal=98%
0	0.012795515	0.096	9202	tags=50%, list=16%, signal=60%
0.004016064	0.012952801	0.102	14918	tags=63%, list=26%, signal=85%
0	0.012878159	0.103	10766	tags=60%, list=19%, signal=74%
0.002028398	0.013228926	0.108	14076	tags=80%, list=25%, signal=106%
0.001968504	0.015817	0.129	9881	tags=47%, list=17%, signal=57%
0.001980198	0.01532775	0.129	15216	tags=67%, list=27%, signal=91%
0.005714286	0.015003168	0.129	10577	tags=50%, list=19%, signal=61%
0	0.016463581	0.145	11748	tags=49%, list=21%, signal=62%
0	0.016664099	0.147	14947	tags=51%, list=26%, signal=69%
0.005780347	0.016598728	0.149	15475	tags=63%, list=27%, signal=86%
0	0.017749274	0.158	8879	tags=46%, list=16%, signal=54%
0.001992032	0.017443988	0.158	14918	tags=57%, list=26%, signal=78%
0.008048289	0.01798264	0.165	15446	tags=88%, list=27%, signal=121%
0.008016032	0.018703233	0.175	7814	tags=78%, list=14%, signal=91%
0.006048387	0.019250564	0.183	7906	tags=81%, list=14%, signal=94%
0	0.019564504	0.186	14918	tags=62%, list=26%, signal=84%
0.003968254	0.019087322	0.186	8879	tags=43%, list=16%, signal=51%
0.004081633	0.01938082	0.193	14918	tags=66%, list=26%, signal=89%
0.007952286	0.023049101	0.237	11414	tags=54%, list=20%, signal=68%
0.006012024	0.022549825	0.237	15475	tags=65%, list=27%, signal=89%
0.003984064	0.023667723	0.252	12777	tags=50%, list=23%, signal=65%
0.005952381	0.023153208	0.252	9820	tags=52%, list=17%, signal=63%
0	0.023330685	0.257	13936	tags=43%, list=25%, signal=57%

0.007905139	0.022866663	0.257	15782	tags=66%, list=28%, signal=91%
0.008403362	0.02300114	0.263	9827	tags=53%, list=17%, signal=65%
0.007858546	0.025717832	0.296	20935	tags=74%, list=37%, signal=118%
0.009615385	0.027036155	0.311	14875	tags=66%, list=26%, signal=90%
0.007889546	0.027398637	0.319	17237	tags=56%, list=30%, signal=81%
0.008298756	0.028084666	0.326	6082	tags=43%, list=11%, signal=48%
0.002066116	0.028358718	0.33	10518	tags=41%, list=19%, signal=50%
0.005847953	0.02786466	0.33	11753	tags=49%, list=21%, signal=62%
0.011976048	0.027771283	0.334	20414	tags=80%, list=36%, signal=125%
0.004040404	0.029134115	0.35	13107	tags=44%, list=23%, signal=57%
0.007843138	0.030558486	0.366	16209	tags=56%, list=29%, signal=78%
0.011472276	0.030991782	0.376	15670	tags=56%, list=28%, signal=78%
0.010288066	0.03672726	0.414	7859	tags=54%, list=14%, signal=62%
0.022088353	0.03926945	0.437	15160	tags=44%, list=27%, signal=60%
0.023622047	0.042470083	0.461	15934	tags=53%, list=28%, signal=74%
0.007984032	0.04289576	0.471	14496	tags=46%, list=26%, signal=62%
0.012096774	0.04355427	0.475	16190	tags=60%, list=29%, signal=84%
0.003944773	0.043638404	0.48	16815	tags=55%, list=30%, signal=78%
0.01984127	0.04332021	0.485	14872	tags=52%, list=26%, signal=71%
0.014028057	0.04345232	0.492	9825	tags=50%, list=17%, signal=60%
0.021653544	0.04491935	0.507	14875	tags=52%, list=26%, signal=71%
0.00998004	0.046142213	0.52	15363	tags=50%, list=27%, signal=69%
0.03088803	0.0473773	0.535	9794	tags=44%, list=17%, signal=54%
0.01764706	0.050362278	0.559	22015	tags=70%, list=39%, signal=114%
0.033333335	0.05058236	0.566	9005	tags=46%, list=16%, signal=54%
0.024590164	0.05667947	0.611	17116	tags=58%, list=30%, signal=83%
0.033663366	0.06028755	0.636	11900	tags=41%, list=21%, signal=52%
0.01632653	0.061817925	0.642	17116	tags=67%, list=30%, signal=96%
0.038229376	0.06378175	0.65	17261	tags=63%, list=31%, signal=90%
0.02366864	0.06329568	0.651	10518	tags=38%, list=19%, signal=47%
0.025896415	0.07461971	0.698	16551	tags=51%, list=29%, signal=73%
0.032520324	0.077842966	0.706	13628	tags=63%, list=24%, signal=82%
0.027290449	0.08141224	0.721	21307	tags=56%, list=38%, signal=90%
0.024193548	0.08512621	0.739	21192	tags=63%, list=37%, signal=100%
0.06420233	0.09026032	0.762	17681	tags=50%, list=31%, signal=73%
0.029350106	0.09158043	0.768	14414	tags=41%, list=26%, signal=55%
0.07984032	0.10061689	0.788	17166	tags=47%, list=30%, signal=67%
0.024793388	0.100187585	0.79	10273	tags=33%, list=18%, signal=40%
0.07450981	0.10596912	0.806	20414	tags=56%, list=36%, signal=88%
0.04347826	0.105259635	0.806	15514	tags=41%, list=27%, signal=57%
0.06374502	0.10420975	0.807	13831	tags=60%, list=24%, signal=79%
0.085436895	0.11338526	0.836	18341	tags=46%, list=32%, signal=69%
0.091816366	0.1293993	0.862	9695	tags=37%, list=17%, signal=45%
0.070564516	0.13017586	0.864	22122	tags=51%, list=39%, signal=84%
0.13429752	0.13482785	0.876	12644	tags=50%, list=22%, signal=64%
0.03950104	0.14438623	0.889	17940	tags=41%, list=32%, signal=60%
0.09746589	0.14552471	0.893	18483	tags=53%, list=33%, signal=78%
0.09142857	0.14992301	0.897	13353	tags=48%, list=24%, signal=62%

0.14457831	0.15062673	0.897	10432 tags=29%, list=18%, signal=36%
0.10557769	0.15380313	0.904	14550 tags=46%, list=26%, signal=62%
0.16733871	0.18620606	0.932	17261 tags=44%, list=31%, signal=63%
0.11485148	0.18497176	0.933	18793 tags=69%, list=33%, signal=103%
0.10224949	0.183766	0.933	18358 tags=45%, list=32%, signal=67%
0.0862069	0.19149095	0.939	13001 tags=32%, list=23%, signal=42%
0.12115385	0.2036889	0.948	17127 tags=50%, list=30%, signal=72%
0.16054158	0.21058044	0.953	22671 tags=73%, list=40%, signal=121%
0.14078675	0.23902711	0.969	9881 tags=24%, list=17%, signal=29%
0.13238288	0.23911598	0.97	16119 tags=34%, list=29%, signal=48%
0.17463617	0.24268293	0.972	15297 tags=59%, list=27%, signal=81%
0.15127702	0.24073818	0.972	21192 tags=61%, list=37%, signal=97%
0.23505977	0.24559873	0.974	14355 tags=46%, list=25%, signal=61%
0.16488223	0.25406066	0.978	22135 tags=58%, list=39%, signal=95%
0.21616162	0.25687036	0.979	22313 tags=71%, list=39%, signal=117%
0.19183673	0.26488683	0.983	15475 tags=37%, list=27%, signal=51%
0.20654397	0.27780536	0.988	14393 tags=44%, list=25%, signal=60%
0.23217922	0.27880117	0.989	4249 tags=20%, list=8%, signal=22%
0.1912046	0.2847685	0.991	15948 tags=42%, list=28%, signal=58%
0.21802935	0.29100066	0.993	14186 tags=31%, list=25%, signal=42%
0.2813765	0.3012243	0.993	10718 tags=37%, list=19%, signal=45%
0.2834008	0.31166977	0.993	10929 tags=25%, list=19%, signal=31%
0.35670102	0.4185267	0.998	5797 tags=21%, list=10%, signal=23%
0.37601626	0.41831967	0.998	15100 tags=39%, list=27%, signal=52%
0.3909465	0.45905536	0.999	18483 tags=40%, list=33%, signal=59%
0.46015936	0.4672336	0.999	14801 tags=32%, list=26%, signal=44%
0.4069264	0.478025	0.999	22785 tags=48%, list=40%, signal=80%
0.47470817	0.50157535	1	15715 tags=27%, list=28%, signal=38%
0.4520548	0.49861258	1	19644 tags=41%, list=35%, signal=64%
0.4834308	0.54953504	1	22358 tags=41%, list=40%, signal=67%
0.6099585	0.5651152	1	36296 tags=43%, list=64%, signal=120%
0.553816	0.57701874	1	24078 tags=57%, list=43%, signal=99%
0.56713426	0.61662215	1	12179 tags=25%, list=22%, signal=32%
0.57760316	0.624287	1	18483 tags=35%, list=33%, signal=51%
0.5882353	0.66057456	1	21804 tags=41%, list=39%, signal=67%
0.68454933	0.6702855	1	12764 tags=29%, list=23%, signal=37%
0.6659878	0.6701657	1	10182 tags=18%, list=18%, signal=22%
0.8220859	0.6928064	1	30363 tags=58%, list=54%, signal=125%
0.63434345	0.69013363	1	18692 tags=41%, list=33%, signal=62%
0.6620825	0.69025534	1	12179 tags=29%, list=22%, signal=36%
0.6858847	0.6858537	1	14785 tags=24%, list=26%, signal=33%
0.62643677	0.6833481	1	19864 tags=34%, list=35%, signal=53%
0.71666664	0.7071846	1	5405 tags=19%, list=10%, signal=21%
0.78043914	0.753983	1	17385 tags=31%, list=31%, signal=44%
0.71428573	0.7500143	1	28782 tags=69%, list=51%, signal=141%
0.6882353	0.75336546	1	33317 tags=74%, list=59%, signal=180%
0.71259844	0.75901824	1	29960 tags=56%, list=53%, signal=119%
0.7717172	0.7726661	1	29739 tags=59%, list=53%, signal=125%

0.79651165	0.8839098	1	29960 tags=44%, list=53%, signal=94%
0.8032129	0.8837845	1	29960 tags=57%, list=53%, signal=122%
0.7968442	0.89592326	1	32085 tags=62%, list=57%, signal=144%
0.8557114	0.9006258	1	34864 tags=71%, list=62%, signal=186%
0	0	0	1509 tags=62%, list=3%, signal=64%
0.003809524	0.0738875	0.195	2786 tags=63%, list=5%, signal=66%
0.02008032	0.08009421	0.281	1552 tags=55%, list=3%, signal=56%
0.06358381	0.2409628	0.63	1189 tags=35%, list=2%, signal=36%
0.07569721	0.21028136	0.657	3014 tags=55%, list=5%, signal=58%
0.09803922	0.26418778	0.767	3209 tags=58%, list=6%, signal=62%
0.09883721	0.27345276	0.809	1189 tags=41%, list=2%, signal=42%
0.05973025	0.26053515	0.829	1939 tags=38%, list=3%, signal=39%
0.1242485	0.30510134	0.88	2921 tags=42%, list=5%, signal=44%
0.20662768	0.4022251	0.95	2211 tags=45%, list=4%, signal=47%
0.18164437	0.38639462	0.956	2211 tags=55%, list=4%, signal=57%
0.18940938	0.36855796	0.963	4152 tags=52%, list=7%, signal=56%
0.15810277	0.36615938	0.97	2211 tags=37%, list=4%, signal=39%
0.1325536	0.34065217	0.97	1776 tags=50%, list=3%, signal=52%
0.2153558	0.485785	0.992	4152 tags=45%, list=7%, signal=48%
0.30120483	0.5186899	0.997	3039 tags=44%, list=5%, signal=47%
0.2893701	0.5858358	0.999	790 tags=21%, list=1%, signal=21%
0.34860557	0.6173571	1	1880 tags=35%, list=3%, signal=36%
0.422	0.5974447	1	1380 tags=29%, list=2%, signal=30%
0.4026975	0.6012105	1	3336 tags=40%, list=6%, signal=43%
0.45528457	0.6114494	1	603 tags=26%, list=1%, signal=26%
0.44294003	0.61896455	1	3218 tags=31%, list=6%, signal=33%
0.47184467	0.62485975	1	1497 tags=32%, list=3%, signal=33%
0.5048356	0.6218489	1	1904 tags=39%, list=3%, signal=40%
0.5107632	0.6164705	1	1189 tags=28%, list=2%, signal=28%
0.50863725	0.6012003	1	2832 tags=34%, list=5%, signal=36%
0.5868726	0.69225264	1	2832 tags=34%, list=5%, signal=36%
0.7165049	0.70731986	1	2314 tags=35%, list=4%, signal=36%
0.7245509	0.80035514	1	79 tags=13%, list=0%, signal=13%
0.92730844	0.8750043	1	535 tags=16%, list=1%, signal=16%
0.8425358	0.8875634	1	2211 tags=24%, list=4%, signal=25%
