# Supplementary material for: Inflammatory Response-Related Long Non-Coding RNA Signature Predicts the Prognosis of Hepatocellular Carcinoma
Source: J Oncol. 2022 Mar 17;2022:9917244. doi: 10.1155/2022/9917244 (PMC8947866; doi:10.1155/2022/9917244)
Supplement: Supplementary Materials — Supplementary tables: Supplementary Table 1. Identified inflammatory response-related genes from the Molecular Signatures Database. Supplementary Table 2. The inflammatory response-related DEGs between HCC and noncancerous liver tissues. Supplementary Table 3. The results of univariate Cox regression, LASSO regression, and multivariate Cox regression analysis. Supplementary Table 4. The net benefit of risk score model in DCA analysis. Supplementary Table 5. The results of gene set enrichment analysis. Supplementary Table 6. The immune responses in low- and high-risk groups. [file 9917244.f1.zip › 9917244.f1/Table S6.pdf]

immunological response	model	pvalue
B cell_TIMER	RiskScore	0.0374257
T cell CD4+_TIMER	RiskScore	0.0285796
Neutrophil_TIMER	RiskScore	8.91E-05
Macrophage_TIMER	RiskScore	2.32E-06
Myeloid dendritic cell_TIMER	RiskScore	0.0035737
B cell plasma_CIBERSORT	RiskScore	0.0009842
T cell CD4+ memory resting_CIBERSORT	RiskScore	9.64E-07
T cell CD4+ memory activated_CIBERSORT	RiskScore	1.15E-05
T cell follicular helper_CIBERSORT	RiskScore	0.007218
T cell regulatory (Tregs)_CIBERSORT	RiskScore	0.0195682
Monocyte_CIBERSORT	RiskScore	0.0306264
Macrophage M0_CIBERSORT	RiskScore	2.46E-07
Eosinophil_CIBERSORT	RiskScore	0.0309667
B cell plasma_CIBERSORT-ABS	RiskScore	0.0004736
T cell CD4+ memory resting_CIBERSORT-ABS	RiskScore	0.0280864
T cell CD4+ memory activated_CIBERSORT-ABS	RiskScore	1.19E-05
T cell follicular helper_CIBERSORT-ABS	RiskScore	0.0002784
T cell regulatory (Tregs)_CIBERSORT-ABS	RiskScore	0.0001912
NK cell activated_CIBERSORT-ABS	RiskScore	0.0288403
Macrophage M0_CIBERSORT-ABS	RiskScore	2.46E-09
Macrophage M2_CIBERSORT-ABS	RiskScore	0.0270313
B cell_QUANTISEQ	RiskScore	0.0007791
Macrophage M1_QUANTISEQ	RiskScore	0.0051351
Macrophage M2_QUANTISEQ	RiskScore	1.08E-05
Monocyte_QUANTISEQ	RiskScore	2.49E-05
T cell CD4+ (non-regulatory)_QUANTISEQ	RiskScore	0.0455515
T cell CD8+_QUANTISEQ	RiskScore	0.0003445
T cell regulatory (Tregs)_QUANTISEQ	RiskScore	5.78E-06
uncharacterized cell_QUANTISEQ	RiskScore	9.57E-06
T cell_MCPCOUNTER	RiskScore	0.020763
B cell_MCPCOUNTER	RiskScore	0.0414083
Monocyte_MCPCOUNTER	RiskScore	2.96E-05
Macrophage/Monocyte_MCPCOUNTER	RiskScore	2.96E-05
B cell_XCELL	RiskScore	3.58E-05
T cell CD4+ memory_XCELL	RiskScore	0.0001327
T cell CD4+ effector memory_XCELL	RiskScore	0.0022291
T cell CD8+ central memory_XCELL	RiskScore	0.005065
Class-switched memory B cell_XCELL	RiskScore	0.0009743
Common lymphoid progenitor_XCELL	RiskScore	1.19E-06
Common myeloid progenitor_XCELL	RiskScore	0.0471479
Myeloid dendritic cell_XCELL	RiskScore	0.0095691
Endothelial cell_XCELL	RiskScore	3.52E-20
Cancer associated fibroblast_XCELL	RiskScore	6.84E-05
Granulocyte-monocyte progenitor_XCELL	RiskScore	1.11E-07
Hematopoietic stem cell_XCELL	RiskScore	1.27E-17
Macrophage_XCELL	RiskScore	1.05E-05
Macrophage M1_XCELL	RiskScore	0.0075898

Macrophage M2_XCELL	RiskScore	1.84E-07
Mast cell_XCELL	RiskScore	4.39E-05
T cell CD4+ Th2_XCELL	RiskScore	1.27E-12
stroma score_XCELL	RiskScore	8.85E-19
microenvironment score_XCELL	RiskScore	1.27E-08
Macrophage_EPIC	RiskScore	4.49E-12
uncharacterized cell_EPIC	RiskScore	1.92E-12
